# The genome of the Antarctic-endemic copepod, *Tigriopus kingsejongensis*

**DOI:** 10.1093/gigascience/giw010

**Published:** 2017-01-07

**Authors:** Seunghyun Kang, Do-Hwan Ahn, Jun Hyuck Lee, Sung Gu Lee, Seung Chul Shin, Jungeun Lee, Gi-Sik Min, Hyoungseok Lee, Hyun-Woo Kim, Sanghee Kim, Hyun Park

**Affiliations:** 1 Unit of Polar Genomics, Korea Polar Research Institute, Yeonsu-gu, Incheon, South Korea; 2 Polar Sciences, University of Science & Technology, Yuseong-gu, Daejeon, South Korea; 3 Department of Biological Sciences, Inha University, Incheon, South Korea; 4 Department of Marine Biology, Pukyong National University, Busan, South Korea; 5 Division of Polar Life Sciences, Korea Polar Research Institute, Yeonsu-gu, Incheon, South Korea

**Keywords:** Copepoda, Genome, Antarctic, Adaptation, *Tigriopus*

## Abstract

**Background:** The Antarctic intertidal zone is continuously subjected to extremely fluctuating biotic and abiotic stressors. The West Antarctic Peninsula is the most rapidly warming region on Earth. Organisms living in Antarctic intertidal pools are therefore interesting for research into evolutionary adaptation to extreme environments and the effects of climate change.

**Findings:** We report the whole genome sequence of the Antarctic-endemic harpacticoid copepod *Tigriopus kingsejongensi*. The 37 Gb raw DNA sequence was generated using the Illumina Miseq platform. Libraries were prepared with 65-fold coverage and a total length of 295 Mb. The final assembly consists of 48 368 contigs with an N50 contig length of 17.5 kb, and 27 823 scaffolds with an N50 contig length of 159.2 kb. A total of 12 772 coding genes were inferred using the MAKER annotation pipeline. Comparative genome analysis revealed that *T. kingsejongensis*-specific genes are enriched in transport and metabolism processes. Furthermore, rapidly evolving genes related to energy metabolism showed positive selection signatures.

**Conclusions:** The *T. kingsejongensis* genome provides an interesting example of an evolutionary strategy for Antarctic cold adaptation, and offers new genetic insights into Antarctic intertidal biota.

## Data description

Approximately 12 000 species have been described in the diverse copepod subclass [1, 2]. These species dominate the zooplankton community, contributing about 70% of total zooplankton biomass [3], and are an important link between phytoplankton and higher trophic levels in the marine meiobenthic food web [4]. Harpacticoid copepods of the genus *Tigriopus* Norman 1868 are dominant members of shallow supratidal rock pools, distributed worldwide among habitats that vary widely in salinity, temperature, desiccation risk, and UV radiation. They are a model system in investigations of osmoregulation [5], temperature adaptation [6, 7] and environmental toxicology [8]. With publically available copepod genome resources (e.g., *Tigriopus californicus* [9], *T. japonicus* [10], *Eurytemora affinis* [11] and salmon louse *Lepeophtheirus salmonis* [12]), it is now possible to explore their fundamental biological processes and physiological responses to diverse environments.

Antarctica is not only an extreme habitat for extant organisms, but also a model for research on evolutionary adaptations to cold environments [13, 14]. The Antarctic intertidal zone, particularly in the Western Antarctic Peninsula region, is one of the most extreme, yet fastest warming environments on Earth. Thus, it is a potential barometer for global climate change [15]. Antarctic intertidal species that have evolved stenothermal phenotypes through adaptation to year-round extreme cold may now face extinction by global warming. The response of these species to further warming in Western Antarctica is of serious concern; however, to date, few studies have focused on Antarctic intertidal zone species.

First described in 2014, *T. kingsejongensis* was recognized as a new species endemic to a rock pool in the Antarctic Peninsula. It is extremely cold-tolerant and can survive in frozen sea water [16]. Compared to the congener *T. japonicus*, which is found in coastal areas of the Yellow Sea, morphological differences of this species include increased numbers of caudal setae in nauplii, an optimal growth temperature of approximately 8 °C, and differing developmental characteristics. *Tigriopus kingsejongensis* has evolved to overcome the unique environmental constraints of Antarctica, therefore providing an ideal experimental model for extreme habitat research. This species may represent a case of rapid speciation, since the intertidal zone on King George Island and the surrounding areas did not exist 10 000 years ago [17]. *Tigriopus kingsejongensis* likely evolved as a distinct species within this relatively short time period. Thus, interspecies and intraspecies comparative analyses of Antarctic *Tigriopus* species will help to define the trajectory of adaptation to the Antarctic environment, and also provide insights into the genetic basis of *Tigriopus* divergence and evolution.

### Library construction and sequencing

**Figure 1.**
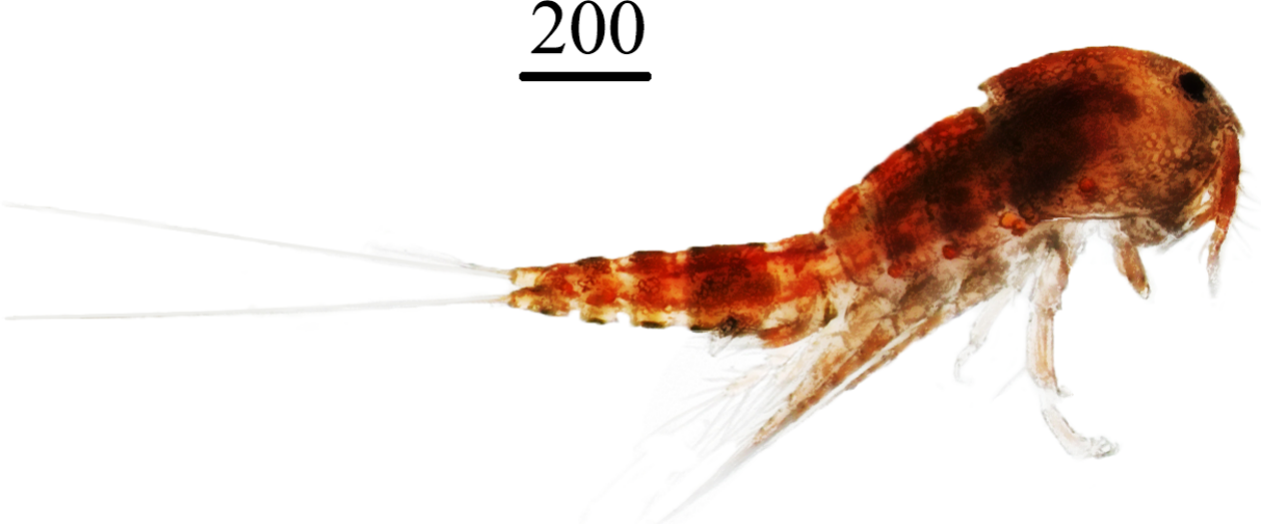
Photograph of an adult *Tigriopus kingsejongensis* specimen (scale bar = 200 μm)

*Tigriopus kingsejongensis* specimens were collected using hand-nets from tidal pools in Potter Cove, near King Sejong Station, on the northern Antarctic Peninsula (62°14^΄^S, 58°47^΄^W) (Fig. 1 and Fig. S1) in January 2013. The water temperature was 1.6 ± 0.8 °C during this month. High molecular weight genomic DNA from pooled *T. kingsejongensis* was extracted using the DNeasy Blood and Tissue Kit (Qiagen, Venlo, The Netherlands). For Illumina Miseq sequencing, four library types were constructed with 350, 400, 450, and 500 bp for paired-end libraries, and 3 kb and 8 kb for mate-pair libraries, prepared using the standard Illumina sample preparation methods (Table 1). All sequencing processes were performed according to the manufacturer's instructions (Illumina, Carlsbad, USA).

**Table 1:** DNA library statistics

Library		Reads (n)	Average	Sequences	Reads Average		Sequences
			length	(bp) (n)	(trimmed) (n)	length	(trimmed) (n)
Paired-end	Sum	99 710 266		29 271 916 613	65 644 374		14 668 956 871
	350S1	6 661 392	300	2 005 078 992	4 446 394	233	1 034 231 244
	350S2	4 933 058	265	1 311 700 122	4 618 711	211	975 471 763
	400S1	65 668 598	300	19 766 247 998	36 863 154	228	8 397 426 481
	450S1	3 418 988	300	1 029 115 388	2 812 455	230	646 302 159
	450S2	8 009 162	245	1 968 652 020	7 660 814	199	1 527 566 312
	500S1	11 019 068	289	3 191 122 093	9 242 846	226	2 087 958 911
Mate-Paired	Sum	103 373 998		7 753 049 850	73 515 391		5 169 006 268
	3KS1	8 374 238	75	628 067 850	6 745 546	73	493 099 413
	3KS2	9 250 994	75	693 824 550	5 281 513	65	344 618 723
	3KS3	51 349 594	75	3 851 219 550	39 147 167	72	2 816 638 666
	3KS4	3 063 232	75	229 742 400	1 740 986	65	112 554 745
	8KS1	9 847 636	75	738 572 700	7 887 612	73	572 246 251
	8KS2	16 322 038	75	1 224 152 850	9 653 293	65	630 842 698
	8KS3	5 166 266	75	387 469 950	3 059 274	65	199 005 774
Total		203 084 264		37 024 966 463	139 159 765		19 837 963 139
Coverage (folds)				120.7			64.7

RNA was prepared from pooled *T. kingsejongensis* and *T. japonicus* specimens from two different temperature experiments (4 °C and 15 °C) using the RNeasy Mini Kit (Qiagen). For Illumina Miseq sequencing, subsequent experiments were carried out according to the manufacturer's instructions (Illumina). The *de novo* transcriptome assembly was performed with CLC Genomics Workbench (Qiagen), setting the minimum allowed contig length to 200 nucleotides. The assembly process generated 40 172 contigs with a maximum length of 23 942 bp and an N50 value of 1093 bp. Generated contigs were used as reference sequences to map trimmed reads, and fold-changes in expression for each gene were calculated with a significance threshold of *P* ≤ 0.05 using the CLC Genomics Workbench (Tables 2 and 3).

**Table 2:** Transcriptome sequencing and assembly analysis for *Tigriopus japonicus*

**Sequencing**	
Total reads (n)	37 956 160
Total bases (n)	7 714 415 316
Trimmed reads (n)	35 577 636
Trimmed bases (n)	5 989 188 343
**Assembly**	
Contigs (n)	40 172
Total contig length (bases)	28 850 726
N50 contig length (bases)	1093
Max scaffold length (bases)	23 942
**Annotation**	
With BLAST results	20 392
Without BLAST hits	7090
With mapping results	8172
Annotated sequences	4518

**Table 3:** RNA-seq statistics analysis for *Tigriopus kingsejongensis*

	Temperature	
	4 °C	15 °C
Total reads (n)	15 786 118	16 417 072
Total bases (n)	3 567 662 668	3 763 295 032
Trimmed reads (n)	14 845 103	15 388 513
Trimmed bases (n)	2 761 189 158	2 833 805 442

### Genome assembly

**Figure 2.**
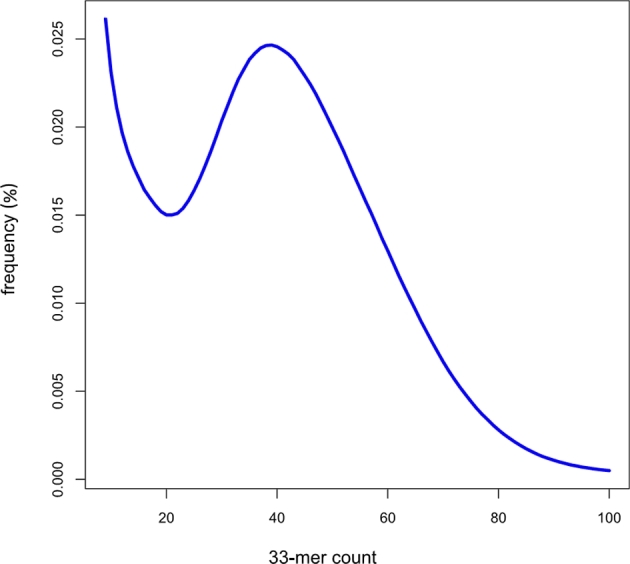
Estimation of the *Tigriopus kingsejongensis* genome size based on 33-mer analysis. X-axis represents the depth (peak at 39×) and the y-axis represents the proportion. Genome size was estimated to be 298 Mb (total k-mer number/volume peak)

**Figure 3.**
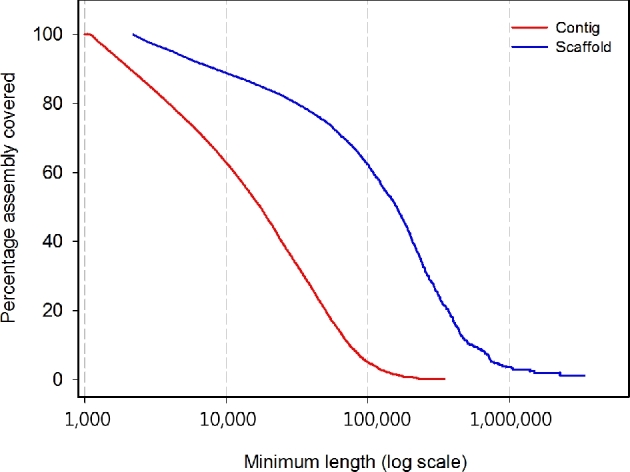
Scaffold and contig size distributions of *Tigriopus kingsejongensis.* The percentage of the assembly included (y-axis) in contigs or scaffolds of a minimum size (x-axis, log scale) is shown for the contig (red) and scaffold (blue)

First, k-mer analysis was conducted using jellyfish 2.2.5 [18] to estimate the genome size from DNA paired-end libraries. The estimated genome size was 298 Mb, with the main peak at a depth of ∼39× (Fig. 2). Then, assemblies were performed using a Celera Assembler with Illumina short reads [19]. Prior to assembly, Illumina reads were trimmed using the FASTX-Toolkit [20] with parameters −t 20, −l 70 and −Q 33, after which a paired sequence from trimmed Illumina reads was selected. Finally, trimmed Illumina reads with 65-fold coverage (insert sizes 350, 400, 450, and 500 bp) were obtained and converted to the FRG file format (required by the Celera Assembler) using FastqToCA. Assembly was performed on a 96-processor workstation with Intel Xeon X7460 2.66 GHz processors and 1 Tb random access memory (RAM) with the following parameters: overlapper = ovl, unitigger = bogart, utgGraphErrorRate = 0.03, utgGraphErrorLimit = 2.5, utgMergeErrorRate = 0.030, utgMergeErrorLimit = 3.25, ovlErrorRate = 0.1, cnsErrorRate = 0.1, cgwErrorRate = 0.1, merSize  =  22, and doOverlapBasedTrimming = 1. The initial Celera assembly was 305 Mb, had an N50 contig size of 17 566 bp, and a maximum contig size of 349.5 kb. Scaffolding was completed using the SSPACE 2.0 scaffolder using mate-paired data [21]. Subsequently, we closed gaps using Gapfiller version 1.9 with 65× trimmed Illumina reads with default settings [22]. *De novo* assembly of 203 million reads from paired-end and mate-paired libraries yielded a draft assembly (65-fold coverage) with a total length of 295 Mb, and contig and scaffold N50 sizes of 17.6 kb and 159.2 kb, respectively (Table 4 and Fig. 3).

**Table 4:** Genome assembly statistics

Type	Parameter	Assembly size according to Celera Assembler
Scaffold	Total scaffold length (bases)	295 233 602
	Gap size (bases)	10 474 460
	Scaffolds (n)	11 558
	N50 scaffold length (bases)	159 218
	Max scaffold length (bases)	3 401 446
Contig	Total contig length (bases)	305 712 242
	Contigs (n)	48 368
	N50 contig length (bases)	17 566
	Max contig length (bases)	349 507

### Annotation

MAKER, a portable and easily configurable genome annotation pipeline, was used to annotate the genome [23]. Repetitive elements were identified using RepeatMasker [24]. This masked genome sequence was used with SNAP software [25] for *ab initio* gene prediction, after which alignment of expressed sequence tags (ESTs) with BLASTn [26] and protein information from tBLASTx [26] were included. The *de novo* repeat library of *T. kingsejongensis* from RepeatModeler was used for RepeatMasker; proteins from five species with data from *Drosophila melanogaster, Daphnia pulex, T. japonicus*, and *T. californicus* were included in the analysis. RNA-seq-based gene prediction, data were aligned against the assembled genome using TopHat [27], and Cufflinks [28] was used to predict cDNAs from the resultant data. Next, MAKER polished the alignments using the program Exonerate [29], which provided integrated information to synthesize SNAP annotation. Considering all information, MAKER then selected and revised the final gene model. A total of 12 772 genes were predicted in *T. kingsejongensis* using MAKER. Annotated genes contained an average of 4.6 exons, with an average mRNA length of 1090 bp. Additionally, 12 562 of 12 772 genes were assigned preliminary functions based on automated annotation using Blast2GO (Ver. 2.6.0) [30] (Figs. S2 and S3) with homology sequences from the SwissProt [31], TrEMBL, National Center for Biotechnology Information (NCBI) non-redudant protein databases [32] and REVIGO software was used to cluster related GO terms according to *P*-value [33]. Infernal version 1.1 [34] and covariance models (CMs) from the Rfam database [35] were used to identify other non-coding RNAs in the *T. kingsejongensis* scaffold. Putative tRNA genes were identified using tRNAscan-SE [36] (Table S1), which uses a CM that scores candidates based on their sequence and predicted secondary structures.

Non-gap sequences occupied 284.8 Mb (96.5%), and simple sequence repeats (SSRs) amounted to 1.2 Mb (0.4%) (Table S2). Transposable elements (TEs) comprised 6.5 Mb; roughly 2.3% of the assembled genome (Tables S2 and S3). On the basis of homology and *ab initio* gene prediction, the *T. kingsejongensis* genome contained 12 772 protein-coding genes (Table 5). By assessing the quality of the 12 772 annotated gene models, 11 686 protein-coding genes (91.5%) were supported by RNA-seq data, of which 7325 (63%) were similar to proteins from other species. To estimate genome assembly and annotation completeness, Core Eukaryotic Genes Mapping Approach (CEGMA) [37] and Benchmarking Universal Single-Copy Orthologs (BUSCO) [38] analysis was used (Table 6). The CEGMA report revealed that 193 of 248 CEGMA score genes were fully annotated (77.8% completeness), and 206 of 248 genes were partially annotated (83% completeness). BUSCO, a similar approach used for lineage-specific profile libraries such as eukaryotes, metazoans, and arthropods, revealed 71% complete and 6% partial Metazoan orthologous gene sets in our assembly; using an arthropod gene set, only 61.1% complete and 10.7% partial genes were assigned. CEGMA and BUSCO gene sets largely comprised insects; other non-insect arthropod genomes obtained similarly low assignment scores. Overall, the *T. kingsejongenesis* genome was moderately complete in non-dipteran arthropod genomes.

**Table 5:** *Tigriopus kingsejongensis* genes: general statistics

Genes (n)	12 772
Gene length sum (bp)	82 293 116
Exons per genes (n)	4.6
mRNA length sum (bp)	43 306 342
Average mRNA length (bp)	1090
Number of tRNA	1393
Number of rRNA	215

**Table 6:** *Tigriopus kingsejongensis* genome completeness reports with the other arthropod genomes

	*Tigriopus*	*Daphnia*	*Ixodes*	*Mesobuthus*	*Strigamia*	*Tetranychus*	*Drosophila*	*Aedes*
Species	*kingsejongensis*	*pulex*	*scapularis*	*martensii*	*maritima*	*urticae*	*melanogaster*	*aegypti*
Assembly	This study	GCA_000187875.1	GCA_000208615.1	GCA_000484575.1	Smar1.22	GCA_000239435.1	Dmel_r5.55	AaegL3
Sample type	genome	genome	genome	genome	genome	genome	genome	genome
CEGMA^a^	83/77.8	99.2/98.8	79.8/41.9^g^	57.3/24.2^g^	95.1^f^	98.0/95.2^g^	100/100	99.2/83.5
BUSCO^b^	61.1 [10.5], 10.7, 28.1	83 [3.9], 11, 5.1^e^	68.9 [2.4], 21.0, 10.1^g^	34.4 [4.0], 23.0, 42.7^g^	84 [5.9], 12, 3.2^e^	68.8 [5.8], 9.9, 21.3^g^	98 [6.4], 0.6, 0.3^e^	86 [13], 10, 3.2 ^e^
BUSCO^c^	70.9 [13.6], 6.0, 23.0							
BUSCO^d^	67.1 [16.8], 5.1, 27.7							

^a^248 CEGMA genes found/complete

^b^BUSCO Arthropods complete [duplicated], fragmented, missing

^c^BUSCO Metazoa complete [duplicated], fragmented, missing

^d^BUSCO Eukaryotes complete [duplicated], fragmented, missing

^e^[38]

^f^[39]

^g^[47]

### Gene families

Orthologous groups were identified from 11 species (*T. kingsejongensis, Aedes aegypti, D. melanogaster, Ixodes scapularis, Mesobuthus martensii, Strigamia martima, Tetranychus urticae, D. pulex, Homo sapiens, Ciona intestinalis*, and *Caenorhabditis elegans*) (Table 7) using OrthoMCL [40] with standard parameters and options; transcript variants other than the longest translation forms were removed. For *T. kingsejongensis*, the coding sequence from the MAKER annotation pipeline was used. The 1:1:1 single-copy orthologous genes were subjected to phylogenetic construction and divergence time estimation. Protein-coding genes were aligned using the Probabilistic Alignment Kit (PRANK) with the codon alignment option [41], and poorly aligned sequences with gaps were removed using Gblock under the codon model [42]. A maximum likelihood phylogenetic tree was constructed using RAxML with 1000 bootstrap values [43] and calibrated the divergence time between species with TimeTree [44]. Finally, the average gene gain/loss rate along the given phylogeny was identified using CAFÉ 3.1 [45].

**Table 7:** Summary of orthologous gene clusters in 11 representative species

Species	Source of data	No. of coding genes	No. of gene families	No. of genes in gene families	No. of orphan genes	No. of unique gene families	Average No. of genes in gene families
*Aedes aegypti*	Ensembl genome 25	15 797	7958	12 792	7839	854	1.61
*Caenorhabditis elegans*	Ensembl gene 78	20 447	6536	13 737	13 911	1528	2.10
*Ciona intestinalis*	Ensembl gene 78	16 671	7017	9058	9654	503	1.29
*Daphnia pulex*	Ensembl genome 25	30 590	6710	8362	7208	368	1.25
*Drosophila melanogaster*	Ensembl gene 78	13 918	9673	21 917	20 917	2408	2.27
*Homo sapiens*	Ensembl gene 78	20 300	8696	17 186	11 604	1065	1.98
*Ixodes scapularis*	Ensembl genome 25	20 486	8097	11 277	12 389	873	1.39
*Mesobuthus martensii*	http://lifecenter.sgst.cn/main/en/scorpion.jsp	32 016	8389	19 961	23 627	2276	2.38
*Strigamia maritima*	Ensembl genome 25	14 992	7727	11 012	7265	583	1.43
*Tetranychus urticae*	Ensembl genome 25	18 224	6602	11 788	11 622	939	1.79
*Tigriopus kingsejongensis*	this study	12 772	6205	8813	6567	649	1.42

**Figure 4.**
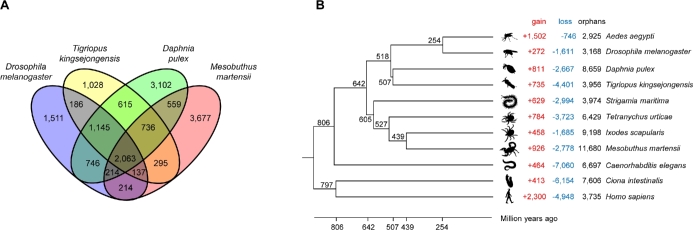
Comparative genome analyses of the *T. kingsejongensis* genome. A. Venn diagram of orthologous gene clusters between four arthropod lineages. B. Gene family gain-and-loss analysis. The number of gained gene families (red), lost gene families (blue) and orphan gene families (black) are indicated for each species. Time lines specify divergence times between the lineages.

Orthologous gene clusters were constructed using four arthropod species (Antarctic copepod, *T. kingsejongensis*; scorpion, *M. martensii*; fruit fly, *D. melanogaster*, and water flea, *D. pulex*) to compare genomic features and adaptive divergence. In total, 2063 gene families are shared by all four species, and 1028 genes are *T. kingsejongensis-*specific. *T. kingsejongensis* shares 4559 (73.5%) gene families with *D. pulex*, which belongs to the same crustacean lineage, Vericrustacea; 3531 (56.9%) with *D. melanogaster*; and 3231 (52.1%) with *M. martensii* (Fig. 4A). Gene Ontology (GO) analysis revealed the 1028 *T. kingsejongensis*-specific genes are enriched in transport (single-organism transport, GO:0044765; transmembrane transport, GO:0055085; ion transport, GO:0006811; cation transport, GO:0006812) and single-organism metabolic processes (GO:0044710) (Tables S4 and S5).

Subsequently, gene gain-and-loss was analyzed in 11 representative species: *T. kingsejongensis* gained 735 and lost 4401 gene families (Fig. 4B). This species exhibits a gene family turnover of 5136, the largest value among the eight arthropods. The second largest value was obtained from *T. uticae* and the third from *M. martensii*. Non-insect arthropod genomes were relatively poorly assigned with CEGMA or BUSCO sets (Table 6). The assignment reports of these largely insect-based gene sets tend to have low assignment scores in non-insect or non-dipteran genomes [38, 46, 47]. This implies that careful examination of gene family turnover is needed in non-insect arthropod genomes, as well as globally approved arthropod orthologous gene sets.

Analysis of gene family expansion and contraction in *T. kingsejongensis* (Tables S6–S9) revealed 232 significantly expanded gene families, which are significantly overrepresented in amino acid and carbohydrate metabolism pathways, according to the Kyoto Encyclopedia of Genes and Genomes (KEGG) [48].

### Genome evolution

Adaptive functional divergence caused by natural selection is commonly estimated based on the ratio of nonsynonymous (*dN*) to synonymous (*dS*) mutations. To estimate *dN, dS*, the average *dN*/*dS* ratio (*w*), and lineage-specific positively selected genes (PSGs) in *T. kingsejongensis* and *T. japonicus*, protein-coding genes from *T. japonicus* were added to define orthologous gene families among four species (*T. kingsejongensis, T. japonicus, D. pulex*, and *D. melanogaster*) using the program OrthoMCL with the same conditions previously described. We identified 2937 orthologous groups shared by all four species; single-copy gene families were used to construct a phylogenetic tree and estimate the time since divergence using the methods described above. Each of the identified orthologous genes was aligned using PRANK, and poorly aligned sequences with gaps were removed using Gblock. Alignments with less than 40% identity and genes shorter than 150 bp were eliminated in subsequent procedures. The values of *dN, dS* and *w* were estimated from each gene using the Codeml program implemented in the Phylogenetic Analysis by Maximum Likelihood (PAML) package with the free-ratio model [49] under F3×4 codon frequencies; orthologs with *w* ≤ 5 and *dS* ≤ 3 were retained [50]. To examine the accelerated nonsynonymous divergence in either the *T. kingsejongensis* or *T. japonicus* lineages, a binomial test [51] was used to determine GO categories with at least 20 orthologous genes. To define PSGs in *T. kingsejongensis* and *T. japonicus*, basic and branch-site models were applied, and Likelihood Ratio Tests (LRTs) were used to remove genes under relaxation of selective pressure. To investigate the functional categories and pathways enriched in PSGs, the Database for Annotation, Visualization and Integrated Discovery (DAVID) Functional Annotation [52] was used with Fisher's exact test (cutoff: *P* ≤ 0.05).

**Figure 5.**
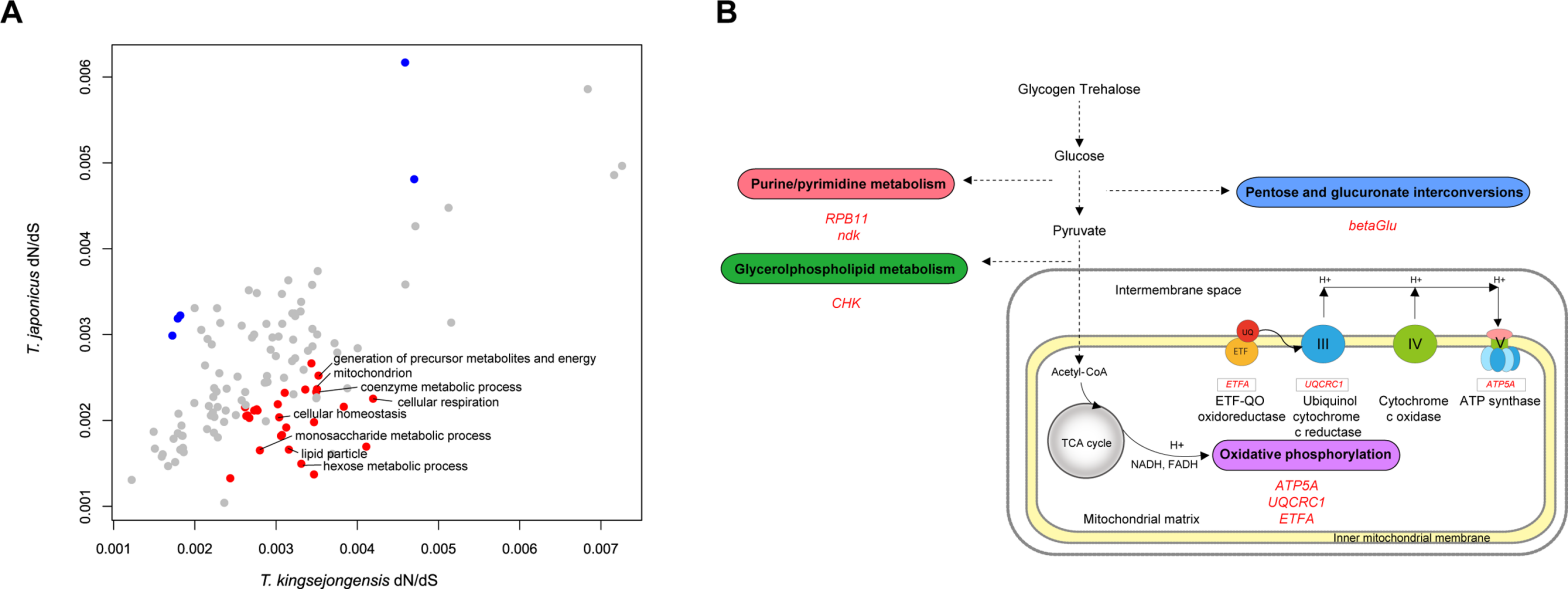
*Tigriopus kingsejongensis*-specific adaptive evolution. A. Global mean *w* (ratio of nonsynonymous (*dN*) to synonymous mutations (*dS*)) distribution by GO categories of *T. kingsejongensis* and *T. japonicus*. GO categories showing supposedly accelerated nonsynonymous divergence (binomial test, test statistic <0.05) in *T. kingsejongensis* and *T. japonicus* are colored in red and blue, respectively. B. A total of seven enzyme-coding genes were positively selected genes (PSGs) involved in the four metabolic pathways (oval frame) of *T. kingsejongensis*: energy (purple), nucleotide (red), lipid (green), and carbohydrate (blue) metabolic pathways. The three genes belonging to the oxidative phosphorylation pathway (KEGG pathway map00190) (rectangular frame) are presented below the enzymes involved. Solid lines indicate direct processes and dashed lines indicate that more than one step is involved in a process.

The average *w* value from 2937 co-orthologous genes of *T. kingsejongensis* (0.0027) is higher than that of *T. japonicus* (0.0022). GO categories that show evidence of accelerated evolution in *T. kingsejongensis* are: energy metabolism (generation of precursor metabolites and energy, GO:0006091; cellular respiration, GO:0045333) and carbohydrate metabolism (monosaccharide metabolic process, GO:0005996; hexose metabolic process, GO:0019318) (Fig. 5A, Table S10). Branch-site model analysis showed that genes belonging to these functional categories have undergone a significant positive selection process by putative functional divergence in certain lineages. There are 74 and 79 PSGs in *T. kingsejongensis* (Table S11) and *T. japonicus* (Table S12), respectively.

The functional categories enriched in *T. kingsejongensis*, when compared to *T. japonicus*, support the idea that functional divergence in *T. kingsejongensis* is strongly related to energy metabolism (oxidative phosphorylation, GO:0006119; energy-coupled proton transport down electrochemical gradient, GO:0015985; ATP synthesis-coupled proton transport, GO 0015986; generation of precursor metabolites and energy, GO:0006091) (Fig. 5B, Tables S13 and S14). In particular, three of the identified genes are involved in the oxidative phosphorylation (OxPhos) pathway, which provides the primary cellular energy source in the form of adenosine triphosphate (ATP). These three genes are nuclear-encoded mitochondrial genes: the catalytic F1 ATP synthase subunit alpha (*ATP5A*) (Fig. S4), a regulatory subunit acting as an electron transport chain such as ubiquinol-cytochrome *c* reductase core protein (*UQCRC1*) (Fig. S5), and an electron transfer flavoprotein alpha subunit (*ETFA*) (Fig. S6).

### Availability of supporting data

*T. kingsejongensis* genome and transcriptome data are deposited in the Sequence Read Archive (SRA) as BioProjects PRJNA307207 and PRJNA307513, respectively. Other supporting data is available in the *GigaScience* repository, GigaDB [53].

### Additional file

Supplementary data are available at *GIGSCI* online.

**Figure S1.** Map showing location of the *Tigriopus kingsejongensis* sampling site.

**Figure S2.** BLAST top-hit species distribution of *Tigriopus kingsejongensis*. Data obtained using BLASTx against the National Center for Biotechnology Information's (NCBI) non-redundant protein database with an E value cutoff of 1e^−5^.

**Figure S3.** Gene Ontology distribution of annotated genes. Gene Ontology (GO) annotation of predicted *Tigriopus kingsejongensis* genes was conducted using the GO annotation. The figure illustrates the number of genes from major GO modules of molecular function (MF), biological process (BP), and cellular component (CC).

**Figure S4.** *Tigriopus kingsejongensis*-specific amino acid changes in ATP synthase subunit alpha. **A**. Clustal X alignment of the amino acid sequences between four species. *Tigriopus kingsejongensis-*specific amino acid changes representing positive selections are presented with red boxes. **B**. Cartoon of the protein crystal structure of the ATP synthase (PDB ID: 1BMF). **C**. The specific amino acid change Ala166 is colored in red (in stick form) and positioned within the external loop region of nucleotide-binding domain. The three domains of the ATP synthase subunit alpha illustrated in cartoon form are colored accordingly (blue, beta-barrel domain; green, nucleotide-binding domain; purple: C terminal domain).

**Figure S5**. *Tigriopus kingsejongensis*-specific amino acid changes in ubiquinol-cytochrome c reductase core protein I. **A**. Clustal X alignment of the amino acid sequences between four species. *Tigriopus kingsejongensis*-specific amino acid changes representing positive selections are presented with red boxes. **B**. Cartoon of the protein crystal structure of ubiquinol-cytochrome c reductase (PDB ID: 1QCR). **C**. Positions of the specific amino acid changes in ubiquinol-cytochrome c reductase core protein I are colored red (stick form). The insulinase domain is yellow and the peptidase M16 domain is green.

**Figure S6**. *Tigriopus kingsejongensis*-specific amino acid changes in electron-transferring flavoprotein. **A**. Clustal X alignment of the amino acid sequences between four species. *Tigriopus kingsejongensis-*specific amino acid changes representing positive selections are presented with red boxes. Among the ten amino acid changes, the five sites are located within the N-terminal domain and the other five are positioned within the FAD binding domain. **B**. Cartoon of the protein crystal structure of the electron-transferring flavoprotein (PDB ID: 1EFV). The five amino acid sites within the FAD binding domain are colored in red (stick form). Electron-transferring flavoprotein alpha subunit is green; FAD-binding domain is represented by color-coded electrostatic surface (blue, positive charge; red, negative charge; grey, neutral charge); FAD is orange (stick form). Notably, the Asp463 residue makes a salt bridge with Arg437 in the homology model structure of electron-transferring flavoprotein from *T. kingsejongensis*. In addition, Gln454 is located near the bound FAD co-factor and may form a hydrogen bond with the N7A atom of FAD in the model structure of electron-transferring flavoprotein from *T. kingsejongensis*.

**Table S1.** Number of tRNA in the *Tigriopus kingsejongensis* genome.

**Table S2.** Known repetitive and transposable elements in the *Tigropus kingsejongensis* genome.

**Table S3.** Transposable elements in the *Tigriopus kingsejongensis* genome.

**Table S4.** Gene Ontology (GO) of lineage-specific gene families in the *Tigriopus kingsejongensis* genome. REVIGO software was used to cluster related GO terms (in bold letters) according to *P*-value.

**Table S5.** Annotated domains of lineage-specific gene families in the *Tigriopus kingsejongensis* genome.

**Table S6.** Gene Ontology (GO) of expanded gene families in the *Tigriopus kingsejongensis* genome. REVIGO software was used to cluster related GO terms (in bold letters) according to *p*-value.

**Table S7.** Gene annotation of the expanded genes in the *Tigriopus kingsejongensis* genome.

**Table S8.** Gene Ontology (GO) of contracted genes in the *Tigriopus kingsejongensis* genome. REVIGO software was used to cluster related GO terms (in bold letters) according to *P*-value.

**Table S9.** Kyoto Encyclopedia of Genes and Genomes (KEGG) pathway of expanded genes in the *Tigriopus kingsejongensis* genome.

**Table S10.** Gene Ontology (GO) categories displaying *w* (ratio of nonsynonymous (dN) to synonymous mutations (dS)) in the genomes of *Tigriopus kingsejongensis* and *T. japonicus.*

**Table S11.** Lists and annotations of positively selected genes in the *Tigriopus kingsejongensis* genome.

**Table S12.** Lists and annotations of positively selected genes in the *Tigriopus japonicus* genome.

**Table S13.** Enriched Gene Ontology (GO) categories identified by positively selected genes from the *Tigriopus kingsejongensis* genome. REVIGO software was used to cluster related GO terms (in bold letters) according to *P*-value.

**Table S14.** Enriched Gene Ontology (GO) categories identified by positively selected genes from the *Tigriopus japonicus* genome. REVIGO software was used to cluster related GO terms (in bold letters) according to *P-*value.

### List of abbreviations

ATP: Adenosine triphosphate; BUSCO: Benchmarking Universal Single-Copy Orthologs; CEGMA: Core Eukaryotic Genes Mapping Approach; CM: Covariance model; DAVID: Database for Annotation, Visualization and Integrated Discovery; *dN*: Nonsynonymous mutations; *dS*: Synonymous mutations; EST: Expressed sequence tag; GO: Gene Ontology; KEGG: Kyoto Encyclopedia of Genes and Genomes; LRT: Likelihood Ratio Test; OxPhos: Oxidative phosphorylation; PAML: Phylogenetic Analysis by Maximum Likelihood; PRANK: Probabilistic Alignment Kit; PSG: Positively selected gene; RAM: Random access memory; SRA: Sequence Read Archive; SSR: Simple sequence repeat; TE: Transposable element; *w*: *dN*/*dS* ratio

### Competing interests

The authors declare no competing interests.

### Funding

This work was supported by the Korea Polar Research Institute-funded the grant ‘Antarctic organisms: cold-adaptation mechanism and its application’ (PE16070), and basic research program (PE14260).

### Authors’ contributions

HP, S Kim and HWK conceived and designed experiments and analyses; S Kang, DHA, SGL, SCS, JL, GSM and HL performed experiments and conducted bioinformatics. Seunghyun Kang, HWK, S Kim and HP. wrote the paper. All authors read and approved the final manuscript.

## Supplementary Material

GIGA-D-16-00040_Original_Submission.pdf

GIGA-D-16-00040_Revision_1.pdf

GIGA-D-16-00040_Revision_2.pdf

GIGA-D-16-00040_Revision_3.pdf

GIGA-D-16-00040_Revision_4.pdf

Response_to_Reviewer_Comments_Original_Submission.pdf

Response_to_Reviewer_Comments_Revision_2.pdf

Response_to_Review_Comments_Revision_3.pdf

Resposne_to_Reviewer_Comments_Revision_1.pdf

Reviewer_1_Report_(Original_Submission).pdf

Reviewer_1_Report_(Revision_1).pdf

Reviewer_2_Report_(Original_Submission).pdf

Figure S1. Map showing location of the *Tigriopus kingsejongensis* sampling site.

Figure S2. BLAST top-hit species distribution of *Tigriopus kingsejongensis*. Data obtained using BLASTx against the National Center for Biotechnology Information's (NCBI) non-redundant protein database with an E value cutoff of 1e^−5^.

Figure S3. Gene Ontology distribution of annotated genes. Gene Ontology (GO) annotation of predicted *Tigriopus kingsejongensis* genes was conducted using the GO annotation. The figure illustrates the number of genes from major GO modules of molecular function (MF), biological process (BP), and cellular component (CC).

Figure S4. *Tigriopus kingsejongensis*-specific amino acid changes in ATP synthase subunit alpha. **A.** Clustal X alignment of the amino acid sequences between four species. *Tigriopus kingsejongensis-*specific amino acid changes representing positive selections are presented with red boxes. **B.** Cartoon of the protein crystal structure of the ATP synthase (PDB ID: 1BMF). **C.** The specific amino acid change Ala166 is colored in red (in stick form) and positioned within the external loop region of nucleotide-binding domain. The three domains of the ATP synthase subunit alpha illustrated in cartoon form are colored accordingly (blue, beta-barrel domain; green, nucleotide-binding domain; purple: C terminal domain).

Figure S5. *Tigriopus kingsejongensis*-specific amino acid changes in ubiquinol-cytochrome c reductase core protein I. **A.** Clustal X alignment of the amino acid sequences between four species. *Tigriopus kingsejongensis*-specific amino acid changes representing positive selections are presented with red boxes. **B.** Cartoon of the protein crystal structure of ubiquinol-cytochrome c reductase (PDB ID: 1QCR). **C.** Positions of the specific amino acid changes in ubiquinol-cytochrome c reductase core protein I are colored red (stick form). The insulinase domain is yellow and the peptidase M16 domain is green.

Figure S6. *Tigriopus kingsejongensis*-specific amino acid changes in electron-transferring flavoprotein. **A.** Clustal X alignment of the amino acid sequences between four species. *Tigriopus kingsejongensis-*specific amino acid changes representing positive selections are presented with red boxes. Among the ten amino acid changes, the five sites are located within the N-terminal domain and the other five are positioned within the FAD binding domain. **B.** Cartoon of the protein crystal structure of the electron-transferring flavoprotein (PDB ID: 1EFV). The five amino acid sites within the FAD binding domain are colored in red (stick form). Electron-transferring flavoprotein alpha subunit is green; FAD-binding domain is represented by color-coded electrostatic surface (blue, positive charge; red, negative charge; grey, neutral charge); FAD is orange (stick form). Notably, the Asp463 residue makes a salt bridge with Arg437 in the homology model structure of electron-transferring flavoprotein from *T. kingsejongensis*. In addition, Gln454 is located near the bound FAD co-factor and may form a hydrogen bond with the N7A atom of FAD in the model structure of electron-transferring flavoprotein from *T. kingsejongensis*.

Table S1. Number of tRNA in the *Tigriopus kingsejongensis* genome.

Table S2. Known repetitive and transposable elements in the *Tigropus kingsejongensis* genome.

Table S3. Transposable elements in the *Tigriopus kingsejongensis* genome.

Table S4. Gene Ontology (GO) of lineage-specific gene families in the *Tigriopus kingsejongensis* genome. REVIGO software was used to cluster related GO terms (in bold letters) according to *P*-value.

Table S5. Annotated domains of lineage-specific gene families in the *Tigriopus kingsejongensis* genome.

Table S6. Gene Ontology (GO) of expanded gene families in the *Tigriopus kingsejongensis* genome. REVIGO software was used to cluster related GO terms (in bold letters) according to *p*-value.

Table S7. Gene annotation of the expanded genes in the *Tigriopus kingsejongensis* genome.

Table S8. Gene Ontology (GO) of contracted genes in the *Tigriopus kingsejongensis* genome. REVIGO software was used to cluster related GO terms (in bold letters) according to *P*-value.

Table S9. Kyoto Encyclopedia of Genes and Genomes (KEGG) pathway of expanded genes in the *Tigriopus kingsejongensis* genome.

Table S10. Gene Ontology (GO) categories displaying *w* (ratio of nonsynonymous (dN) to synonymous mutations (dS)) in the genomes of *Tigriopus kingsejongensis* and *T. japonicus.*

Table S11. Lists and annotations of positively selected genes in the *Tigriopus kingsejongensis* genome.

Table S12. Lists and annotations of positively selected genes in the *Tigriopus japonicus* genome.

Table S13. Enriched Gene Ontology (GO) categories identified by positively selected genes from the *Tigriopus kingsejongensis* genome. REVIGO software was used to cluster related GO terms (in bold letters) according to *P*-value.

Table S14. Enriched Gene Ontology (GO) categories identified by positively selected genes from the *Tigriopus japonicus* genome. REVIGO software was used to cluster related GO terms (in bold letters) according to *P-*value.
