# Erratum to: The genome of the Antarctic-endemic copepod, *Tigriopus kingsejongensis*

**DOI:** 10.1093/gigascience/gix071

**Published:** 2017-10-06

**Authors:** Seunghyun Kang, Do-Hwan Ahn, Jun Hyuck Lee, Sung Gu Lee, Seung Chul Shin, Jungeun Lee, Gi-Sik Min, Hyoungseok Lee, Hyun-Woo Kim, Sanghee Kim, Hyun Park

**Correction**

In the final publication of “The genome of the Antarctic-endemic copepod, *Tigriopus kingsejongensi*,” by Seunghyun Kang et. al., the listed accepted date was incorrect, and the article did not include the revised date. The accepted date has been corrected and the revised date added. The corrected manuscript can be found online at https://academic.oup.com/gigascience/article/6/1/1/2865210/The-genome-of-the-Antarctic-endemic-copepod?searchresult=1.

The Publisher regrets these errors.

