# Supplementary material for: The genome of the Antarctic-endemic copepod, Tigriopus kingsejongensis
Source: Gigascience. 2017 Jan 7;6(1):1–9. doi: 10.1093/gigascience/giw010 (PMC5467011; doi:10.1093/gigascience/giw010)
Supplement: GIGA-D-16-00040_Original_Submission.pdf [file giw010_GIGA-D-16-00040_Original_Submission.pdf]

Manuscript

Click here to view linked References

Click here to download Manuscript 20160627_Tigriopus_ms_referece formatted.docx

# The First Copepod Genome Reveals Evolutionary Adaptation to Extreme Environments in the Antartic-endemic *Tigriopus*

Seunghyun Kang[1¶], Do-Hwan Ahn[1¶], Jun Hyuck Lee[1,2], Sung Gu Lee[1,2], Seung Chul Shin[1], Jungeun Lee[1], Gi-Sik Min[3], Hyoungseok Lee[1], Hyun-Woo Kim[4*&], Sanghee Kim[1*&] & Hyun Park[1,2*&]

[1] Division of Polar Life Sciences, Korea Polar Research Institute, Yeonsu-gu, Incheon, South Korea

[2] Polar Sciences, University of Science & Technology, Yuseong-gu, Daejeon, South Korea

[3] Department of Biological Sciences, Inha University, Incheon, South Korea

[4] Department of Marine Biology, Pukyong National University, Busan, South Korea

* Corresponding author

E-mail: kimhw@pknu.ac.kr; sangheekim@kopri.re.kr; hpark@kopri.re.kr

[¶]These authors contributed equally to this work.

[&]These authors also contributed equally to this work.

## Abstract

**Background:** The subclass copepods are more rich in species than any other group of multicellular animals including insects and nematodes. Despite of their extraordinary economic and ecological importance, still genomic resources are limited to these groups. The Antarctic intertidal zone is continuously subject to extreme fluctuations in biotic and abiotic stressors, and the West Antarctic Peninsula is the most rapidly warming region on earth. Organisms living in Antarctic intertidal pools are therefore of great interest for research on topics such as evolutionary adaptation to extreme environments and the effects of climate change.

**Results:** Here, we report the whole-genome sequence of the endemic Harpacticoid copepod, *Tigriopus kingsejongensis*. Comparative genome analysis revealed that *T. kingsejongensis* specific genes are enriched in transport and metabolism processes. Furthermore, rapidly evolving genes related to energy metabolism showed signatures of positive selection. Evolutionary adaptation to cold temperatures has led to the distinct feature that transmembrane transport genes (*TkTre*t) in functional categories are highly induced at low temperatures. The *TkTret* gene family is regulated at low temperatures by specific transcription factors, these have been reported to be involved in the transport of trehalose, which provides a cryo- or anhydroprotectant nutrient source that helps protect against environmental stresses. Interestingly, this phenomenon is not observed in the temperate genome of *Tigriopus* specie.

**Conclusions:** The genome of *T. kingsejongensis* therefore provides an interesting example of an evolutionary strategy for Antarctic cold adaptation, and offers new genetic insights into Antarctic intertidal biota.



## Keywords

Copepoda, Genomics, Antarctic, adaptation, *Tigriopus*

## Background

The subclass copepods are more diverse than any other group of multicellular animals including insects and nematodes and so far approximately 12,000 described copepod species have been described [1, 2]. They dominate the zooplankton community contributing about 70% of total zooplankton biomass [3] and play an important role in the marine meiobenthic food web linking between the phytoplankton and higher trophic levels [4]. Harpacticoid copepods of the genus *Tigriopus* Norman 1868 are dominant members of shallow supratidal rock pools worldwide. They are distributed among habitats that vary widely in salinity, temperature, desiccation risk, and UV radiation, and have been used as a model system to investigate topics such as osmoregulation [5], temperature adaptation [6, 7] and environmental toxicology [8]. Although their biological and economic importance, currently there are no published Copepoda genome available yet.

Antarctica provides not only an extreme habitat for extant organisms, but also a model for research on evolutionary adaptations to cold environments [9, 10]. The Antarctic intertidal zone, particularly in the Western Antarctic Peninsula region, is one of the most extreme environments on earth. It also serves as a potential barometer for global climate changes, since it is the fastest-warming region on earth [11]. Antarctic intertidal species that have evolved stenothermal phenotypes through adaptation to a year-round climate of extreme cold may now face extinction by global warming. The response of these species to further warming in Western Antarctica is of serious concern; however, to date there have been few studies focusing on species from the Antarctic intertidal zone.

*Tigriopus kingsejongensis* was first found and recognized as a new endemic species in a rock pool in the Antarctic Peninsula (Figure S1 and S2 in Additional file 1), and is extremely cold-tolerant and can survive in frozen sea water [12]. We observed the morphological differences,

such as increased numbers of caudal setae in nauplii, optimal growth temperature (ca. 8°C) and developmental characteristics have been compared to those of the congener *Tigriopus japonicus,* which is found in the coastal area of the Yellow Sea. *Tigriopus kingsejongensis* has evolved to overcome the unique environmental constrains of Antarctica, and therefore provides an ideal experimental model for all aspects of research on extreme habitats. This species may represent a case of rapid speciation, since the intertidal zone on King George Island and surrounding areas did not exist before 10,000 years ago [13]. *Tigriopus kingsejongensis* likely evolved as a distinct species within this relatively short time period. Thus, inter- and intraspecies comparative analyses of Antarctic *Tigriopus* species will help define the trajectory of adaptation to the Antarctic environment and also provide insights into the genetic basis of *Tigriopus* divergence and evolution.

## Data description

In this study, we sequenced the genome of *T. kingsejongensis* using a whole-genome shotgun strategy with the Illumina Miseq platform. *De novo* assembly of 203 million reads from paired-end libraries and mate-paired libraries yielded a draft assembly (65-fold coverage) with a total length of 295 Mb, and contig and scaffold N50 sizes of 17.6 kb and 159.2 kb, respectively. Non-gap sequences occupied 284.8 Mb (96.5%), and simple sequence repeats (SSRs) were 1.2 Mb (0.4%) in total. Transposable elements (TEs) comprised 6.5 Mb, which is roughly 2.3% of the assembled genome (Figure S3 and S4 in Additional file 1, Table S1-S5 in Additional file 2). On the basis of homology and *ab initio* gene prediction, we found that the genome of *T. kingsejongensis* contains 12,772 protein-coding genes (Figure S5 and S6 in Additional file 1, Table S6 and S7 in Additional file 2). By assessing the quality of the annotated 12,772 gene models, we found that 11,686 protein-coding genes (91.5%) were supported by the RNA-seq data, of which, 7,325 (63%) showed similarity to proteins from

other species. We also found that 376 of the 458 CEGMA (Core Eukaryotic Genes Mapping Approach) core genes were identified in the gene models, of which, 356 (94.7%) were supported by the RNA-seq data.

## Analysis

### Gene families

We constructed orthologous gene clusters using four arthropod species (Antarctic copepod, *T. kingsejongensis*; scorpion, *Mesobuthus martensii*; fruit fly, *Drosophila melanogaster* and water flea, *Daphnia pulex*) to compare the genomic features and the adaptive divergence in the arthropods. In total, 2,063 gene families are shared by all four species, and 1,028 genes are specific to the Antarctic copepod. *Tigriopus kingsejongensis* shares 4,559 (73.5%) gene families with *D. pulex,* which belongs to the same crustacean lineage Vericrustacea, 3,531 (56.9%) with *D. melanogaster,* and 3,231 (52.1%) with *M. martensii* (Figure 1A). Gene ontology (GO) analysis revealed that the 1,028 *T. kingsejongensis*-specific genes are enriched in transport (single-organism transport, GO: 0044765; transmembrane transport, GO: 0055085; ion transport, GO: 0006811; cation transport, GO: 0006812) and single-organism metabolic processes (GO: 0044710) (Table S8 and S9). Subsequently, we performed gene gain-and-loss analysis on 11 representative species, and found that *T. kingsejongensis* gained 735 gene families and lost 4,401 gene families (Figure 1B, Table S10 in Additional file 2). Thus, this species exhibits a gene family turnover of 5,136, the largest value among the eight arthropods. We also analyzed expansion and contraction of the gene families (Table S11-S13), and found 232 significantly expanded gene families in *T. kingsejongensis*; these gene families are significantly overrepresented in amino acid metabolism and carbohydrate metabolism in KEGG metabolic pathways (Table S14 in

Additional file 2).

## Genome evolution

Adaptive functional divergence caused by natural selection is commonly estimated based on the ratio of nonsynonymous (*dN*) to synonymous (*dS*) mutations. The average *dN*/*dS* ratio (*w*) from 2,937 co-orthologous genes of *T. kingsejongensis* (0.0027) is higher than that of *T. japonicus* (0.0022). The GO categories that showing evidence of accelerated evolution in *T. kingsejongensis* are energy metabolism (generation of precursor metabolites and energy, GO: 0006091; cellular respiration, GO: 0045333) and carbohydrate metabolism (monosaccharide metabolic process, GO: 0005996; hexose metabolic process, GO: 0019318) (Figure 2A, Table S15 in Additional file 2). Branch-site model analysis showed that the genes belonging to the functional categories above have undergone a significant positive selection process by putative functional divergence in certain lineages. There are 74 and 79 positively selected genes (PSGs) in *T. kingsejongensis* (Table S16 in Additional file 2) and *T. japonicus* (Table S17 in Additional file 2), respectively. The functional categories enriched in *T. kingsejongensis*, when compared to *T. japonicus*, support the idea that the functional divergence in *T. kingsejongensis* is strongly related to energy metabolism (oxidative phosphorylation, GO: 0006119; energy-coupled proton transport down electrochemical gradient, GO: 0015985; ATP synthesis-coupled proton transport, GO: 0015986; generation of precursor metabolites and energy, GO: 0006091) (Figure 2B, Table S18 and S19 in Additional file 2). In particular, three of the identified genes are involved in the oxidative phosphorylation (OxPhos) pathway, which provides the primary cellular energy source in the form of adenosine triphosphate (ATP). These three genes are nuclear-encoded mitochondrial genes: the catalytic F1 ATP synthase subunit alpha (*ATP5A*) (S7 Fig), a regulatory subunit acting as an electron transport chain such as ubiquinol-cytochrome *c* reductase core protein (*UQCRC1*) (S8 Fig), and an electron transfer flavoprotein

alpha subunit (*ETFA*) (S9 Fig).

## Temperature-specific gene expression patterns

Cold temperatures are a key factor in the Antarctic environment, and represent a major driving force of adaptation and evolution among Antarctic organisms. To determine the genetic basis for the metabolic adjustment of *Tigriopus* to cold and warm temperatures, we compared temperature-dependent RNA expression at 4°C and 15°C (Figure 3A, Table S6 in Additional file 2). Approximately, the temperature specific libraries yielded 15-16 million reads per experiment with about 3.5-3.8 billion bases each. Among 12,772 annotated genes, 2,276 genes are over-expressed at 4°C, whereas 2,560 genes are over-expressed at 15°C. Twenty-three GO terms are enriched based on a GO enrichment test for genes showing greater than 2-fold overexpression at 4°C (S10 Fig, Table S20 in Additional file 2). Among these GO terms, the transmembrane transport (GO: 0055085) term included four facilitated trehalose transporter genes (*Tret*) (Table S21 in Additional file 2). Trehalose is the main hemolymph sugar that serves as a nutrient source in most arthropods; further, it acts as a protectant, promoting insect survival against harsh conditions such as desiccation, heat and cold [14, 15]. One strategy used by many insects to tolerate cold temperatures is the induction of high levels of polyols (such as glycerol or sugars including trehalose), which act as cryoprotectants. In Antarctic midge, the injection of trehalose enhanced resistance to heat and cold stress and dehydration [16]. The *T. kingsejongensis* genome includes seven *Tret* genes (Figure S11 and S12 in Additional file 1), which is higher than other arthropods (*D. melanogaster* and *T. japonicus* possess four *Tret* genes each) (Table S22 in Additional file 2).

A duplication event of common gene families, reported in the genome of *D. pulex* [17-20], might be related to evolutionary innovations such as neofunctionalization and

subfunctionalization [17]. Duplication of *Tret* genes in Antarctic *Tigriopus* (*TkTret*) may represent an important evolutionary innovation, since they are known to be involved in the production of cryoprotectant nutrients. Notably, at cold temperatures, the expression of two *Tret* genes (*TkTret1-3* and *TkTret1-7*) were highly upregulated (38 to 42-fold), and those of two other *Tret* genes (*TkTret1-5* and *TkTret1-6*) were slightly upregulated (2 to 5-fold) in the *T. kingsejongensis*. Additionally, we found 356 overrepresented transcription factor binding sites in the promoter regions among 2,276 upregulated genes at 4°C using F-Match analysis with TRANSFAC data (Table S23 in Additional file 2) [21]. Four upregulated *TkTret* genes contain 16 kinds of transcription factor binding regions (Figure 3B and C), among which, 12 are shared by all upregulated genes. Transcription factor gene expression patterns for the *TkTret* genes showed that the expressions of *ELK1*, *OCT1*, *PAX4*, *BRN2* and *MCM1* are increased at low temperatures compared to those at high temperatures (Figure 3D). *ELK1 and OCT1* are known to play important roles in the developmental process [22, 23], and *MCM1* has been speculated to coordinate cell cycle progression with changes in cell wall integrity and metabolic activity [24].

## Discussion

Although it is difficult to elucidate the transcription factor's primary role in the cell, differential expression of transcription factors is an important mechanism for regulating the expression of targeted genes in cold environments. These putative cold-regulated transcription factors have been reported to regulate gene networks not only to maintain normal energy production in cold environments, but also to help withstand constant low temperatures and seasonal food scarcity in the extreme environment of Antarctica. We were unable to see the increase on genes responsible for trehalose biosynthesis, assuming that *T. kingsejongensis* regulate the trehalose levels via facilitation of transporter in harsh but endurable condition (e.g.,

4°C in our experiment) rather than synthesize trehalose itself which usually happens in life-threatening condition (e.g., desiccation and frozen). As in larvae of *Polypedilum vanderplanki,* desiccation stress induces trehalose synthesis [25, 26] and gene expression of *Tret1* in the fat body, it would be interesting to see whether the similar induction happens during some days on a year-round condition in Antarctica.

Mitochondrial DNA- and nuclear-encoded mitochondrial genes, particularly those belonging to the OxPhos pathway, are highly conserved, even between distantly related species [27]. Mutations in mitochondrial genes are known to cause a variety of negative effects, including increased oxidative stress, a reduction in body mass and survival, and metabolic disorders [28-30]. Polymorphism within *ATP5A* in ovenbirds is associated with higher individual fitness by conferring increased body mass [31], implying a possible role for this gene in environmental adaptation. Furthermore, *UQCRC1* polymorphism in humans is associated with body lipid accumulation [32], and *ETFA* polymorphism is related to altered thermal stability of enzyme activity [33, 34]. The most distinct environmental adaptations for Antarctic *Tigriopus* are to a constant low temperature and seasonal food scarcity in Antarctica. The genetic variations on genes can answer for the successful settlement of *T. kingsejongensis,* by fulfilling the need for efficient energy production and adequate energy preservation in an extremely cold environment.

Despite their extraordinary species richness, economic and environmental importance, genomic and genomic information about copepods has been limited. This study presents the first genome sequence, to our knowledge, of Copepoda. The evolutionary analyses based on novel *T. kingsejongensis* whole-genome and transcriptome data provide important insights into adaptation to harsh Antarctic environments. Further understanding of the signatures of adaptive evolution in similar environments is necessary for functional analysis of the genes identified in

this species. The present work provides an Antarctic eco-model system that can be used for further research on adaptations, ecological and population studies of Antarctic biota, as well as a foundation for addressing key issues related to the management of Antarctic environmental changes.

## Methods

### DNA library construction and sequencing

*Tigriopus kingsejongensis* were collected from tidal pools in Potter Cove, near King Sejong Station, on the northern Antarctic Peninsula (62°14'S, 58°47'W) in January 2013 with a hand-nets (Figure S1 and S2 in Additional file 1). Water temperatures were 1.6 ± 0.8°C during this month. High-molecular-weight genomic DNA from pooled *T. kingsejongensis* was extracted using the DNeasy Blood & Tissue Kit (Qiagen). For Illumina Miseq sequencing, four library types were constructed with 350, 400, 450, and 500 bp for paired-end libraries, and 3 kb and 8 kb for mate-pair libraries, prepared using the standard Illumina sample preparation methods (Table S1 in Additional file 2). All sequencing processes were performed according to the manufacturer's instructions (Illumina).

### Genome assembly

First, assemblies were performed using a Celera Assembler with Illumina short reads [35. Prior to assembly, Illumina reads were trimmed using the FASTX-Toolkit (http://hannonlab.cshl.edu/fastx_toolkit) with parameters -t 20, -l 70 and -Q 33, after which a paired sequence from trimmed Illumina reads was selected. Finally, trimmed Illumina reads with 65-fold coverage (insert sizes 350, 400, 450, and 500 bp) were obtained and converted to the FRG file format (required by the Celera assembler) using FastqToCA. Assembly was

performed on a 96-processor workstation with Intel Xeon X7460 2.66 GHz processors and 1 terabyte RAM with the following parameters: overlapper = ovl, unitigger = bogart, utgGraphErrorRate = 0.03, utgGraphErrorLimit = 2.5, utgMergeErrorRate = 0.030, utgMergeErrorLimit = 3.25, ovlErrorRate = 0.1, cnsErrorRate = 0.1, cgwErrorRate = 0.1, merSize = 22, and doOverlapBasedTrimming = 1. The initial Celera assembly had a total size of 305 Mb, N50 contig size of 17,566 bp, and max contig size of 349.5 kb. Scaffolding was completed using the software SSPACE 2.0 scaffolder using mate-paired data {Boetzer, 2011 #2344]. Subsequently, we closed gaps using Gapfiller Ver.1.9 software with 65× trimmed Illumina reads with default settings [36]. The final result included a total of 11,558 scaffolds (295 Gb in length) with 10 Mb gaps having an N50 length of 159 kb and a max length of 3.4 Mb.

**Annotation**

We used MAKER for genome annotation [37]. MAKER is a portable and easily configurable genome annotation pipeline. MAKER first identified repetitive elements using RepeatMasker [38]. This masked genome sequence was used for *ab initio* gene prediction with SNAP software [39], after which alignment of expressed sequence tags with BLASTn and protein information from tBLASTx were included. We used the *de novo* repeat library of *T. kingsejongensis* from RepeatModeler for RepeatMasker; proteins from five species with data from *D. melanogaster*, *D. pulex*, *T. japonicus*, and *Tigriopus californicus* were included in the analysis. RNA-seq-based gene prediction was performed by aligning all RNA-seq data against the assembled genome using TopHat [40], and Cufflinks [41] was used to predict cDNAs from the resultant data. Next, MAKER polished the alignments using the program Exonerate [42], which provided integrated information to synthesize SNAP annotation. MAKER then selected and revised the final gene model considering all information. A total of 12,772 genes were

predicted using MAKER in *T. kingsejongensis* (Table S3-S7 in Additional file 2). Annotated genes contained an average of 4.6 exons, with an average mRNA length of 1,090 bp. Additionally, 12,562 of 12,772 genes were assigned preliminary functions based on automated annotation using Blast2GO (Ver. 2.6.0) [43] (Figure S3 and S4 in Additional file 1). The Infernal software package (Ver. 1.1) [44] and covariance models (CMs) from the Rfam database [45] were used to identify other non-coding RNAs in the *T. kingsejongensis* scaffold. We identified putative tRNA genes using tRNAscan-SE [46]. tRNAscan-SE uses a covariance model (CM) that scores candidates based on their sequence and predicted secondary structures.

**Gene families**

The orthologous groups were identified from 11 species (*T. kingsejongensis, Aedes aegypti*, *D. melanogaster*, *Ixodes scapularis, M. martensii*, *Strigamia martima*, *Tetranychus urticae, D. pulex, Homo sapiens, Ciona intestinalis,* and *Caenorhabditis elegans*) (Table S10 in Additional file 2) using OrthoMCL [47] with standard parameters and options, and transcript variants other than the longest translation forms were removed. For *T. kingsejongensis*, the coding sequence from the MAKER annotation pipeline was used. The 1:1:1 single-copy orthologous genes were subjected to phylogenetic construction and divergence time estimation. Protein-coding genes were aligned using PRANK with the codon alignment option [48], and poorly aligned sequences with gaps were removed using Gblock under the codon model [49]. We constructed a maximum-likelihood phylogenetic tree using RAxML with 1,000 bootstrap values [50] and calibrated the divergence time between species with TimeTree [51]. Finally, the average gene gain/loss rate along the given phylogeny was identified using the program CAFÉ 3.1 [52].

**Evolutionary analysis**

To estimate *dN*, *dS*, *w*, and lineage-specific PSGs in *T. kingsejongensis* and *T. japonicus*, protein-coding genes from *T. japonicus* (Table S24 in Additional file 2) were added to define orthologous gene families among the four species (*T. kingsejongensis, T. japonicus, D. pulex*, and *D. melanogaster*) using the program OrthoMCL with the same conditions previously described. We identified 2,937 orthologous groups shared by all four species, and single-copy gene families were used to construct a phylogenetic tree and estimate the time since divergence using the same methods described above. Each of the identified orthologous genes was aligned using the PRANK, and poorly aligned sequences with gaps were removed using Gblock. Alignments showing less than 40% identity and genes shorter than 150 bp were eliminated in subsequent procedures. The values of *dN*, *dS* and *w* were estimated from each gene using the Codeml program implemented in the PAML package with the free-ratio model [53] under F3X4 codon frequencies, and orthologs with $w \leq 5$ and $dS \leq 3$ were retained [54]. To examine the accelerated nonsynonymous divergence in either *T. kingsejongensis* or *T. japonicus* lineage, a binomial test [55] was used to determine GO categories with at least 20 orthologous genes. To define PSGs in *T. kingsejongensis* and *T. japonicus*, we applied basic and branch-site models, and Likelihood Ratio Tests (LRTs) were used to remove genes under relaxation of selective pressure. To investigate which functional categories and pathways were enriched in the PSGs, we performed DAVID Functional Annotation [56] with Fisher's exact test (cutoff: $P \leq 0.05$).

**Gene expression under temperature stress**

*Tigriopus kingsejongensis* were captured at the King Sejong Station (62°14'S, 58°47'W) and acclimated in large tanks with circulating fresh sea water at 4.0 ± 0.2°C for at least 7 days prior to experiments. We prepared two other large tanks at 4°C and 15°C for control

and heat stress, respectively. After acclimation, two groups of 20 specimens each of *T. kingsejongensis* were collected at 5 days after culture. For RNA-seq experiments, we prepared mRNA from each sample. Sequencing was performed with Illumina Miseq, and generated reads were trimmed to ~30 bases in length and ~20 in base quality (Table S6 in Additional file 2). Trimmed reads of each tissue were mapped to the annotated scaffold of the *T. kingsejongensis* genome. Fragments per kilobase of exon per million fragments mapped (FPKM) values and fold changes in expression were calculated for each gene in each sample with a significance threshold of $P \leq 0.05$ using CLC Genomics Workbench (Ver. 8.0).

**RNA-seq analysis of *T. japonicus***

*Tigriopus japonicus* experiments were carried out under the same conditions as described above. The *de novo* transcriptome assembly was performed with CLC Genomics Workbench, setting the minimum allowed contig length to 200 nucleotides. The assembly process generated 40,172 contigs with a max length of 23,942 bp and an N50 value of 1,093 bp. These generated contigs were used as reference sequences for mapping of trimmed reads, and fold changes in expression for each gene were calculated with a significance threshold of $P \leq 0.05$ using CLC Genomics Workbench (Table S24 in Additional file 2).

## Availability of supporting data

The data for *T. kingsejongensis* genome and transcriptome has been deposited in the SRA as BioProject PRJNA307207 and PRJNA307513, respectively.

## List of abbreviations

facilitated trehalose transporter genes, *Tret*; *Tret* genes in Antarctic *Tigriopus*, *TkTret*; and simple sequence repeats, SSRs; Transposable elements, TEs; CEGMA, Core Eukaryotic Genes Mapping Approach; Gene ontology, GO; nonsynonymous mutations, *dN*; synonymous mutations, *dS*; average *dN/dS* ratio, *w*; positively selected genes, PSGs; oxidative phosphorylation, OxPhos; adenosine triphosphate, ATP; catalytic F1 ATP synthase subunit alpha, *ATP5A*; ubiquinol-cytochrome c reductase core protein, *UQCRC1*; electron transfer flavoprotein alpha subunit, *ETFA*; covariance models, CMs; Likelihood Ratio Tests, LRTs; Fragments per kilobase of exon per million fragments mappedvalues, FPKM

## Competing interests

The authors declare no competing interests.

## Funding

This work was supported by an Antarctic organisms: Cold-adaptation mechanism and its application grant (PE16070) and the basic research program (PE14260) funded by the Korea Polar Research Institute (KOPRI).

## Author contributions

H.P., Sanghee Kim and H.W.K. conceived and designed experiments and analyses; Seunghyun Kang, D.-H.A., S.G.L., S.C.S., J.L., G.S.M. and H.L. performed experiments and conducted bioinformatics. Seunghyun Kang, H.W.K., Sanghee Kim and H.P. wrote the paper.

## Acknowledgements

We would like to thank Joseph A. Covi for comments and discussion.

## References


1. Huys R, Boxshall GA: *Copepod evolution.* Ray Society; 1991.

2. Humes AG: **How many copepods?** *Hydrobiologia* 1994, **292:**1-7.

3. Wells P, Persoone G, Jaspers E, C. C: *Marine ecotoxicological tests with zooplankton. In: Persoone, G., Jaspers, E., Claus, C. (Eds.), Ecotoxicological Testing for the Marine Environment.* Inst. Mar. Sci. Res., Bredene; 1984.

4. Ruppert E, Fox R, Barnes R: **Invertebrate Zoology, A Functional Evolutionary Approach. Brooks/Cole-Thomson Learning.** *Belmont, CA* 2003.

5. Goolish E, Burton R: **Energetics of osmoregulation in an intertidal copepod: Effects of anoxia and lipid reserves on the pattern of free amino accumulation.** *Funct Ecol* 1989**:**81-89.

6. Lazzaretto I, Libertini A: **Karyological comparison among different Mediterranean populations of the genus *Tigriopus* (Copepoda Harpacticoida).** *Boll Zool* 2009, **53:**197-201.

7. Davenport J, Barnett P, McAllen R: **Environmental tolerances of three species of the harpacticoid copepod genus *Tigriopus*.** *J Mar Biol Assoc UK* 1997, **77:**3-16.

8. Raisuddin S, Kwok KW, Leung KM, Schlenk D, Lee J-S: **The copepod *Tigriopus*: A promising marine model organism for ecotoxicology and environmental genomics.** *Aquat Toxicol* 2007, **83:**161-173.

9. Thorne MAS, Kagoshima H, Clark MS, Marshall CJ, Wharton DA: **Molecular analysis of the cold tolerant Antarctic Nematode, *Panagrolaimus davidi*.** *PLOS one* 2014, **9:**e104526.

10. Everatta MJ, Worlandb MR, Balea JS, Conveyb P, Hayward SAL: **Pre-adapted to the maritime Antarctic? – Rapid cold hardening of the midge, *Eretmoptera murphyi*.**

*J Insect Physiol* 2012, **58:**1104–1111.

11. Bromwich DH, Nicolas JP, Monaghan AJ, Lazzara MA, Keller LM, Weidner GA, Wilson AB: **Central West Antarctica among the most rapidly warming regions on Earth.** *Nature Geoscience* 2013, **6:**139-145.

12. Park E-O, Lee S, Cho M, Yoon SH, Lee Y, Lee W: **A new species of the genus *Tigriopus* (Copepoda: Harpacticoida: Harpacticidae) from Antarctica.** *Proc Biol Soc Wash* 2014, **127:**138-154.

13. Birkenmajer K: **Geology of Admiralty Bay, King George Island (South Shetland Islands). An outline.** *Pol Polar Res* 1980, **1:**29-54.

14. Crowe JH, Carpenter JF, Crowe LM: **The role of vitrification in anhydrobiosis.** *Annu Rev Physiol* 1998, **60:**73-103.

15. Arrese EL, Soulages JL: **Insect fat body: energy, metabolism, and regulation.** *Annu Rev Entomol* 2010, **55:**207.

16. Benoit JB, Lopez-Martinez G, Elnitsky MA, Lee RE, Denlinger DL: **Dehydration-induced cross tolerance of *Belgica antarctica* larvae to cold and heat is facilitated by trehalose accumulation.** *Comparative Biochemistry and Physiology Part A: Molecular & Integrative Physiology* 2009, **152:**518-523.

17. Boucher P, Ditlecadet D, Dubé C, Dufresne F: **Unusual duplication of the insulin-like receptor in the crustacean *Daphnia pulex*.** *BMC Evol Biol* 2010, **10:**305.

18. Baldwin WS, Marko PB, Nelson DR: **The cytochrome P450 (*CYP*) gene superfamily in *Daphnia pulex*.** *BMC Genomics* 2009, **10:**169.

19. Zeis B, Lamkemeyer T, Paul RJ, Nunes F, Schwerin S, Koch M, Schütz W, Madlung J, Fladerer C, Pirow R: **Acclimatory responses of the *Daphnia pulex* proteome to environmental changes. I. Chronic exposure to hypoxia affects the oxygen transport system and carbohydrate metabolism.** *BMC Physiol* 2009, **9:**7.

20. Sturm A, Cunningham P, Dean M: **The ABC transporter gene family of *Daphnia pulex*.** *BMC Genomics* 2009, **10:**170.

21. Matys V, Kel-Margoulis OV, Fricke E, Liebich I, Land S, Barre-Dirrie A, Reuter I, Chekmenev D, Krull M, Hornischer K: **TRANSFAC® and its module TRANSCompel®: transcriptional gene regulation in eukaryotes.** *Nucleic Acids Res* 2006, **34:**D108-D110.

22. Tiensuu T, Larsen MK, Vernersson E, Tuck S: ***lin-1* has both positive and negative functions in specifying multiple cell fates induced by Ras/MAP kinase signaling in *C. elegans*.** *Dev Biol* 2005, **286:**338-351.

23. Ryan AK, Rosenfeld MG: **POU domain family values: flexibility, partnerships, and developmental codes.** *Genes Dev* 1997, **11:**1207-1225.

24. Kuo M-H, Grayhack E: **A library of yeast genomic MCM1 binding sites contains genes involved in cell cycle control, cell wall and membrane structure, and metabolism.** *Mol Cell Biol* 1994, **14:**348-359.

25. Kikawada T, Minakawa N, Watanabe M, Okuda T: **Factors inducing successful anhydrobiosis in the African chironomid *Polypedilum vanderplanki*: significance of the larval tubular nest.** *Integr Comp Biol* 2005, **45:**710-714.

26. Kikawada T, Saito A, Kanamori Y, Nakahara Y, Iwata K-i, Tanaka D, Watanabe M, Okuda T: **Trehalose transporter 1, a facilitated and high-capacity trehalose transporter, allows exogenous trehalose uptake into cells.** *Proc Natl Acad Sci USA* 2007, **104:**11585-11590.

27. Martinez-Cruz O, Muhlia-Almazan A, Sanchez-Paz A, Garcia-Carreño F, Jimenez-Gutierrez L, Toro MdlANd: *Invertebrates mitochondrial function and energetic challenges.* INTECH Open Access Publisher; 2012.

28. Schapira AH: **Mitochondrial diseases.** *The Lancet* 2012, **379:**1825-1834.

29. Tuppen HA, Blakely EL, Turnbull DM, Taylor RW: **Mitochondrial DNA mutations and human disease.** *Biochimica et Biophysica Acta (BBA)-Bioenergetics* 2010, **1797:**113-128.

30. Johannsen DL, Ravussin E: **The role of mitochondria in health and disease.** *Curr Opin Pharm* 2009, **9:**780-786.

31. Toms JD, Eggert LS, Arendt WJ, Faaborg J: **A genetic polymorphism in the sex-linked *ATP5A1* gene is associated with individual fitness in Ovenbirds (*Seiurus aurocapilla*).** *Ecology and evolution* 2012, **2:**1312-1318.

32. Kunej T, Wang Z, Michal JJ, Daniels TF, Magnuson NS, Jiang Z: **Functional *UQCRC1* polymorphisms affect promoter activity and body lipid accumulation.** *Obesity* 2007, **15:**2896-2901.

33. Henriques BJ, Fisher MT, Bross P, Gomes CM: **A polymorphic position in electron transfer flavoprotein modulates kinetic stability as evidenced by thermal stress.** *FEBS Lett* 2011, **585:**505-510.

34. Bross P, Pedersen P, Winter V, Nyholm M, Johansen BN, Olsen RKJ, Corydon MJ, Andresen BS, Eiberg H, Kølvraa S, Gregersen N: **A polymorphic variant in the human electron transfer flavoprotein α-chain (α-T171) displays decreased thermal stability and is overrepresented in very-long-chain acyl-coA dehydrogenase-deficient patients with mild childhood presentation.** *Mol Genet Metab* 1999, **67:**138-147.

35. Myers EW, Sutton GG, Delcher AL, Dew IM, Fasulo DP, Flanigan MJ, Kravitz SA, Mobarry CM, Reinert KH, Remington KA, et al: **A whole-genome assembly of *Drosophila*.** *Science* 2000, **287:**2196-2204.

36. Nadalin F, Vezzi F, Policriti A: **GapFiller: a *de novo* assembly approach to fill the gap within paired reads.** *BMC Bioinformatics* 2012, **13:**S8.

37. Holt C, Yandell M: **MAKER2: an annotation pipeline and genome-database management tool for second-generation genome projects.** *BMC Bioinformatics* 2011, **12:**491.

38. Smit AFA HR, Green, P.: **RepeatMasker Open-3.0. 1996-2004 (http://www.RepeatMakser.org).**

39. Korf I: **Gene finding in novel genomes.** *BMC Bioinformatics* 2004, **5:**59.

40. Trapnell C, Pachter L, Salzberg SL: **TopHat: discovering splice junctions with RNA-Seq.** *Bioinformatics* 2009, **25:**1105-1111.

41. Trapnell C, Williams BA, Pertea G, Mortazavi A, Kwan G, van Baren MJ, Salzberg SL, Wold BJ, Pachter L: **Transcript assembly and quantification by RNA-Seq reveals unannotated transcripts and isoform switching during cell differentiation.** *Nat Biotech* 2010, **28:**511-515.

42. Slater GS, Birney E: **Automated generation of heuristics for biological sequence comparison.** *BMC Bioinformatics* 2005, **6:**31.

43. Conesa A, Gotz S, Garcia-Gomez JM, Terol J, Talon M, Robles M: **Blast2GO: a universal tool for annotation, visualization and analysis in functional genomics research.** *Bioinformatics* 2005, **21:**3674-3676.

44. Nawrocki EP, Kolbe DL, Eddy SR: **Infernal 1.0: inference of RNA alignments.** *Bioinformatics* 2009, **25:**1335-1337.

45. Gardner PP, Daub J, Tate J, Moore BL, Osuch IH, Griffiths-Jones S, Finn RD, Nawrocki EP, Kolbe DL, Eddy SR, Bateman A: **Rfam: Wikipedia, clans and the "decimal" release.** *Nucleic Acids Res* 2011, **39:**D141-145.

46. Lowe TM, Eddy SR: **tRNAscan-SE: a program for improved detection of transfer RNA genes in genomic sequence.** *Nucleic Acids Res* 1997, **25:**955-964.

47. Li L, Stoeckert CJ, Roos DS: **OrthoMCL: identification of ortholog groups for**

**eukaryotic genomes.** *Genome Res* 2003, **13:**2178-2189.

48. Löytynoja A, Goldman N: **An algorithm for progressive multiple alignment of sequences with insertions.** *Proc Natl Acad Sci U S A* 2005, **102:**10557-10562.

49. Castresana J: **Selection of conserved blocks from multiple alignments for their use in phylogenetic analysis.** *Mol Biol Evol* 2000, **17:**540-552.

50. Stamatakis A: **RAxML version 8: a tool for phylogenetic analysis and post-analysis of large phylogenies.** *Bioinformatics* 2014, **30:**1312-1313.

51. Hedges SB, Dudley J, Kumar S: **TimeTree: a public knowledge-base of divergence times among organisms.** *Bioinformatics* 2006, **22:**2971-2972.

52. Han MV, Thomas GW, Lugo-Martinez J, Hahn MW: **Estimating gene gain and loss rates in the presence of error in genome assembly and annotation using CAFE 3.** *Mol Biol Evol* 2013, **30:**1987-1997.

53. Yang Z: **PAML 4: phylogenetic analysis by maximum likelihood.** *Mol Biol Evol* 2007, **24:**1586-1591.

54. Zhang G, Li C, Li Q, Li B, Larkin DM, Lee C, Storz JF, Antunes A, Greenwold MJ, Meredith RW: **Comparative genomics reveals insights into avian genome evolution and adaptation.** *Science* 2014, **346:**1311-1320.

55. Consortium TCSaA: **Initial sequence of the chimpanzee genome and comparison with the human genome.** *Nature* 2005, **437:**69-87.

56. Huang DW, Sherman BT, Lempicki RA: **Systematic and integrative analysis of large gene lists using DAVID bioinformatics resources.** *Nature protocols* 2008, **4:**44-57.

## Figure legends

**Figure 1 Comparative genome analyses of the *T. kingsejongensis* genome. (A)** Venn diagram of orthologous gene clusters between the four arthropod lineages. **(B)** Gene family gain-and-loss analysis. The number of gained gene families (red), lost gene families (blue) and orphan gene families (black) are indicated for each species. Time lines specify divergence times between the lineages.

**Figure 2 *Tigriopus kingsejongensis*-specific adaptive evolution. (A)** Global mean *w* distribution by GO categories of *T. kingsejongensis* and *T. japonicus*. GO categories showing supposedly accelerated nonsynonymous divergence (binomial test, test statistic $< 0.05$) in *T. kingsejongensis* and *T. japonicus* are colored in red and blue, respectively. **(B)** A total of seven enzyme-coding genes were PSGs involved in the four metabolic pathways (oval frame) of *T. kingsejongensis*: energy (purple), nucleotide (red), lipid (green), and carbohydrate (blue) metabolic pathways. The three genes belonging to the oxidative phosphorylation pathway (KEGG pathway map00190) (rectangular frame) are presented below the enzymes involved. Solid lines indicate direct processes and dashed lines indicate that more than one step is involved in a process.

**Figure 3 Comparing gene expression at different temperatures. (A)** RNA-seq analysis of upregulated *T. kingsejongensis* genes at 4°C and 15°C. **(B)** Transcription factors of four upregulated *TkTret* genes, and comparisons of transcription factor frequency between 2,276 upregulated genes at 4°C and the entire gene set. **(C)** Structure of transcription binding motifs of four upregulated *TkTret* genes. **(D)** Comparison of expression change for *Tret* transcription factors at 4°C and 15°C.

Figure 1

Click here to download Figure Fig1.tif

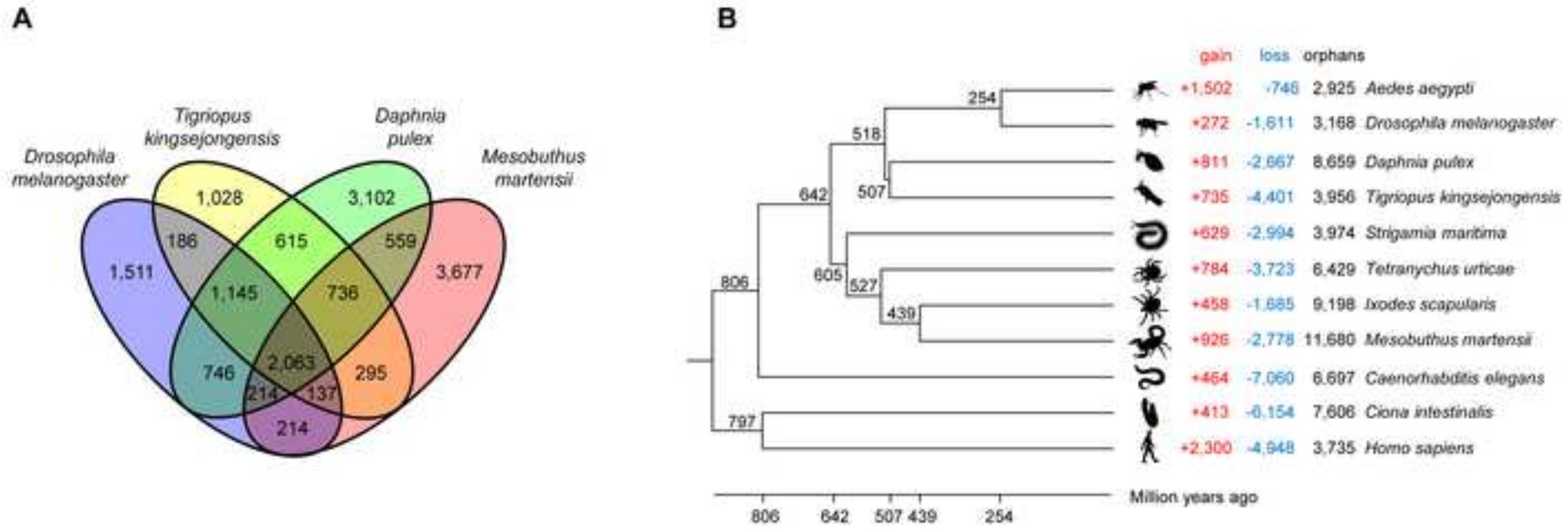

Figure 2

Click here to download Figure Fig2.tif

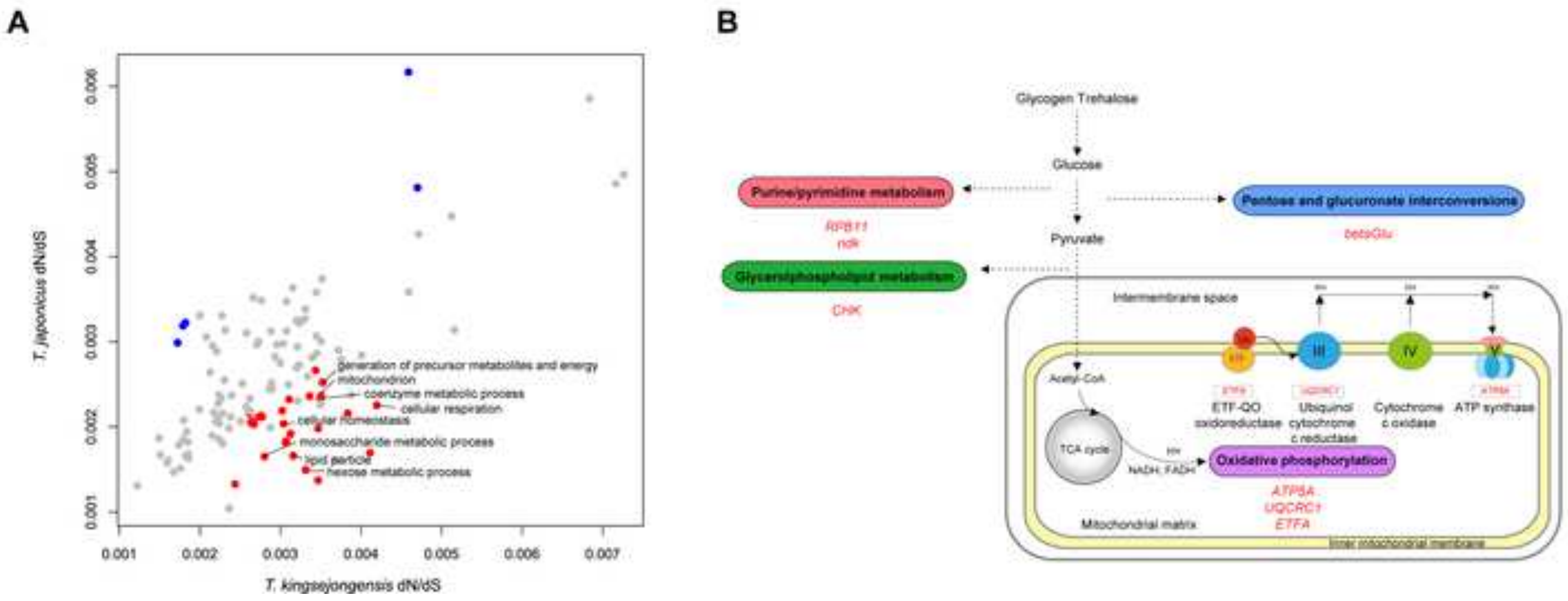

Figure 3

Click here to download Figure Fig3.tif

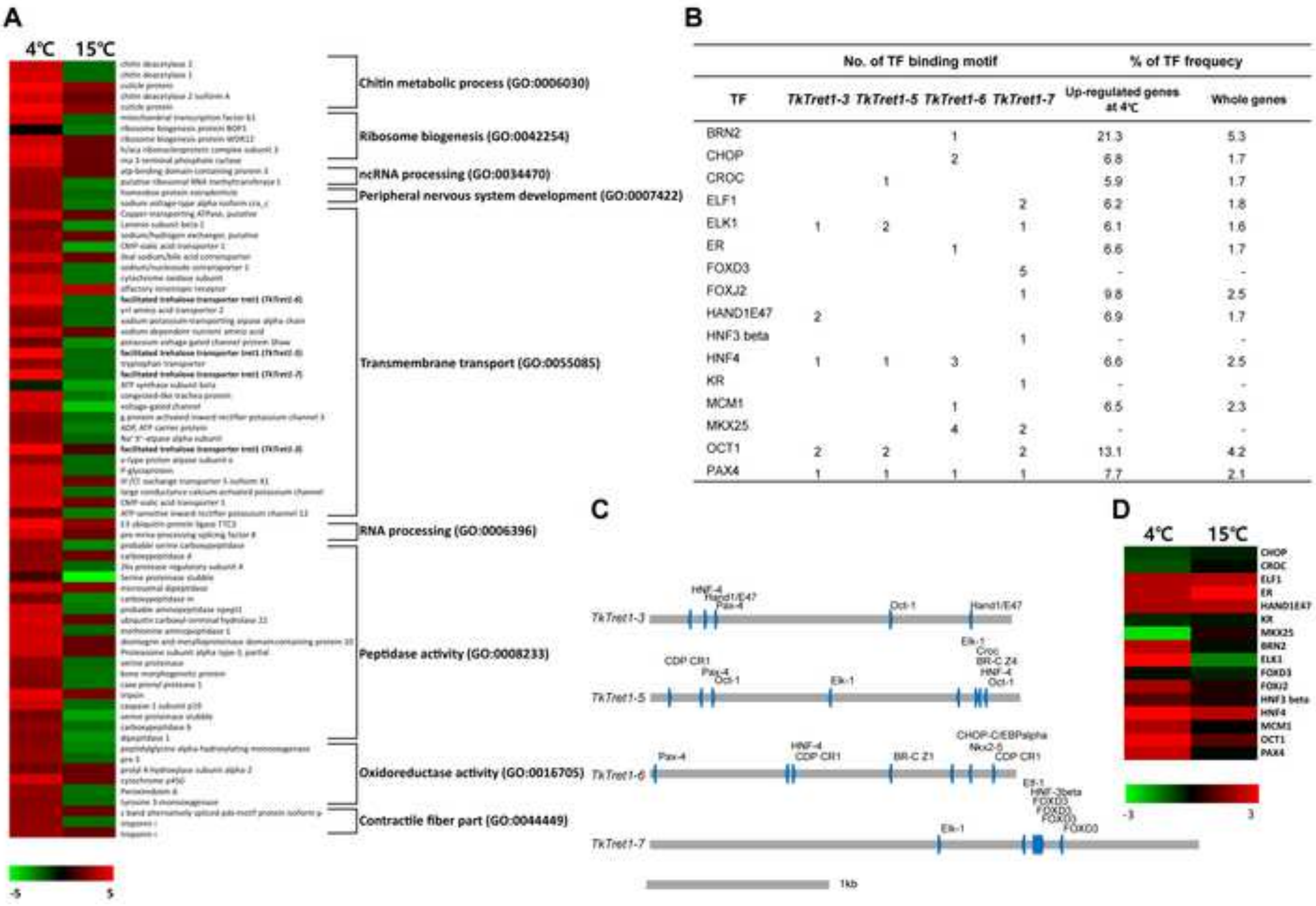


| | No. of TF binding motif | | | | % of TF frequency | |
|---|---|---|---|---|---|---|
| TF | *TkTret1-3* | *TkTret1-5* | *TkTret1-6* | *TkTret1-7* | Up-regulated genes at 4℃ | Whole genes |
| BRN2 | | | 1 | | 21.3 | 5.3 |
| CHOP | | | 2 | | 6.8 | 1.7 |
| CROC | | 1 | | | 5.9 | 1.7 |
| ELF1 | | | | 2 | 6.2 | 1.8 |
| ELK1 | 1 | 2 | | 1 | 6.1 | 1.6 |
| ER | | | 1 | | 6.6 | 1.7 |
| FOXD3 | | | | 5 | - | - |
| FOXJ2 | | | | 1 | 9.8 | 2.5 |
| HAND1E47 | 2 | | | | 6.9 | 1.7 |
| HNF3 beta | | | | 1 | - | - |
| HNF4 | 1 | 1 | 3 | | 6.6 | 2.5 |
| KR | | | | 1 | - | - |
| MCM1 | | | 1 | | 6.5 | 2.3 |
| MKX25 | | | 4 | 2 | - | - |
| OCT1 | 2 | 2 | | 2 | 13.1 | 4.2 |
| PAX4 | 1 | 1 | 1 | 1 | 7.7 | 2.1 |

Supplementary Material 1

Click here to access/download
**Supplementary Material**
Additional_file_1.docx

Supplementary Material 2

Click here to access/download
**Supplementary Material**
Additional_file_2.docx
