# Supplementary material for: The genome of the Antarctic-endemic copepod, Tigriopus kingsejongensis
Source: Gigascience. 2017 Jan 7;6(1):1–9. doi: 10.1093/gigascience/giw010 (PMC5467011; doi:10.1093/gigascience/giw010)

GigaScience

The genome of Antarctic-endemic Copepod, Tigriopus kingsejongensis

--Manuscript Draft--

**Manuscript Number:**	GIGA-D-16-00040R3
**Full Title:**	The genome of Antarctic-endemic Copepod, Tigriopus kingsejongensis
**Article Type:**	Data Note
**Funding Information:**	Korea Polar Research Institute (KR) (PE16070) — Dr. Hyun Park<br>Korea Polar Research Institute (PE14260) — Dr Sanghee Kim
**Abstract:**	Background: The Antarctic intertidal zone is continuously subject to extreme fluctuations in biotic and abiotic stressors, and the West Antarctic Peninsula is the most rapidly warming region on earth. Organisms living in Antarctic intertidal pools are therefore of great interest for research on topics such as evolutionary adaptation to extreme environments and the effects of climate change.<br>Findings: Here, we report the whole-genome sequence of the Antarctic endemic Harpacticoid copepod, Tigriopus kingsejongensis with a total of 37 Gb raw DNA sequence using Illumina Miseq platform and the libraries were prepared with 65-fold coverage with a total length of 295 Mb. The final assembly consists of 48,368 contigs with an N50 contig length of 17.5 kb and 27,823 scaffolds with N50 contig length of 159.2 kb and a total of 12,772 coding genes were inferred using the MAKER annotation pipeline approach. Comparative genome analysis revealed that T. kingsejongensis specific genes are enriched in transport and metabolism processes. Furthermore, rapidly evolving genes related to energy metabolism showed signatures of positive selection.<br>Conclusions: The genome of T. kingsejongensis will provide an interesting example of an evolutionary strategy for Antarctic cold adaptation, and offers new genetic insights into Antarctic intertidal biota.
**Corresponding Author:**	Hyun Park<br><br>KOREA, REPUBLIC OF
**Corresponding Author Secondary Information:**	
**Corresponding Author's Institution:**	
**Corresponding Author's Secondary Institution:**	
**First Author:**	Seunghyun Kang
**First Author Secondary Information:**	
**Order of Authors:**	Seunghyun Kang<br>Do-Hwan Ahn<br>Jun Hyuck Lee<br>Sung Gu Lee<br>Seung Chul Shin<br>Jungeun Lee<br>Gi-Sik Min<br>Hyoungseok Lee<br>Hyun-Woo Kim<br>Sanghee Kim<br>Hyun Park

Powered by Editorial Manager® and ProduXion Manager® from Aries Systems Corporation

**Order of Authors Secondary Information:**	
**Response to Reviewers:**	GIGA-D-16-00040<br>The genome of Antarctic-endemic Copepod, Tigriopus kingsejongensis<br>Seunghyun Kang; Do-Hwan Ahn; Jun Hyuck Lee; Sung Gu Lee; Seung Chul Shin; Jungeun Lee; Gi-Sik Min; Hyoungseok Lee; Hyun-Woo Kim; Sanghee Kim; Hyun Park<br>GigaScience<br><br>Dear Hans,<br><br>We would like to thank you and the reviewers for your kind help to revise our manuscript and consider our manuscript for publication in GigaScience. We appreciated all of the comments and suggestions and carefully considered all of them during the revision. All of inferred statements have been corrected and we have included the photo of the specimen as a main figure as you suggested. The corrected points were marked in blue color in revised manuscript.<br><br>We did our best to address the comments from the reviewers. Hope the revised is acceptable for publication. We look forward to hearing your decision.<br><br>Thanks and best regards.<br><br><br>Reviewer reports:<br>Reviewer #1: This paper is in reasonably good shape and the data will be useful for comparison to temperate Tigriopus. My only remaining comment is that the coverage of the CEGMA is relatively low and I don't know how that impacts the estimates of turnover in gene families (which the authors estimate to be quite high). Could this be a result of the fact that the assembly is missing a sizable proportion of core eukaryotic genes?<br><br>Response) We have added table 6 which summarizes genome completeness reports of T. kingsejongensis and other arthropod genomes used in gene family assignments. Overall, non-insect arthropod genomes showed relatively low CEGMA and BUSCO assignment scores. This tendency is commonly observed in non-insect genomes [1, 2] because the gene sets are mainly made of widely studied insect genomes. In “Annotation” and “Gene Families” part (which is in blue color), we made comment about this tendency and need to be careful to examine gene turnover events in non-insect arthropod genomes. Finally, we remarked necessity of globally approved arthropod orthologous gene sets to the field.<br><br>1.Hoy M, Waterhouse R, Wu K, Estep A, Ioannidis P, Palmer W, Pomerantz A, Simão F, Thomas J, Jiggins F: Genome sequencing of the phytoseiid predatory mite Metaseiulus occidentalis reveals completely atomised Hox genes and super-dynamic intron evolution. Genome biology and evolution 2016.<br><br>2.Simão FA, Waterhouse RM, Ioannidis P, Kriventseva EV, Zdobnov EM: BUSCO: assessing genome assembly and annotation completeness with single-copy orthologs. Bioinformatics 2015:btv351.
**Additional Information:**	
**Question**	**Response**
Are you submitting this manuscript to a special series or article collection?	No
**Experimental design and statistics**<br><br><br>Full details of the experimental design and statistical methods used should be given in the Methods section, as detailed in our Minimum Standards Reporting Checklist. Information essential to interpreting the	Yes

data presented should be made available in the figure legends.<br><br>Have you included all the information requested in your manuscript?	
**Resources**<br><br>A description of all resources used, including antibodies, cell lines, animals and software tools, with enough information to allow them to be uniquely identified, should be included in the Methods section. Authors are strongly encouraged to cite Research Resource Identifiers (RRIDs) for antibodies, model organisms and tools, where possible.<br><br>Have you included the information requested as detailed in our Minimum Standards Reporting Checklist?	Yes
**Availability of data and materials**<br><br>All datasets and code on which the conclusions of the paper rely must be either included in your submission or deposited in publicly available repositories (where available and ethically appropriate), referencing such data using a unique identifier in the references and in the "Availability of Data and Materials" section of your manuscript.<br><br>Have you have met the above requirement as detailed in our Minimum Standards Reporting Checklist?	Yes

Manuscript
Click here to view linked References
Click here to download Manuscript 20161018_Tigriopus_ms_plain text.docx

The genome of Antarctic-endemic Copepod, *Tigriopus kingsejongensis*

Seunghyun Kang[1¶], Do-Hwan Ahn[1¶], Jun Hyuck Lee[1,2], Sung Gu Lee[1,2], Seung Chul Shin[1], Jungeun Lee[1], Gi-Sik Min[3], Hyoungseok Lee[1], Hyun-Woo Kim[4*&], Sanghee Kim[5*&] & Hyun Park[1,2*&]

[1] Unit of Polar Genomics, Korea Polar Research Institute, Yeonsu-gu, Incheon, South Korea

[2] Polar Sciences, University of Science & Technology, Yuseong-gu, Daejeon, South Korea

[3] Department of Biological Sciences, Inha University, Incheon, South Korea

[4] Department of Marine Biology, Pukyong National University, Busan, South Korea

[5] Division of Polar Life Sciences, Korea Polar Research Institute, Yeonsu-gu, Incheon, South Korea

* Corresponding author

E-mail: kimhw@pknu.ac.kr; sangheekim@kopri.re.kr; hpark@kopri.re.kr

[¶]These authors contributed equally to this work.

[&]These authors also contributed equally to this work.

Abstract

Background: The Antarctic intertidal zone is continuously subject to extreme fluctuations in biotic and abiotic stressors, and the West Antarctic Peninsula is the most rapidly warming region on earth. Organisms living in Antarctic intertidal pools are therefore of great interest for research on topics such as evolutionary adaptation to extreme environments and the effects of climate change.

Findings: Here, we report the whole-genome sequence of the Antarctic endemic Harpacticoid copepod, *Tigriopus kingsejongensis* with a total of 37 Gb raw DNA sequence using Illumina Miseq platform and the libraries were prepared with 65-fold coverage with a total length of 295 Mb. The final assembly consists of 48,368 contigs with an N50 contig length of 17.5 kb and 27,823 scaffolds with N50 contig length of 159.2 kb and a total of 12,772 coding genes were inferred using the MAKER annotation pipeline approach. Comparative genome analysis revealed that *T. kingsejongensis* specific genes are enriched in transport and metabolism processes. Furthermore, rapidly evolving genes related to energy metabolism showed signatures of positive selection.

Conclusions: The genome of *T. kingsejongensis* will provide an interesting example of an evolutionary strategy for Antarctic cold adaptation, and offers new genetic insights into Antarctic intertidal biota.

Keywords: Copepoda, Genome, Antarctic, adaptation, *Tigriopus*

Data description

The subclass copepods are very diverse and approximately 12,000 copepod species have been described [1, 2]. They dominate the zooplankton community contributing about 70% of total zooplankton biomass [3] and play an important role in the marine meiobenthic food web linking between the phytoplankton and higher trophic levels [4]. Harpacticoid copepods of the genus *Tigriopus* Norman 1868 are dominant members of shallow supratidal rock pools worldwide. They are distributed among habitats that vary widely in salinity, temperature, desiccation risk, and UV radiation, and have been used as a model system to investigate topics such as osmoregulation [5], temperature adaptation [6, 7] and environmental toxicology [8]. As the genome resources of copepods has been publically available (*Tigriopus californicus* [http://i5k.nal.usda.gov/Tigriopus_californicus], *Tigriopus japonicus* [9], *Eurytemora affinis* [http://i5k.nal.usda.gov/Eurytemora_affinis] and salmon louse *Lepeophtheirus salmonis* [http://sealouse.imr.no/]), now it is possible to explore their fundamental biological processes and physiological responses to diverse environments.

Antarctica provides not only an extreme habitat for extant organisms, but also a model for research on evolutionary adaptations to cold environments [10, 11]. The Antarctic intertidal zone, particularly in the Western Antarctic Peninsula region, is one of the most extreme environments on earth. It also serves as a potential barometer for global climate changes, since it is the fastest-warming region on earth [12]. Antarctic intertidal species that have evolved stenothermal phenotypes through adaptation to a year-round climate of extreme cold may now face extinction by global warming. The response of these species to further warming in Western Antarctica is of serious concern; however, to date there have been few studies focusing on species from the Antarctic intertidal zone.

Tigriopus kingsejongensis was first found and recognized as a new endemic species in a rock pool in the Antarctic Peninsula, and is extremely cold-tolerant and can survive in frozen sea

water [13]. We observed the morphological differences, such as increased numbers of caudal setae in nauplii, optimal growth temperature (ca. 8°C) and developmental characteristics have been compared to those of the congener *Tigriopus japonicus*, which is found in the coastal area of the Yellow Sea. *Tigriopus kingsejongensis* has evolved to overcome the unique environmental constrains of Antarctica, and therefore provides an ideal experimental model for all aspects of research on extreme habitats. This species may represent a case of rapid speciation, since the intertidal zone on King George Island and surrounding areas did not exist before 10,000 years ago [14]. *Tigriopus kingsejongensis* likely evolved as a distinct species within this relatively short time period. Thus, inter- and intraspecies comparative analyses of Antarctic *Tigriopus* species will help define the trajectory of adaptation to the Antarctic environment and also provide insights into the genetic basis of *Tigriopus* divergence and evolution.

Library construction and sequencing

Tigriopus kingsejongensis were collected from tidal pools in Potter Cove, near King Sejong Station, on the northern Antarctic Peninsula (62°14'S, 58°47'W) (Fig. 1 and Fig. S1 in additional file1) in January 2013 with a hand-nets. Water temperatures were 1.6 ± 0.8°C during this month. High-molecular-weight genomic DNA from pooled *T. kingsejongensis* was extracted using the DNeasy Blood & Tissue Kit (Qiagen). For Illumina Miseq sequencing, four library types were constructed with 350, 400, 450, and 500 bp for paired-end libraries, and 3 kb and 8 kb for mate-pair libraries, prepared using the standard Illumina sample preparation methods (Table 1). All sequencing processes were performed according to the manufacturer's instructions (Illumina).

RNA was prepared from pooled *T. kingsejongensis* and *Tigriopus japonicus* specimens from two different temperature experiments (4°C and 15°C) using the RNeasy Mini Kit

(Qiagen). For Illumina Miseq sequencing, subsequent experiments were carried out under the manufacturer's instructions (Illumina). The *de novo* transcriptome assembly was performed with CLC Genomics Workbench, setting the minimum allowed contig length to 200 nucleotides. The assembly process generated 40,172 contigs with a max length of 23,942 bp and an N50 value of 1,093 bp. These generated contigs were used as reference sequences for mapping of trimmed reads, and fold changes in expression for each gene were calculated with a significance threshold of $P \leq 0.05$ using CLC Genomics Workbench (Table 2 and 3).

Genome assembly

First, k-mer analysis was conducted using jellyfish 2.2.5 [15] to estimate the genome size from DNA paired-end libraries. The estimateds genome size was 298 Mb with main peak at a depth of ~39x (Fig. 2). Then, assemblies were performed using a Celera Assembler with Illumina short reads [16]. Prior to assembly, Illumina reads were trimmed using the FASTX-Toolkit (http://hannonlab.cshl.edu/fastx_toolkit) with parameters -t 20, -l 70 and -Q 33, after which a paired sequence from trimmed Illumina reads was selected. Finally, trimmed Illumina reads with 65-fold coverage (insert sizes 350, 400, 450, and 500 bp) were obtained and converted to the FRG file format (required by the Celera assembler) using FastqToCA. Assembly was performed on a 96-processor workstation with Intel Xeon X7460 2.66 GHz processors and 1 terabyte RAM with the following parameters: overlapper = ovl, unitigger = bogart, utgGraphErrorRate = 0.03, utgGraphErrorLimit = 2.5, utgMergeErrorRate = 0.030, utgMergeErrorLimit = 3.25, ovlErrorRate = 0.1, cnsErrorRate = 0.1, cgwErrorRate = 0.1, merSize = 22, and doOverlapBasedTrimming = 1. The initial Celera assembly had a total size of 305 Mb, N50 contig size of 17,566 bp, and max contig size of 349.5 kb. Scaffolding was completed using the software SSPACE 2.0 scaffolder using mate-paired data [17].

Subsequently, we closed gaps using Gapfiller Ver.1.9 software with 65× trimmed Illumina reads with default settings [18]. *De novo* assembly of 203 million reads from paired-end libraries and mate-paired libraries yielded a draft assembly (65-fold coverage) with a total length of 295 Mb, and contig and scaffold N50 sizes of 17.6 kb and 159.2 kb, respectively (Table 4 and Fig. 3).

Annotation

We used MAKER for genome annotation [19]. MAKER is a portable and easily configurable genome annotation pipeline. MAKER first identified repetitive elements using RepeatMasker [20]. This masked genome sequence was used for *ab initio* gene prediction with SNAP software [21], after which alignment of expressed sequence tags with BLASTn and protein information from tBLASTx were included. We used the *de novo* repeat library of *T. kingsejongensis* from RepeatModeler for RepeatMasker; proteins from five species with data from *D. melanogaster, D. pulex, T. japonicus*, and *Tigriopus californicus* were included in the analysis. RNA-seq-based gene prediction was performed by aligning all RNA-seq data against the assembled genome using TopHat [22], and Cufflinks [23] was used to predict cDNAs from the resultant data. Next, MAKER polished the alignments using the program Exonerate [24], which provided integrated information to synthesize SNAP annotation. MAKER then selected and revised the final gene model considering all information. A total of 12,772 genes were predicted using MAKER in *T. kingsejongensis*. Annotated genes contained an average of 4.6 exons, with an average mRNA length of 1,090 bp. Additionally, 12,562 of 12,772 genes were assigned preliminary functions based on automated annotation using Blast2GO (Ver. 2.6.0) [25] (Fig. S2 and S3 in additional file 1). The Infernal software package (Ver. 1.1) [26] and covariance models (CMs) from the Rfam database [27] were used to identify other non-coding RNAs in the *T. kingsejongensis* scaffold. We identified putative tRNA genes using tRNAscan-

SE [28] (Table S1 in additional file 2). tRNAscan-SE uses a covariance model (CM) that scores candidates based on their sequence and predicted secondary structures.

Non-gap sequences occupied 284.8 Mb (96.5%), and simple sequence repeats (SSRs) were 1.2 Mb (0.4%) in total (Table S2 in additional file 2). Transposable elements (TEs) comprised 6.5 Mb, which is roughly 2.3% of the assembled genome (Table S2 and S3 in additional file 2). On the basis of homology and *ab initio* gene prediction, we found that the genome of *T. kingsejongensis* contains 12,772 protein-coding genes (Table 5). By assessing the quality of the annotated 12,772 gene models, we found that 11,686 protein-coding genes (91.5%) were supported by the RNA-seq data, of which, 7,325 (63%) showed similarity to proteins from other species. To estimate genome assembly and annotation completeness, we have used both Analysis of Core Eukaryotic Genes Mapping Approach (CEGMA) [29] and Benchmarking Universal Single-Copy Orthologs (BUSCO) [30] (Table 6). CEGMA report showed that 193 of 248 CEGMA score genes were fully annotated (77.8 % completeness) and 206 of 248 genes were partially annotated (83 % completeness). The similar approach BUSCO assessment employ lineage-specific profiles libraries such as eukaryotes, metazoans, and arthropods. BUSCO analysis showed that the genome assembly contains 71 % of complete and 6 % of partial Metazoan orthologous gene set. The BUSCO reports using arthropod gene set assigned poorly the most which contained 61.1% complete genes and 10.7 % partial genes. The CEGMA and BUSCO gene set were largely made of insects and the other non-insect arthropod genomes obtained similar low assignment scores. Overall, genome completeness reports revealed moderately complete *T. kingsejongenesis* genome in non-dipteran arthropod genomes.

Gene families

The orthologous groups were identified from 11 species (*T. kingsejongensis*, *Aedes aegypti*, *D. melanogaster*, *Ixodes scapularis*, *M. martensii*, *Strigamia martima*, *Tetranychus*

urticae, *D. pulex*, *Homo sapiens*, *Ciona intestinalis*, and *Caenorhabditis elegans*) (Table 7) using OrthoMCL [32] with standard parameters and options, and transcript variants other than the longest translation forms were removed. For *T. kingsejongensis*, the coding sequence from the MAKER annotation pipeline was used. The 1:1:1 single-copy orthologous genes were subjected to phylogenetic construction and divergence time estimation. Protein-coding genes were aligned using PRANK with the codon alignment option [33], and poorly aligned sequences with gaps were removed using Gblock under the codon model [34]. We constructed a maximum-likelihood phylogenetic tree using RAxML with 1,000 bootstrap values [35] and calibrated the divergence time between species with TimeTree [36]. Finally, the average gene gain/loss rate along the given phylogeny was identified using the program CAFÉ 3.1 [37]. We constructed orthologous gene clusters using four arthropod species (Antarctic copepod, *T. kingsejongensis*; scorpion, *Mesobuthus martensii*; fruit fly, *Drosophila melanogaster* and water flea, *Daphnia pulex*) to compare the genomic features and the adaptive divergence in the arthropods. In total, 2,063 gene families are shared by all four species, and 1,028 genes are specific to the Antarctic copepod. *Tigriopus kingsejongensis* shares 4,559 (73.5%) gene families with *D. pulex*, which belongs to the same crustacean lineage Vericrustacea, 3,531 (56.9%) with *D. melanogaster*, and 3,231 (52.1%) with *M. martensii* (Fig. 4A). Gene ontology (GO) analysis revealed that the 1,028 *T. kingsejongensis*-specific genes are enriched in transport (single-organism transport, GO: 0044765; transmembrane transport, GO: 0055085; ion transport, GO: 0006811; cation transport, GO: 0006812) and single-organism metabolic processes (GO: 0044710) (Table S4 and S5 in additional file 2). Subsequently, we performed gene gain-and-loss analysis on 11 representative species, and found that *T. kingsejongensis* gained 735 gene families and lost 4,401 gene families (Fig. 4B). This species exhibits a gene family turnover of 5,136, the largest value among the eight arthropods. The second largest value was obtained from *T. uticae* and the third was obtained from *M. martensii*. In table 6,

non-insect arthropod genomes were relatively poorly assigned with CEGMA or BUSCO sets. These gene sets are largely based on insects in arthropods and the assignment reports have tendency to have low assignment scores in non-insect or -dipteran genomes [30, 38, 39]. This implies that we need to carefully examine the gene family turnover in non-insect arthropod genomes and require globally approved arthropod orthologous gene sets.

We also analyzed expansion and contraction of the gene families (Table S6-S9 in additional file 2), and found 232 significantly expanded gene families in *T. kingsejongensis*; these gene families are significantly overrepresented in amino acid metabolism and carbohydrate metabolism in KEGG metabolic pathways.

Genome evolution

Adaptive functional divergence caused by natural selection is commonly estimated based on the ratio of nonsynonymous (*dN*) to synonymous (*dS*) mutations. To estimate *dN*, *dS*, and average *dN*/*dS* ratio (*w*), and lineage-specific PSGs in *T. kingsejongensis* and *T. japonicus*, protein-coding genes from *T. japonicus* were added to define orthologous gene families among the four species (*T. kingsejongensis*, *T. japonicus*, *D. pulex*, and *D. melanogaster*) using the program OrthoMCL with the same conditions previously described. We identified 2,937 orthologous groups shared by all four species, and single-copy gene families were used to construct a phylogenetic tree and estimate the time since divergence using the same methods described above. Each of the identified orthologous genes was aligned using the PRANK, and poorly aligned sequences with gaps were removed using Gblock. Alignments showing less than 40% identity and genes shorter than 150 bp were eliminated in subsequent procedures. The values of *dN*, *dS* and *w* were estimated from each gene using the Codeml program implemented

in the PAML package with the free-ratio model [40] under F3X4 codon frequencies, and orthologs with $w \leq 5$ and $dS \leq 3$ were retained [41]. To examine the accelerated nonsynonymous divergence in either *T. kingsejongensis* or *T. japonicus* lineage, a binomial test [42] was used to determine GO categories with at least 20 orthologous genes. To define PSGs in *T. kingsejongensis* and *T. japonicus*, we applied basic and branch-site models, and Likelihood Ratio Tests (LRTs) were used to remove genes under relaxation of selective pressure. To investigate which functional categories and pathways were enriched in the PSGs, we performed DAVID Functional Annotation [43] with Fisher's exact test (cutoff: $P \leq 0.05$).

The average *w* value from 2,937 co-orthologous genes of *T. kingsejongensis* (0.0027) is higher than that of *T. japonicus* (0.0022). The GO categories that showing evidence of accelerated evolution in *T. kingsejongensis* are energy metabolism (generation of precursor metabolites and energy, GO: 0006091; cellular respiration, GO: 0045333) and carbohydrate metabolism (monosaccharide metabolic process, GO: 0005996; hexose metabolic process, GO: 0019318) (Fig. 5A, Table S10 in Additional file 2). Branch-site model analysis showed that the genes belonging to the functional categories above have undergone a significant positive selection process by putative functional divergence in certain lineages. There are 74 and 79 positively selected genes (PSGs) in *T. kingsejongensis* (Table S11 in Additional file 2) and *T. japonicus* (Table S12 in Additional file 2), respectively. The functional categories enriched in *T. kingsejongensis*, when compared to *T. japonicus*, support the idea that the functional divergence in *T. kingsejongensis* is strongly related to energy metabolism (oxidative phosphorylation, GO: 0006119; energy-coupled proton transport down electrochemical gradient, GO: 0015985; ATP synthesis-coupled proton transport, GO: 0015986; generation of precursor metabolites and energy, GO: 0006091) (Fig. 5B, Table S13 and S14 in Additional file 2). In particular, three of the identified genes are involved in the oxidative phosphorylation

(OxPhos) pathway, which provides the primary cellular energy source in the form of adenosine triphosphate (ATP). These three genes are nuclear-encoded mitochondrial genes: the catalytic F1 ATP synthase subunit alpha (*ATP5A*) (Fig. S4 in Additional file 1), a regulatory subunit acting as an electron transport chain such as ubiquinol-cytochrome *c* reductase core protein (*UQCRC1*) (Fig. S5 in Additional file 1), and an electron transfer flavoprotein alpha subunit (*ETFA*) (Fig. S6 in Additional file 1).

Availability of supporting data

The data for *T. kingsejongensis* genome and transcriptome has been deposited in the SRA as BioProject PRJNA307207 and PRJNA307513, respectively.

List of abbreviations

simple sequence repeats, SSRs; Transposable elements, TEs; CEGMA, Core Eukaryotic Genes Mapping Approach; BUSCO, Benchmarking Universal Single-Copy Orthologs ; Gene ontology, GO

Competing interests

The authors declare no competing interests.

Funding

This work was supported by an Antarctic organisms: Cold-adaptation mechanism and its application grant (PE16070) and the basic research program (PE14260) funded by the Korea Polar Research Institute (KOPRI).

Author contributions

H.P., Sanghee Kim and H.W.K. conceived and designed experiments and analyses; Seunghyun Kang, D.-H.A., S.G.L., S.C.S., J.L., G.S.M. and H.L. performed experiments and conducted

bioinformatics. Seunghyun Kang, H.W.K., Sanghee Kim and H.P. wrote the paper.

Acknowledgements

We would like to thank Joseph A. Covi for comments and discussion.

References

1. Huys R, Boxshall GA: *Copepod evolution.* Ray Society; 1991.
2. Humes AG: **How many copepods?** *Hydrobiologia* 1994, **292:**1-7.
3. Wells P, Persoone G, Jaspers E, C. C: *Marine ecotoxicological tests with zooplankton. In: Persoone, G., Jaspers, E., Claus, C. (Eds.), Ecotoxicological Testing for the Marine Environment.* Inst. Mar. Sci. Res., Bredene; 1984.
4. Ruppert E, Fox R, Barnes R: **Invertebrate Zoology, A Functional Evolutionary Approach. Brooks/Cole-Thomson Learning.** *Belmont, CA* 2003.
5. Goolish E, Burton R: **Energetics of osmoregulation in an intertidal copepod: Effects of anoxia and lipid reserves on the pattern of free amino accumulation.** *Funct Ecol* 1989**:**81-89.
6. Lazzaretto I, Libertini A: **Karyological comparison among different Mediterranean populations of the genus *Tigriopus* (Copepoda Harpacticoida).** *Boll Zool* 2009, **53:**197-201.
7. Davenport J, Barnett P, McAllen R: **Environmental tolerances of three species of the harpacticoid copepod genus *Tigriopus*.** *J Mar Biol Assoc UK* 1997, **77:**3-16.
8. Raisuddin S, Kwok KW, Leung KM, Schlenk D, Lee J-S: **The copepod *Tigriopus*: A promising marine model organism for ecotoxicology and environmental genomics.** *Aquat Toxicol* 2007, **83:**161-173.
9. Lee J-S, Rhee J-S, Kim R-O, Hwang D-S, Han J, Choi B-S, Park GS, Kim I-C, Park HG, Lee Y-M: **The copepod Tigriopus japonicus genomic DNA information (574Mb) and molecular anatomy.** *Mar Environ Res* 2010, **69:**S21-S23.
10. Thorne MAS, Kagoshima H, Clark MS, Marshall CJ, Wharton DA: **Molecular analysis of the cold tolerant Antarctic Nematode, *Panagrolaimus davidi*.** *PLOS one* 2014, **9:**e104526.

11. Everatta MJ, Worlandb MR, Balea JS, Conveyb P, Hayward SAL: **Pre-adapted to the maritime Antarctic? – Rapid cold hardening of the midge, *Eretmoptera murphyi*.** *J Insect Physiol* 2012, **58:**1104–1111.

12. Bromwich DH, Nicolas JP, Monaghan AJ, Lazzara MA, Keller LM, Weidner GA, Wilson AB: **Central West Antarctica among the most rapidly warming regions on Earth.** *Nature Geoscience* 2013, **6:**139-145.

13. Park E-O, Lee S, Cho M, Yoon SH, Lee Y, Lee W: **A new species of the genus *Tigriopus* (Copepoda: Harpacticoida: Harpacticidae) from Antarctica.** *Proc Biol Soc Wash* 2014, **127:**138-154.

14. Birkenmajer K: **Geology of Admiralty Bay, King George Island (South Shetland Islands). An outline.** *Pol Polar Res* 1980, **1:**29-54.

15. Marçais G, Kingsford C: **A fast, lock-free approach for efficient parallel counting of occurrences of k-mers.** *Bioinformatics* 2011, **27:**764-770.

16. Myers EW, Sutton GG, Delcher AL, Dew IM, Fasulo DP, Flanigan MJ, Kravitz SA, Mobarry CM, Reinert KH, Remington KA, et al: **A whole-genome assembly of *Drosophila*.** *Science* 2000, **287:**2196-2204.

17. Boetzer M, Henkel CV, Jansen HJ, Butler D, Pirovano W: **Scaffolding pre-assembled contigs using SSPACE.** *Bioinformatics* 2011, **27:**578-579.

18. Nadalin F, Vezzi F, Policriti A: **GapFiller: a *de novo* assembly approach to fill the gap within paired reads.** *BMC Bioinformatics* 2012, **13:**S8.

19. Holt C, Yandell M: **MAKER2: an annotation pipeline and genome-database management tool for second-generation genome projects.** *BMC Bioinformatics* 2011, **12:**491.

20. Smit AFA HR, Green, P.: **RepeatMasker Open-3.0. 1996-2004 (http://www.RepeatMakser.org).**

21. Korf I: **Gene finding in novel genomes.** *BMC Bioinformatics* 2004, **5:**59.

22. Trapnell C, Pachter L, Salzberg SL: **TopHat: discovering splice junctions with RNA-Seq.** *Bioinformatics* 2009, **25:**1105-1111.

23. Trapnell C, Williams BA, Pertea G, Mortazavi A, Kwan G, van Baren MJ, Salzberg SL, Wold BJ, Pachter L: **Transcript assembly and quantification by RNA-Seq reveals unannotated transcripts and isoform switching during cell differentiation.** *Nat Biotech* 2010, **28:**511-515.

24. Slater GS, Birney E: **Automated generation of heuristics for biological sequence comparison.** *BMC Bioinformatics* 2005, **6:**31.

25. Conesa A, Gotz S, Garcia-Gomez JM, Terol J, Talon M, Robles M: **Blast2GO: a universal tool for annotation, visualization and analysis in functional genomics research.** *Bioinformatics* 2005, **21:**3674-3676.

26. Nawrocki EP, Kolbe DL, Eddy SR: **Infernal 1.0: inference of RNA alignments.** *Bioinformatics* 2009, **25:**1335-1337.

27. Gardner PP, Daub J, Tate J, Moore BL, Osuch IH, Griffiths-Jones S, Finn RD, Nawrocki EP, Kolbe DL, Eddy SR, Bateman A: **Rfam: Wikipedia, clans and the "decimal" release.** *Nucleic Acids Res* 2011, **39:**D141-145.

28. Lowe TM, Eddy SR: **tRNAscan-SE: a program for improved detection of transfer RNA genes in genomic sequence.** *Nucleic Acids Res* 1997, **25:**955-964.

29. Parra G, Bradnam K, Korf I: **CEGMA: a pipeline to accurately annotate core genes in eukaryotic genomes.** *Bioinformatics* 2007, **23:**1061-1067.

30. Simão FA, Waterhouse RM, Ioannidis P, Kriventseva EV, Zdobnov EM: **BUSCO: assessing genome assembly and annotation completeness with single-copy orthologs.** *Bioinformatics* 2015**:**btv351.

31. Chipman AD, Ferrier DE, Brena C, Qu J, Hughes DS, Schröder R, Torres-Oliva M,

Znassi N, Jiang H, Almeida FC: **The first myriapod genome sequence reveals conservative arthropod gene content and genome organisation in the centipede *Strigamia maritima*.** *PLoS Biol* 2014, **12:**e1002005.

32. Li L, Stoeckert CJ, Roos DS: **OrthoMCL: identification of ortholog groups for eukaryotic genomes.** *Genome Res* 2003, **13:**2178-2189.

33. Löytynoja A, Goldman N: **An algorithm for progressive multiple alignment of sequences with insertions.** *Proc Natl Acad Sci U S A* 2005, **102:**10557-10562.

34. Castresana J: **Selection of conserved blocks from multiple alignments for their use in phylogenetic analysis.** *Mol Biol Evol* 2000, **17:**540-552.

35. Stamatakis A: **RAxML version 8: a tool for phylogenetic analysis and post-analysis of large phylogenies.** *Bioinformatics* 2014, **30:**1312-1313.

36. Hedges SB, Dudley J, Kumar S: **TimeTree: a public knowledge-base of divergence times among organisms.** *Bioinformatics* 2006, **22:**2971-2972.

37. Han MV, Thomas GW, Lugo-Martinez J, Hahn MW: **Estimating gene gain and loss rates in the presence of error in genome assembly and annotation using CAFE 3.** *Mol Biol Evol* 2013, **30:**1987-1997.

38. Rider SD, Morgan MS, Arlian LG: **Draft genome of the scabies mite.** *Parasites & Vectors* 2015, **8:**585.

39. Hoy M, Waterhouse R, Wu K, Estep A, Ioannidis P, Palmer W, Pomerantz A, Simão F, Thomas J, Jiggins F: **Genome sequencing of the phytoseiid predatory mite Metaseiulus occidentalis reveals completely atomised Hox genes and super-dynamic intron evolution.** *Genome biology and evolution* 2016.

40. Yang Z: **PAML 4: phylogenetic analysis by maximum likelihood.** *Mol Biol Evol* 2007, **24:**1586-1591.

41. Zhang G, Li C, Li Q, Li B, Larkin DM, Lee C, Storz JF, Antunes A, Greenwold MJ,

Meredith RW: **Comparative genomics reveals insights into avian genome evolution and adaptation.** *Science* 2014, **346:**1311-1320.

42. Consortium TCSaA: **Initial sequence of the chimpanzee genome and comparison with the human genome.** *Nature* 2005, **437:**69-87.

43. Huang DW, Sherman BT, Lempicki RA: **Systematic and integrative analysis of large gene lists using DAVID bioinformatics resources.** *Nature protocols* 2008, **4:**44-57.

Figure legends

Figure 1 Image of an adult *Tigriopus kingsejongensis* specimen.

Figure 2 Estimation of the *T. kingsejongensis* genome size based on 33-mer analysis. The x-axis represents the depth (peak at 39X) and the y-axis represents the proportion. The genome size was estimated as 298 Mb (total k-mer number/volume peak).

Figure 3 Scaffold and contig size distributions of *T. kingsejongensis.* The percentage of the assembly included (y-axis) in contigs or scaffolds of a minimum size (x-axis, log scale) is shown for the contig (red) and scaffold (blue).

Figure 4 Comparative genome analyses of the *T. kingsejongensis* genome. **a** Venn diagram of orthologous gene clusters between the four arthropod lineages. **b** Gene family gain-and-loss analysis. The number of gained gene families (red), lost gene families (blue) and orphan gene families (black) are indicated for each species. Time lines specify divergence times between the lineages.

Figure 5 *Tigriopus kingsejongensis*-specific adaptive evolution. **a** Global mean *w* distribution by GO categories of *T. kingsejongensis* and *T. japonicus*. GO categories showing supposedly accelerated nonsynonymous divergence (binomial test, test statistic < 0.05) in *T. kingsejongensis* and *T. japonicus* are colored in red and blue, respectively. **b** A total of seven enzyme-coding genes were PSGs involved in the four metabolic pathways (oval frame) of *T. kingsejongensis*: energy (purple), nucleotide (red), lipid (green), and carbohydrate (blue) metabolic pathways. The three genes belonging to the oxidative phosphorylation pathway

(KEGG pathway map00190) (rectangular frame) are presented below the enzymes involved. Solid lines indicate direct processes and dashed lines indicate that more than one step is involved in a process.

Table legends

Table 1 Statistics for each DNA library.

Table 2 Sequencing and assembly results of transcriptome analysis of *T. japonicus.*

Table 3 Sequencing statistics of RNA-seq analysis of *T. kingsejongensis*.

Table 4 Statistics of genome assembly.

Table 5 General statistics of genes in *T. kingsejongensis.*

Table 6 *Tigriopus kingsejongensis* genome completeness reports with the other arthropod genomes.

Table 7 Summary of orthologous gene clusters in the 11 representative species.

Table 1 Statistics for each DNA library.

**Library**		**Reads (n)**	**Ave. length**	**Sequences (bp) (n)**	**Reads (trimmed) (n)**	**Ave. length**	**Sequences (trimmed) (n)**
Paired-end	Sum	99,710,266		29,271,916,613	65,644,374		14,668,956,871
	350S1	6,661,392	300	2,005,078,992	4,446,394	233	1,034,231,244
	350S2	4,933,058	265	1,311,700,122	4,618,711	211	975,471,763
	400S1	65,668,598	300	19,766,247,998	36,863,154	228	8,397,426,481
	450S1	3,418,988	300	1,029,115,388	2,812,455	230	646,302,159
	450S2	8,009,162	245	1,968,652,020	7,660,814	199	1,527,566,312
	500S1	11,019,068	289	3,191,122,093	9,242,846	226	2,087,958,911
Mate-Paired	Sum	103,373,998		7,753,049,850	73,515,391		5,169,006,268
	3KS1	8,374,238	75	628,067,850	6,745,546	73	493,099,413
	3KS2	9,250,994	75	693,824,550	5,281,513	65	344,618,723
	3KS3	51,349,594	75	3,851,219,550	39,147,167	72	2,816,638,666
	3KS4	3,063,232	75	229,742,400	1,740,986	65	112,554,745
	8KS1	9,847,636	75	738,572,700	7,887,612	73	572,246,251
	8KS2	16,322,038	75	1,224,152,850	9,653,293	65	630,842,698
	8KS3	5,166,266	75	387,469,950	3,059,274	65	199,005,774
Total		203,084,264		37,024,966,463	139,159,765		19,837,963,139
Coverage (folds)				120.7			64.7

Table 2 Sequencing and assembly results of transcriptome analysis of *T. japonicus*.

**Sequencing**	
Total reads (n)	37,956,160
Total bases (n)	7,714,415,316
Trimmed reads (n)	35,577,636
Trimmed bases (n)	5,989,188,343
**Assembly**	
Contigs (n)	40,172
Total contig length (bases)	28,850,726
N50 contig length (bases)	1,093
Max scaffold length (bases)	23,942
**Annotation**	
With blast results	20,392
Without blast hits	7,090
With mapping results	8,172
Annotated sequences	4,518

Table 3 Sequencing statistics of RNA-seq analysis of *T. kingsejongensis*.

	**4°C**	**15°C**
Total reads (n)	15,786,118	16,417,072
Total bases (n)	3,567,662,668	3,763,295,032
Trimmed reads (n)	14,845,103	15,388,513
Trimmed bases (n)	2,761,189,158	2,833,805,442

Table 4 Statistics of genome assembly.

		**Celera assembler (Version : 8.0)**
Scaffold	Total scaffold length (bases)	295,233,602
	Gap size (bases)	10,474,460
	Scaffolds (n)	11,558
	N50 scaffold length (bases)	159,218
	Max scaffold length (bases)	3,401,446
Contig	Total contig length (bases)	305,712,242
	Contigs (n)	48,368
	N50 contig length (bases)	17,566
	Max contig length (bases)	349,507

Table 5 General statistics of genes in *T. kingsejongensis*.

Genes (n)	12,772
Gene Length Sum (bp)	82,293,116
Exons per genes (n)	4.6
mRNA Length Sum (bp)	43,306,342
Average mRNA length (bp)	1,090
Number of tRNA	1,393
Number of rRNA	215

Click here to download Table Table 6.docx

Table 6 *Tigriopus kingsejongensis* genome completeness reports with the other arthropod genomes.

**Species**	***Tigriopus kingsejongensis***	***Daphnia pulex***	***Ixodes scapularis***	***Mesobuthus martensii***	***Strigamia maritima***	***Tetranychus urticae***	***Drosophila melanogaster***	***Aedes aegypti***
**Assembly**	This study	GCA_000187875.1	GCA_000208615.1	GCA_000484575.1	Smar1.22	GCA_000239435.1	Dmel_r5.55	AaegL3
**Sample type**	genome	genome	genome	genome	genome	genome	genome	genome
**CEGMA**[a]	83/77.8	99.2/98.8	79.8/41.9 [g]	57.3/24.2 [g]	95.1 [f]	98.0/95.2 [g]	100/100	99.2/83.5
**BUSCO**[b]	61.1 [10.5], 10.7, 28.1	83 [3.9], 11, 5.1 [e]	68.9 [2.4], 21.0, 10.1 [g]	34.4 [4.0], 23.0, 42.7 [g]	84 [5.9], 12, 3.2 [e]	68.8 [5.8], 9.9, 21.3 [g]	98 [6.4], 0.6, 0.3 [e]	86 [13], 10, 3.2 [e]
**BUSCO**[c]	70.9 [13.6], 6.0, 23.0							
**BUSCO**[d]	67.1 [16.8], 5.1, 27.7							

[a] 248 CEGMA genes Found/Complete

[b] BUSCO Arthropods Complete [Duplicated], Fragmented, Missing

[c] BUSCO Metazoan Complete [Duplicated], Fragmented, Missing

[d] BUSCO Eukaryotes Complete [Duplicated], Fragmented, Missing

[e] [30]

[f] [31]

[g] [39]

Table 7 Summary of orthologous gene clusters in the 11 representative species.

Species	Source of data	No. of coding genes	No. of gene families	No. of genes in gene families	No. of orphan genes	No. of unique gene families	Average No. of genes in gene families
Aedes aegypti	Ensembl genome 25	15,797	7,958	12,792	7,839	854	1.61
Caenorhabditis elegans	Ensembl gene 78	20,447	6,536	13,737	13,911	1,528	2.10
Ciona intestinalis	Ensembl gene 78	16,671	7,017	9,058	9,654	503	1.29
Daphnia pulex	Ensembl genome 25	30,590	6,710	8,362	7,208	368	1.25
Drosophila melanogaster	Ensembl gene 78	13,918	9,673	21,917	20,917	2,408	2.27
Homo sapiens	Ensembl gene 78	20,300	8,696	17,186	11,604	1,065	1.98
Ixodes scapularis	Ensembl genome 25	20,486	8,097	11,277	12,389	873	1.39
Mesobuthus martensii	http://lifecenter.sgst.cn /main/en/scorpion.jsp	32,016	8,389	19,961	23,627	2,276	2.38
Strigamia maritima	Ensembl genome 25	14,992	7,727	11,012	7,265	583	1.43
Tetranychus urticae	Ensembl genome 25	18,224	6,602	11,788	11,622	939	1.79
T. kingsejongensis	this study	12,772	6,205	8,813	6,567	649	1.42

Figure 1

Click here to download Figure Fig1.tif

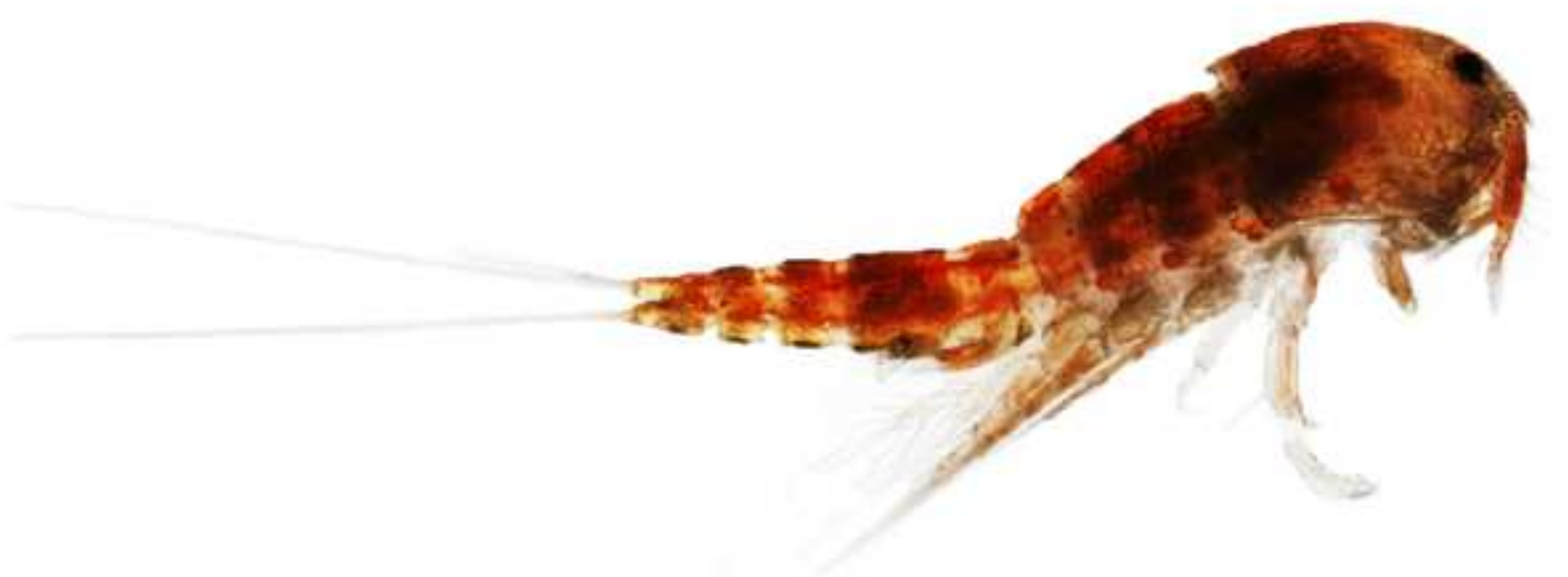

Figure 2

Click here to download Figure Fig2.tif

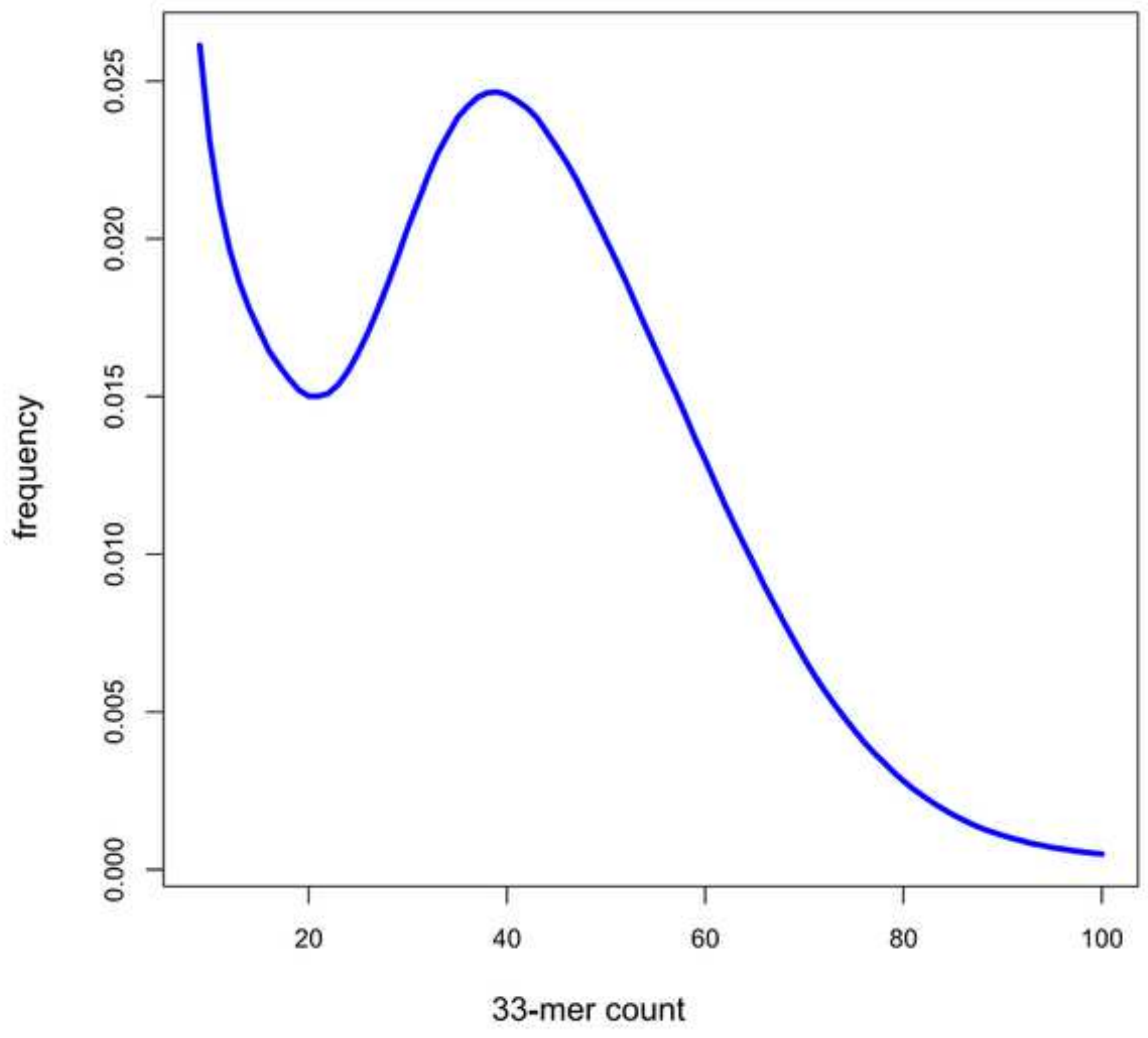

Figure 3

Click here to download Figure Fig3.tif

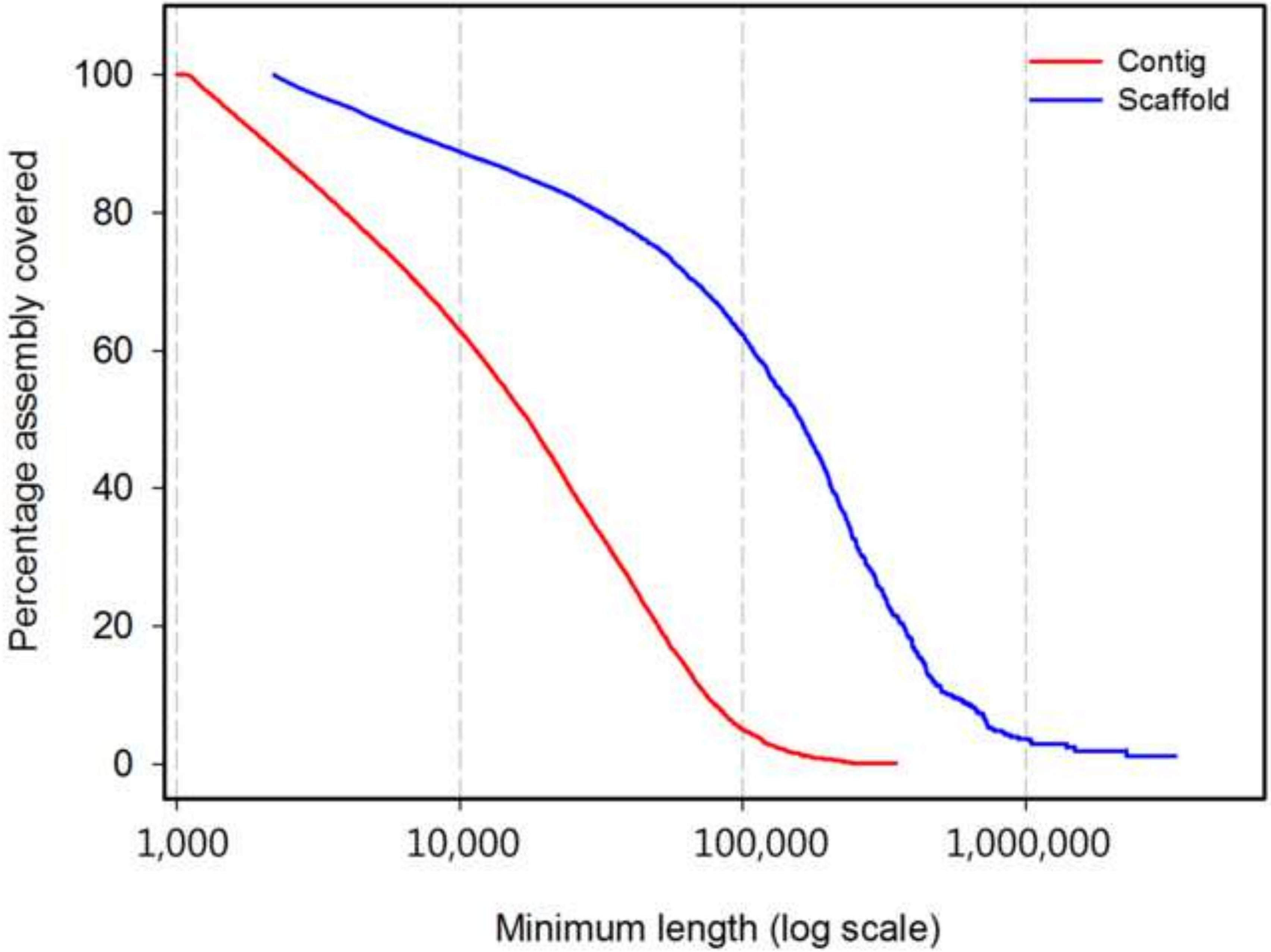

Figure 4 Click here to download Figure Fig4.tif

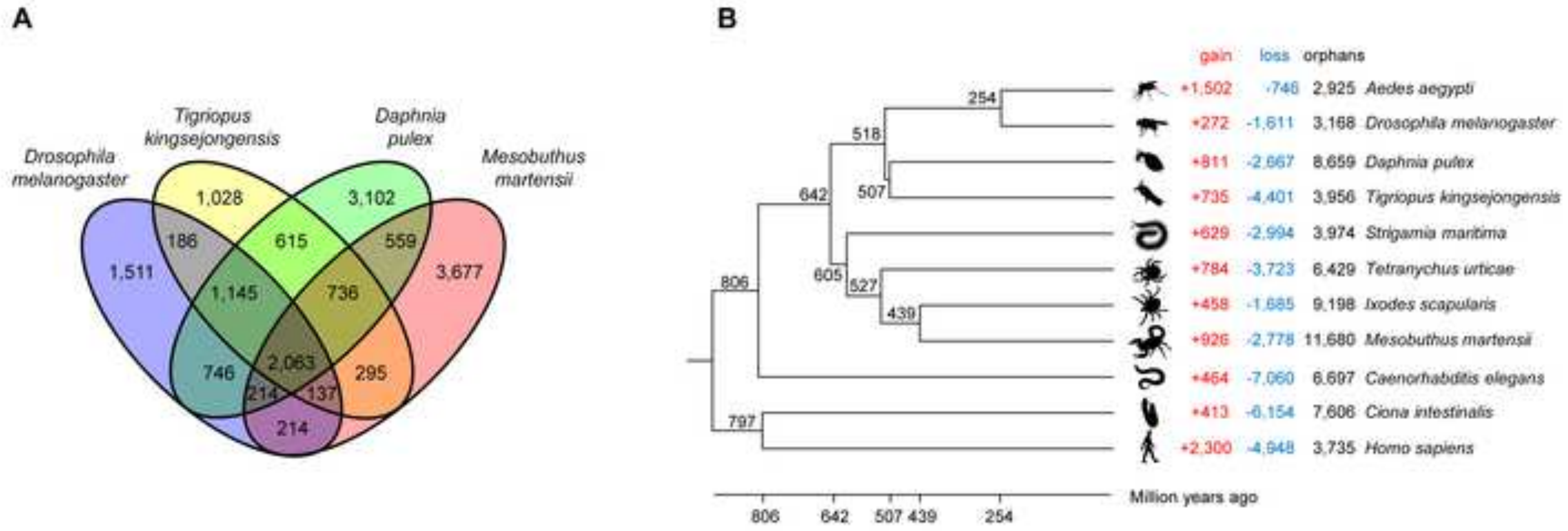

Figure 5

Click here to download Figure Fig5.tif

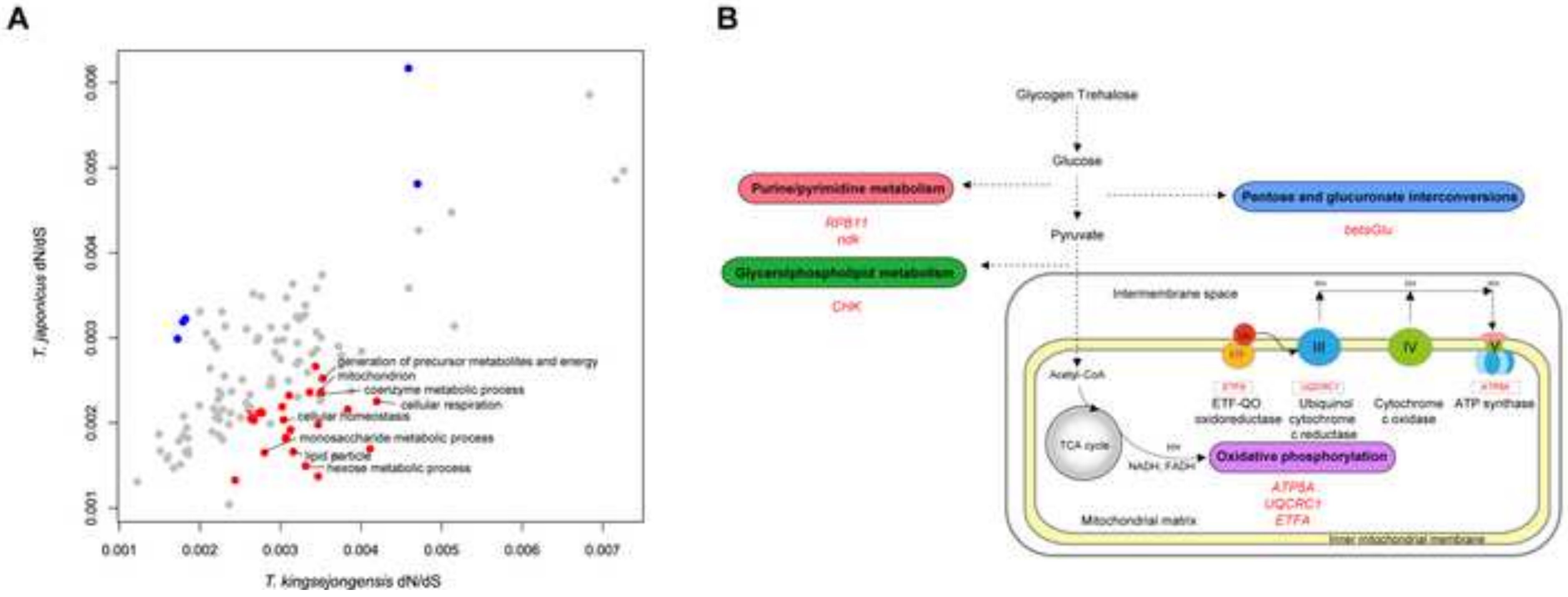

Supplementary Figures

Click here to access/download
Supplementary Material
Additional_file_1.docx

Click here to access/download
Supplementary Material
Additional_file_2.docx

Click here to download Personal Cover Cover image.tif

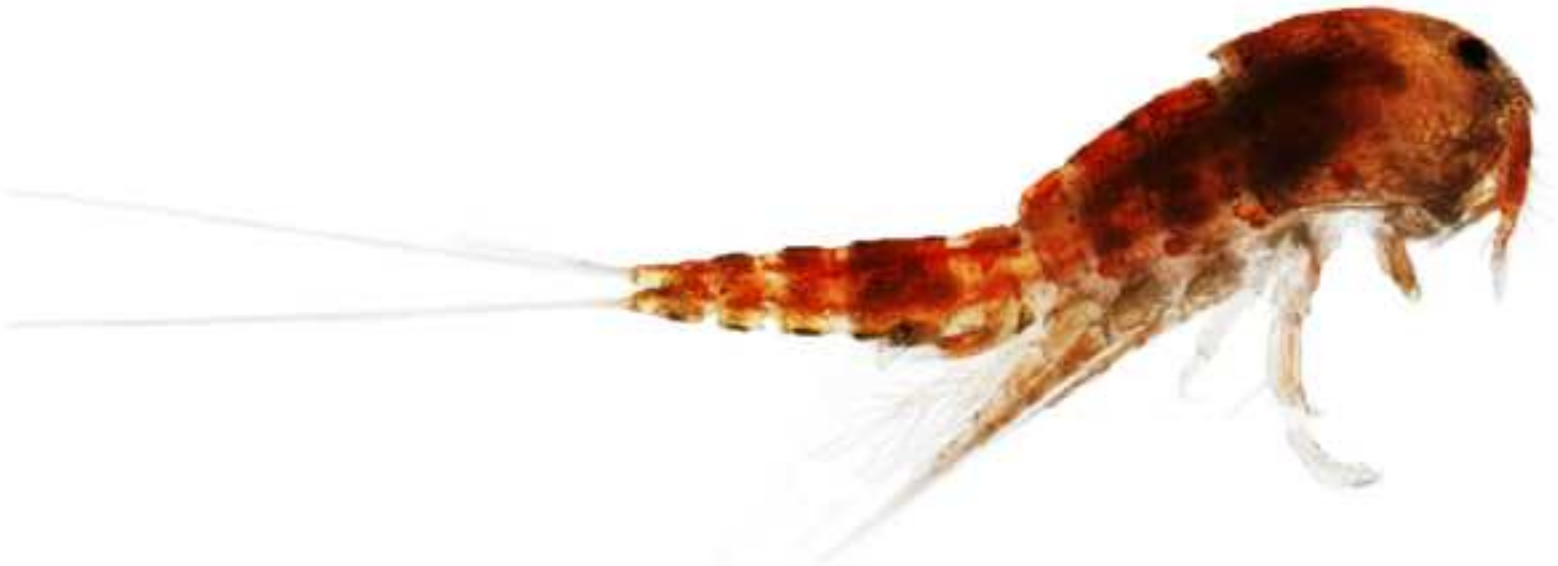

Supplement: GIGA-D-16-00040_Revision_3.pdf [file giw010_GIGA-D-16-00040_Revision_3.pdf]
