# Supplementary figures and images for: The genome of the Antarctic-endemic copepod, Tigriopus kingsejongensis
Source: Gigascience. 2017 Jan 7;6(1):1–9. doi: 10.1093/gigascience/giw010 (PMC5467011; doi:10.1093/gigascience/giw010)

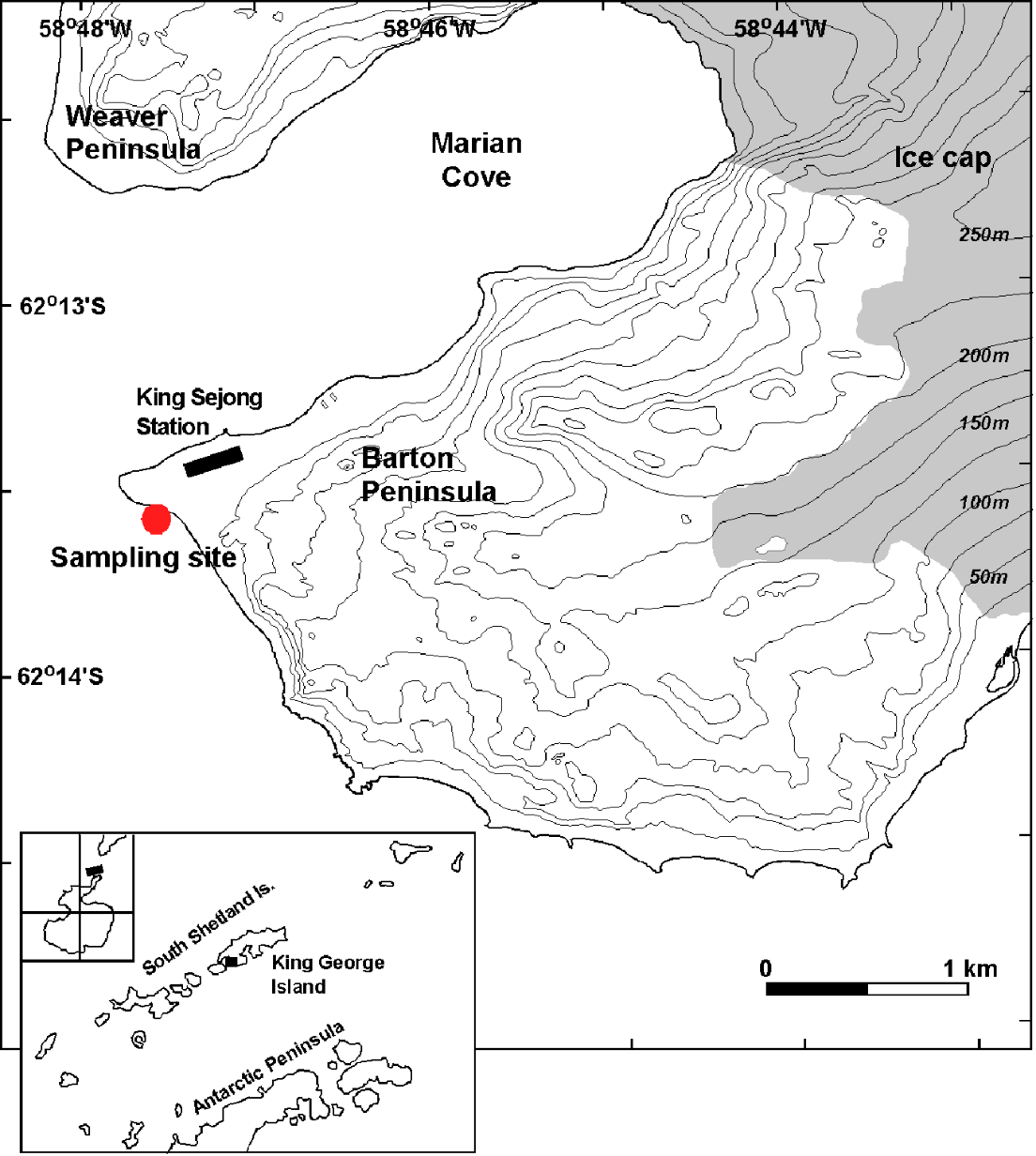

Supplement: Figure S1. — Map showing location of the Tigriopus kingsejongensis sampling site. [file giw010_FigS1.png]

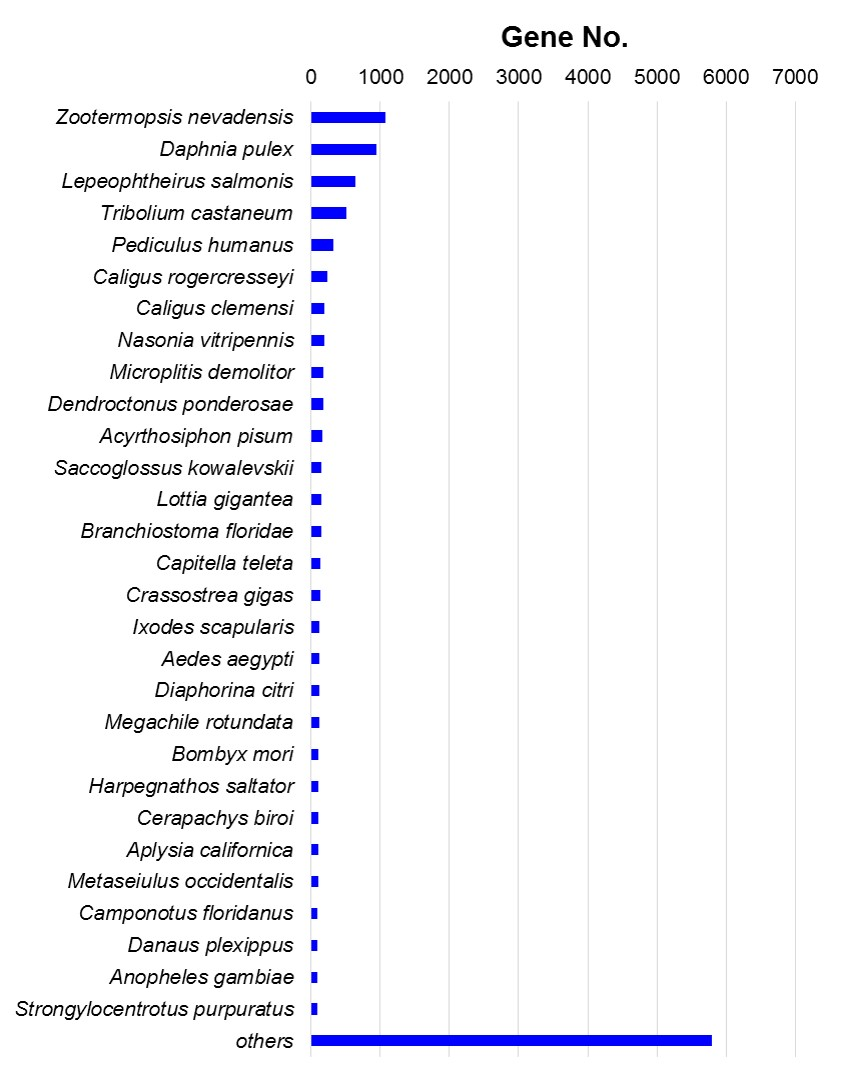

Supplement: Figure S2. — BLAST top-hit species distribution of Tigriopus kingsejongensis. Data obtained using BLASTx against the National Center for Biotechnology Information's (NCBI) non-redundant protein database with an E value cutoff of 1e−5. [file giw010_FigS2.png]

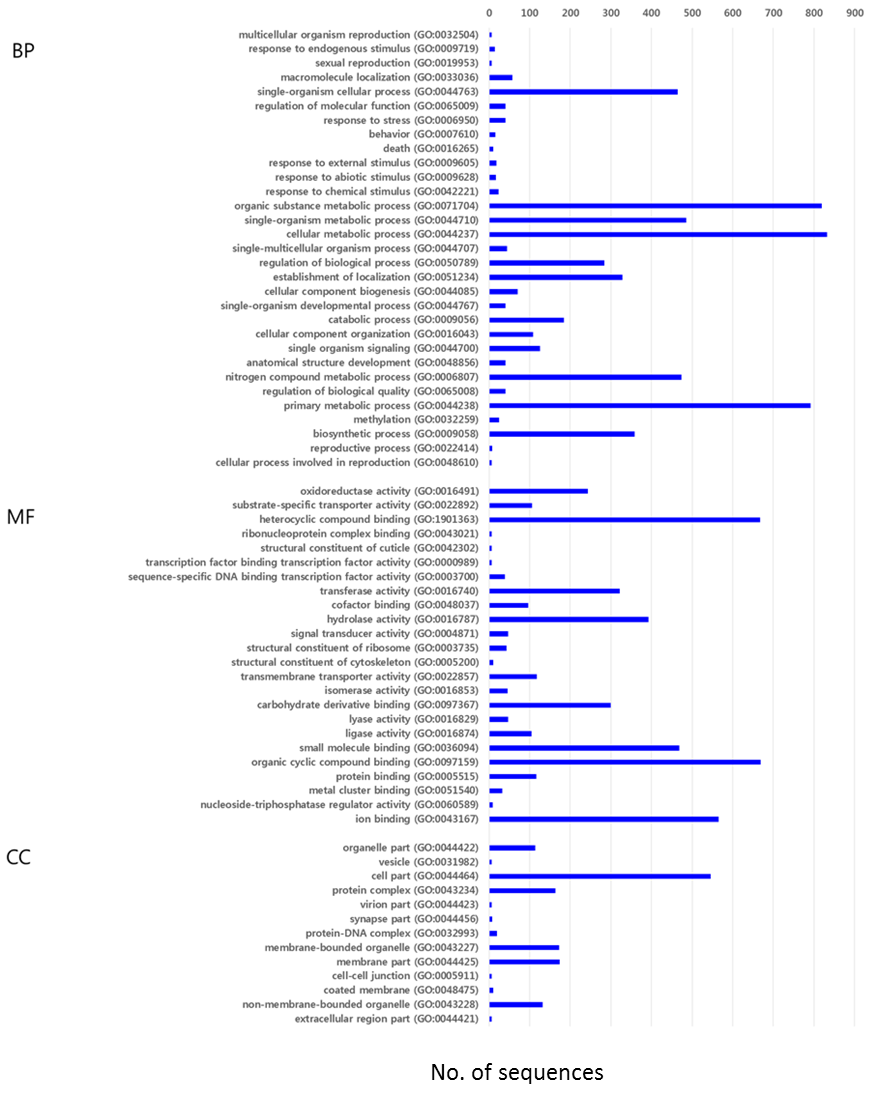

Supplement: Figure S3. — Gene Ontology distribution of annotated genes. Gene Ontology (GO) annotation of predicted Tigriopus kingsejongensis genes was conducted using the GO annotation. The figure illustrates the number of genes from major GO modules of molecular function (MF), biological process (BP), and cellular component (CC). [file giw010_FigS3.png]

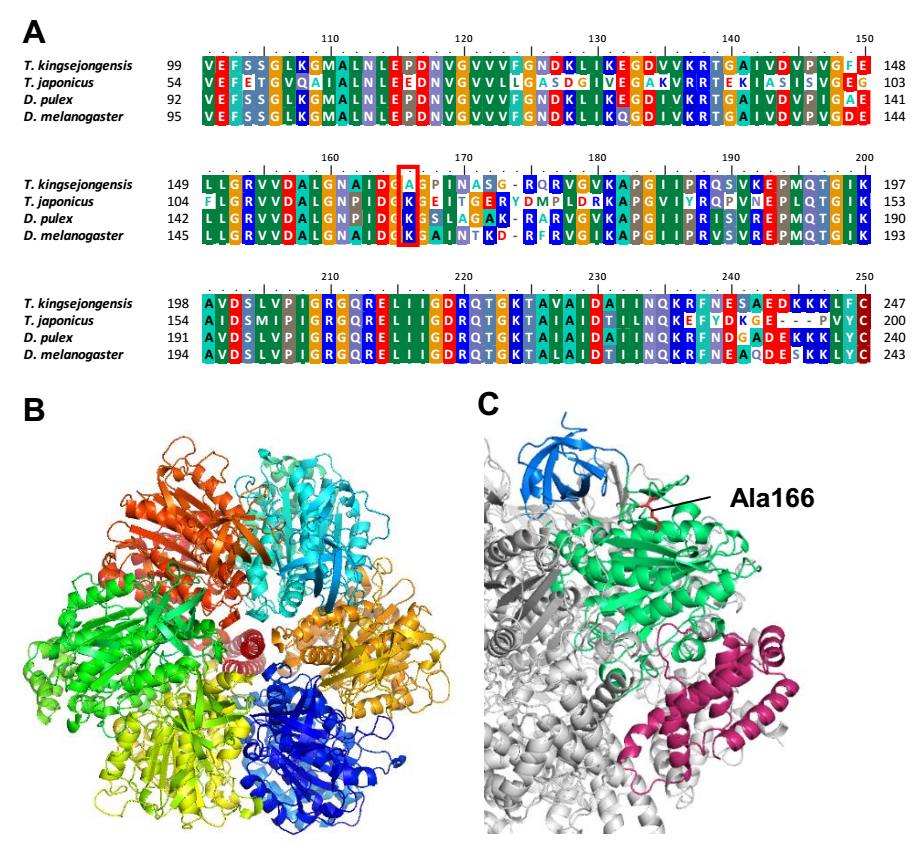

Supplement: Figure S4. — Tigriopus kingsejongensis-specific amino acid changes in ATP synthase subunit alpha. A. Clustal X alignment of the amino acid sequences between four species. Tigriopus kingsejongensis-specific amino acid changes representing positive selections are presented with red boxes. B. Cartoon of the protein crystal structure of the ATP synthase (PDB ID: 1BMF). C. The specific amino acid change Ala166 is colored in red (in stick form) and positioned within the external loop region of nucleotide-binding domain. The three domains of the ATP synthase subunit alpha illustrated in cartoon form are colored accordingly (blue, beta-barrel domain; green, nucleotide-binding domain; purple: C terminal domain). [file giw010_FigS4.png]

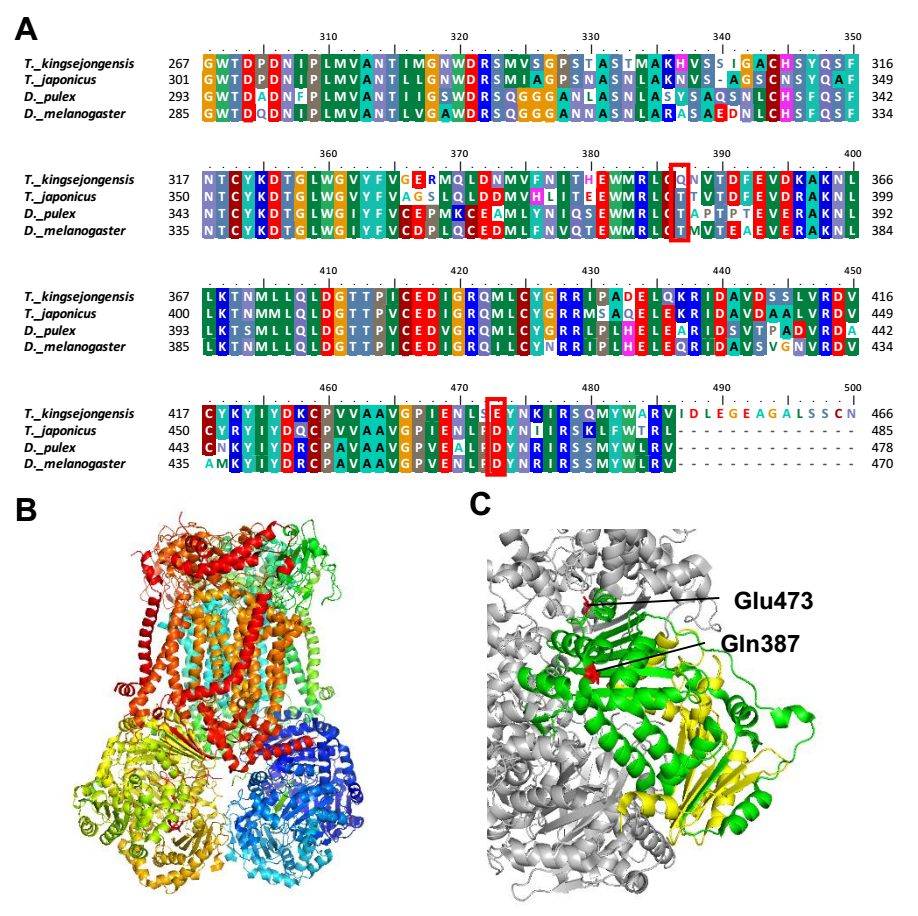

Supplement: Figure S5. — Tigriopus kingsejongensis-specific amino acid changes in ubiquinol-cytochrome c reductase core protein I. A. Clustal X alignment of the amino acid sequences between four species. Tigriopus kingsejongensis-specific amino acid changes representing positive selections are presented with red boxes. B. Cartoon of the protein crystal structure of ubiquinol-cytochrome c reductase (PDB ID: 1QCR). C. Positions of the specific amino acid changes in ubiquinol-cytochrome c reductase core protein I are colored red (stick form). The insulinase domain is yellow and the peptidase M16 domain is green. [file giw010_FigS5.png]

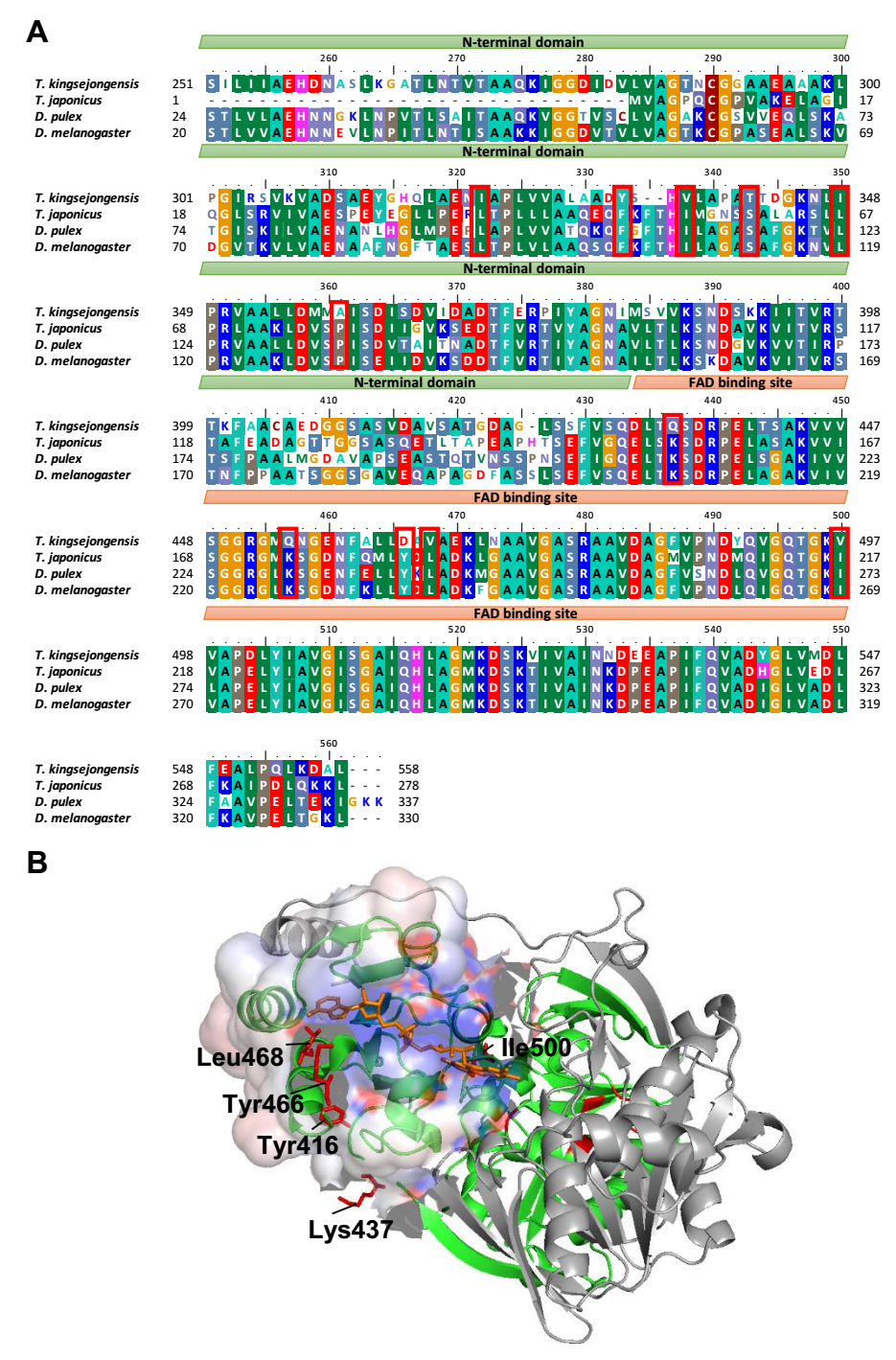

Supplement: Figure S6. — Tigriopus kingsejongensis-specific amino acid changes in electron-transferring flavoprotein. A. Clustal X alignment of the amino acid sequences between four species. Tigriopus kingsejongensis-specific amino acid changes representing positive selections are presented with red boxes. Among the ten amino acid changes, the five sites are located within the N-terminal domain and the other five are positioned within the FAD binding domain. B. Cartoon of the protein crystal structure of the electron-transferring flavoprotein (PDB ID: 1EFV). The five amino acid sites within the FAD binding domain are colored in red (stick form). Electron-transferring flavoprotein alpha subunit is green; FAD-binding domain is represented by color-coded electrostatic surface (blue, positive charge; red, negative charge; grey, neutral charge); FAD is orange (stick form). Notably, the Asp463 residue makes a salt bridge with Arg437 in the homology model structure of electron-transferring flavoprotein from T. kingsejongensis. In addition, Gln454 is located near the bound FAD co-factor and may form a hydrogen bond with the N7A atom of FAD in the model structure of electron-transferring flavoprotein from T. kingsejongensis. [file giw010_FigS6.png]
