# Supplementary material for: The genome of the Antarctic-endemic copepod, Tigriopus kingsejongensis
Source: Gigascience. 2017 Jan 7;6(1):1–9. doi: 10.1093/gigascience/giw010 (PMC5467011; doi:10.1093/gigascience/giw010)
Supplement: Table S1. — Number of tRNA in the Tigriopus kingsejongensis genome. [file giw010_TableS1.docx]

Table S1.

| **tRNA** | **Number** |
| --- | --- |
| Ala | 59 |
| Gly | 80 |
| Pro | 68 |
| Thr | 81 |
| Val | 49 |
| Ser | 120 |
| Arg | 129 |
| Leu | 173 |
| Phe | 30 |
| Asn | 33 |
| Lys | 31 |
| Asp | 30 |
| Glu | 31 |
| His | 40 |
| Gln | 32 |
| Ile | 33 |
| Met | 122 |
| Tyr | 31 |
| Cys | 38 |
| Trp | 43 |
| SelCys | 4 |
| Pseudo | 102 |
| Sum | 1359 |
