# Supplementary material for: The genome of the Antarctic-endemic copepod, Tigriopus kingsejongensis
Source: Gigascience. 2017 Jan 7;6(1):1–9. doi: 10.1093/gigascience/giw010 (PMC5467011; doi:10.1093/gigascience/giw010)
Supplement: Table S2. — Known repetitive and transposable elements in the Tigropus kingsejongensis genome. [file giw010_TableS2.docx]

Table S2.

|  |  |  | ***de novo* repeat** | |
| --- | --- | --- | --- | --- |
|  | **Transposable elements** | **Count** | **Length** | **%** |
| Retrotransposon | LTR^1^ | 12 380 | 3 755 033 | 1.319 |
|  | LINE^2^ | 4216 | 1 167 322 | 0.410 |
|  | SINE^3^ | 82 | 23 372 | 0.008 |
| Unknown | | 78 | 21 362 | 0.008 |
| Tandem repeat | Satellites | 231 | 92 355 | 0.032 |
|  | Simple repeats | 28 271 | 1 201 410 | 0.422 |
| Low complexity | | 362 | 73 979 | 0.026 |
| Total | | 45 620 | 6 334 833 | 2.225 |

^1^LTR : long terminal repeat

^2^LINE : long interspersed nuclear element3

^3^SINE : short interspersed nuclear element
