# Supplementary material for: The genome of the Antarctic-endemic copepod, Tigriopus kingsejongensis
Source: Gigascience. 2017 Jan 7;6(1):1–9. doi: 10.1093/gigascience/giw010 (PMC5467011; doi:10.1093/gigascience/giw010)
Supplement: Table S3. — Transposable elements in the Tigriopus kingsejongensis genome. [file giw010_TableS3.docx]

Table S3.

| **Transposable element** | **Gene number** | **Size** | **Proportion** |
| --- | --- | --- | --- |
| Gypsy | 7715 | 2 414 988 | 37.24 |
| Copia | 1797 | 509 597 | 7.86 |
| Pao | 1620 | 452 974 | 6.99 |
| TcMar-Tc1 | 1401 | 464 057 | 7.16 |
| L2 | 1259 | 352 146 | 5.43 |
| L1 | 533 | 148 519 | 2.29 |
| CR1 | 530 | 185 786 | 2.87 |
| CMC-EnSpm | 475 | 120 729 | 1.86 |
| ERV1 | 450 | 141 683 | 2.19 |
| LOA | 387 | 98 912 | 1.53 |
| Helitron | 341 | 91 268 | 1.41 |
| Jockey | 340 | 89 545 | 1.38 |
| DNA | 298 | 77 542 | 1.19 |
| MULE-MuDR | 265 | 65 695 | 1.01 |
| ERVK | 259 | 73 850 | 1.14 |
| R1 | 247 | 67 440 | 1.04 |
| Maverick | 228 | 64 374 | 0.99 |
| Satellite | 216 | 87 989 | 1.36 |
| hAT-Ac | 185 | 48 009 | 0.74 |
| PIF-Harbinger | 176 | 42 487 | 0.66 |
| Tad1 | 176 | 40 086 | 0.62 |
| LTR | 162 | 43 962 | 0.67 |
| TcMar-Mariner | 156 | 43 475 | 0.67 |
| CR1-Zenon | 152 | 38 743 | 0.60 |
| DIRS | 133 | 31 491 | 0.49 |
| I | 129 | 30 355 | 0.47 |
| Sola | 108 | 27 379 | 0.42 |
| RTE-X | 103 | 25 142 | 0.39 |
| L1-Tx1 | 101 | 24 236 | 0.37 |
| hAT-Charlie | 94 | 25 358 | 0.39 |
| hAT | 110 | 28 351 | 0.44 |
| P | 77 | 20 609 | 0.32 |
| Unknown | 76 | 20 912 | 0.32 |
| hAT-Tip100 | 75 | 17 585 | 0.27 |
| CMC-Chapaev-3 | 72 | 27 895 | 0.43 |
| RTE-BovB | 68 | 16 114 | 0.25 |
| hAT-hATx | 66 | 17 953 | 0.28 |
| ERVL | 62 | 48 626 | 0.75 |
| Ngaro | 60 | 15 696 | 0.24 |
| Academ | 57 | 13 580 | 0.21 |
| hAT-hATm | 55 | 13 386 | 0.21 |
| Ginger | 54 | 13 712 | 0.21 |
| Penelope | 52 | 12 697 | 0.20 |
| Crypton | 49 | 11 754 | 0.18 |
| PiggyBac | 49 | 11 624 | 0.18 |
| R2 | 48 | 12 411 | 0.19 |
| MIR | 44 | 10 394 | 0.16 |
| TcMar-Fot1 | 44 | 10 977 | 0.17 |
| hAT-Tag1 | 42 | 9909 | 0.15 |
| CMC-Chapaev | 41 | 10 232 | 0.16 |
| ERV4 | 40 | 9 561 | 0.15 |
| CMC-Transib | 37 | 8706 | 0.13 |
| Dada | 36 | 9207 | 0.14 |
| Kolobok-Hydra | 35 | 8693 | 0.13 |
| ARTEFACT | 29 | 14 145 | 0.22 |
| Novosib | 27 | 15 469 | 0.24 |
| PIF-ISL2EU | 27 | 6893 | 0.11 |
| hAT-hobo | 26 | 3052 | 0.05 |
| TcMar-Tigger | 26 | 6945 | 0.11 |
| hAT-Blackjack | 25 | 6420 | 0.10 |
| Caulimovirus | 24 | 5633 | 0.09 |
| ID | 24 | 6227 | 0.10 |
| snRNA | 24 | 16 868 | 0.26 |
| Merlin | 20 | 5229 | 0.08 |
| Rex-Babar | 19 | 4 795 | 0.07 |
| MULE-NOF | 18 | 4255 | 0.07 |
| Zator | 18 | 4232 | 0.07 |
| IS3EU | 15 | 4191 | 0.06 |
| TcMar-Tc2 | 15 | 3603 | 0.06 |
| TcMar-Sagan | 14 | 3253 | 0.05 |
| hAT-hATw | 12 | 2858 | 0.04 |
| TcMar-ISRm11 | 12 | 2829 | 0.04 |
| ERVL-MaLR | 11 | 2615 | 0.04 |
| Harbinger | 11 | 2582 | 0.04 |
| R2-Hero | 11 | 2572 | 0.04 |
| RTE | 11 | 2898 | 0.04 |
| Kolobok-T2 | 10 | 2322 | 0.04 |
| TcMar-Pogo | 10 | 2313 | 0.04 |
| Dong-R4 | 9 | 2088 | 0.03 |
| hAT-Pegasus | 9 | 2120 | 0.03 |
| TcMar-Ant1 | 9 | 2091 | 0.03 |
| 7SL | 8 | 5317 | 0.08 |
| Ambal | 8 | 1962 | 0.03 |
| TcMar | 8 | 711 | 0.01 |
| Proto2 | 7 | 1615 | 0.02 |
| Satellite/5S | 7 | 2187 | 0.03 |
| Retroposon | 6 | 1906 | 0.03 |
| RTE-RTE | 6 | 1425 | 0.02 |
| DRE | 5 | 1195 | 0.02 |
| P-Fungi | 5 | 1183 | 0.02 |
| Proto1 | 5 | 1148 | 0.02 |
| TcMar-m44 | 5 | 1217 | 0.02 |
| TcMar-Stowaway | 5 | 1179 | 0.02 |
| CRE | 4 | 1008 | 0.02 |
| MULE-F | 4 | 926 | 0.01 |
| Other | 4 | 951 | 0.01 |
| RNA | 4 | 2737 | 0.04 |
| hAT-hAT5 | 3 | 701 | 0.01 |
| R2-NeSL | 3 | 691 | 0.01 |
| Satellite/telo | 3 | 761 | 0.01 |
| hAT-hAT1 | 2 | 475 | 0.01 |
| Satellite/macro | 2 | 522 | 0.01 |
| Satellite/W-chromosome | 2 | 639 | 0.01 |
| SINE | 2 | 498 | 0.01 |
| srpRNA | 2 | 1128 | 0.02 |
| TcMar-Tc4 | 2 | 466 | 0.01 |
| Unknown/centromeric | 2 | 450 | 0.01 |
| Zorro | 2 | 475 | 0.01 |
| Alu | 1 | 228 | 0.00 |
| B2 | 1 | 250 | 0.00 |
| B4 | 1 | 231 | 0.00 |
| CMC-Mirage | 1 | 227 | 0.00 |
| Copia(Xen1) | 1 | 225 | 0.00 |
| ERV-Foamy | 1 | 229 | 0.00 |
| Gypsy-Troyka | 1 | 240 | 0.00 |
| hAT-hAT6 | 1 | 234 | 0.00 |
| hAT-Restless | 1 | 231 | 0.00 |
| Kolobok | 1 | 230 | 0.00 |
| LINE | 1 | 236 | 0.00 |
| Mermaid | 1 | 227 | 0.00 |
| Retroposon/SVA | 1 | 225 | 0.00 |
| Satellite/Y-chromosome | 1 | 257 | 0.00 |
| scRNA | 1 | 227 | 0.00 |
| TATE | 1 | 235 | 0.00 |
| TcMar-Mogwai | 1 | 234 | 0.00 |
| Sum | 21 984 | 6 484 338 |  |
