# Supplementary material for: The genome of the Antarctic-endemic copepod, Tigriopus kingsejongensis
Source: Gigascience. 2017 Jan 7;6(1):1–9. doi: 10.1093/gigascience/giw010 (PMC5467011; doi:10.1093/gigascience/giw010)
Supplement: Table S4. — Gene Ontology (GO) of lineage-specific gene families in the Tigriopus kingsejongensis genome. REVIGO software was used to cluster related GO terms (in bold letters) according to P-value. [file giw010_TableS4.docx]

Table S4.

| **GO-ID** | **Term** | **No. of genes** | **Category** | ***P*-value** |
| --- | --- | --- | --- | --- |
| **GO:0044765** | **single-organism transport** | 25 | P | 3.7E-03 |
| GO:0055085 | transmembrane transport | 16 | P | 1.9E-02 |
| GO:0006811 | ion transport | 16 | P | 3.8E-02 |
| GO:0006812 | cation transport | 12 | P | 1.1E-02 |
| **GO:0016887** | **ATPase activity** | 10 | F | 1.1E-02 |
| GO:0022804 | active transmembrane transporter activity | 9 | F | 1.8E-03 |
| GO:0042626 | ATPase activity, coupled to transmembrane movement of substances | 8 | F | 1.7E-03 |
| GO:0019829 | cation-transporting ATPase activity | 4 | F | 3.6E-02 |
| GO:0015405 | P-P-bond-hydrolysis-driven transmembrane transporter activity | 8 | F | 1.7E-03 |
| GO:0016820 | hydrolase activity, acting on acid anhydrides, catalyzing transmembrane movement of substances | 8 | F | 2.4E-03 |
| GO:0042623 | ATPase activity, coupled | 8 | F | 1.4E-02 |
| GO:0015399 | primary active transmembrane transporter activity | 8 | F | 1.7E-03 |
| GO:0043492 | ATPase activity, coupled to movement of substances | 8 | F | 1.7E-03 |
| **GO:0009064** | **glutamine family amino acid metabolic process** | 5 | P | 6.0E-03 |
| GO:0006547 | histidine metabolic process | 2 | P | 4.8E-02 |
| GO:0009084 | glutamine family amino acid biosynthetic process | 3 | P | 2.6E-02 |
| GO:0043650 | dicarboxylic acid biosynthetic process | 2 | P | 3.3E-02 |
| GO:0006537 | glutamate biosynthetic process | 2 | P | 2.1E-02 |
| GO:0006536 | glutamate metabolic process | 3 | P | 6.7E-03 |
| **GO:0042773** | **ATP synthesis coupled electron transport** | 2 | P | 4.8E-02 |
| GO:0009123 | nucleoside monophosphate metabolic process | 9 | P | 4.6E-02 |
| GO:0009161 | ribonucleoside monophosphate metabolic process | 9 | P | 4.4E-02 |
| **GO:0016841** | **ammonia-lyase activity** | 2 | F | 2.1E-02 |
| GO:0016840 | carbon-nitrogen lyase activity | 2 | F | 4.8E-02 |
| **GO:0044710** | **single-organism metabolic process** | 44 | P | 3.9E-03 |
| **GO:0016491** | **oxidoreductase activity** | 24 | F | 1.6E-02 |
| **GO:0032991** | **macromolecular complex** | 6 | C | 1.4E-02 |
| **GO:0043648** | **dicarboxylic acid metabolic process** | 3 | P | 3.3E-02 |
| **GO:0030145** | **manganese ion binding** | 2 | F | 3.3E-02 |
| **GO:0008410** | **CoA-transferase activity** | 2 | F | 4.8E-02 |
| **GO:0015930** | **glutamate synthase activity** | 2 | F | 1.1E-02 |
| **GO:0031032** | **actomyosin structure organization** | 2 | P | 3.3E-02 |
| **GO:0052803** | **imidazole-containing compound metabolic process** | 2 | P | 4.8E-02 |

F: molecular function; P: biological process; C: cellular component
