# Supplementary material for: The genome of the Antarctic-endemic copepod, Tigriopus kingsejongensis
Source: Gigascience. 2017 Jan 7;6(1):1–9. doi: 10.1093/gigascience/giw010 (PMC5467011; doi:10.1093/gigascience/giw010)
Supplement: Table S5. — Annotated domains of lineage-specific gene families in the Tigriopus kingsejongensis genome. [file giw010_TableS5.docx]

Table S5.

| **InterProScan ID** | **Number** | **Description** | **Classification** |
| --- | --- | --- | --- |
| **transporters** |  |  |  |
| IPR020846 | 15 | Major facilitator superfamily domain | DOMAIN |
| IPR005828 | 7 | Major facilitator, sugar transporter-like | DOMAIN |
| IPR003439 | 5 | ABC transporter-like | DOMAIN |
| IPR011701 | 6 | Major facilitator superfamily | DOMAIN |
| **ATPase** |  |  |  |
| IPR027417 | 35 | P-loop containing nucleoside triphosphate hydrolase | DOMAIN |
| IPR003593 | 7 | AAA+ ATPase domain | FAMILY |
| **Zinc finger** |  |  |  |
| IPR013087 | 10 | Zinc finger C2H2-type/integrase DNA-binding domain | CONSERVED_SITE |
| IPR007087 | 18 | Zinc finger, C2H2 | DOMAIN |
| IPR015880 | 16 | Zinc finger, C2H2-like | FAMILY |
| IPR001781 | 5 | Zinc finger, LIM-type | FAMILY |
| **homeodomain** |  |  |  |
| IPR009057 | 10 | Homeodomain-like | CONSERVED_SITE |
| IPR001356 | 9 | Homeobox domain | CONSERVED_SITE |
| IPR017970 | 6 | Homeobox, conserved site | DOMAIN |
| **Etc.** |  |  |  |
| IPR016040 | 12 | NAD(P)-binding domain | DOMAIN |
| IPR000618 | 10 | Insect cuticle protein | CONSERVED_SITE |
| IPR011009 | 8 | Protein kinase-like domain | DOMAIN |
| IPR000276 | 7 | G protein-coupled receptor, rhodopsin-like | DOMAIN |
| IPR002198 | 7 | Short-chain dehydrogenase/reductase SDR | DOMAIN |
| IPR002347 | 7 | Glucose/ribitol dehydrogenase | null |
| IPR012337 | 7 | Ribonuclease H-like domain | DOMAIN |
| IPR013783 | 7 | Immunoglobulin-like fold | CONSERVED_SITE |
| IPR017452 | 7 | GPCR, rhodopsin-like, 7TM | DOMAIN |
| IPR029058 | 7 | Alpha/Beta hydrolase fold | DOMAIN |
| IPR000719 | 6 | Protein kinase domain | DOMAIN |
| IPR002290 | 6 | Serine/threonine/dual specificity protein kinase, catalytic domain | DOMAIN |
| IPR002557 | 6 | Chitin binding domain | DOMAIN |
| IPR005225 | 6 | Small GTP-binding protein domain | DOMAIN |
| IPR008271 | 6 | Serine/threonine-protein kinase, active site | DOMAIN |
| IPR011992 | 6 | EF-hand domain pair | DOMAIN |
| IPR013785 | 6 | Aldolase-type TIM barrel | DOMAIN |
| IPR000210 | 5 | BTB/POZ domain | FAMILY |
| IPR001254 | 5 | Serine proteases, trypsin domain | DOMAIN |
| IPR001873 | 5 | Na+ channel, amiloride-sensitive | FAMILY |
| IPR002048 | 5 | EF-hand domain | DOMAIN |
| IPR006091 | 5 | Acyl-CoA oxidase/dehydrogenase, central domain | FAMILY |
| IPR006201 | 5 | Neurotransmitter-gated ion-channel | DOMAIN |
| IPR006202 | 5 | Neurotransmitter-gated ion-channel ligand-binding domain | FAMILY |
| IPR009003 | 5 | Peptidase S1, PA clan | DOMAIN |
| IPR009075 | 5 | Acyl-CoA dehydrogenase/oxidase C-terminal | FAMILY |
| IPR009100 | 5 | Acyl-CoA dehydrogenase/oxidase, N-terminal and middle domain | DOMAIN |
| IPR012336 | 5 | Thioredoxin-like fold | DOMAIN |
| IPR013320 | 5 | Concanavalin A-like lectin/glucanase domain | DOMAIN |
| IPR013781 | 5 | Glycoside hydrolase, catalytic domain | DOMAIN |
| IPR015421 | 5 | Pyridoxal phosphate-dependent transferase, major region, subdomain 1 | DOMAIN |
| IPR015424 | 5 | Pyridoxal phosphate-dependent transferase | DOMAIN |
| IPR016024 | 5 | Armadillo-type fold | DOMAIN |
| IPR018114 | 5 | Peptidase S1, trypsin family, active site | DOMAIN |
| IPR018247 | 5 | EF-Hand 1, calcium-binding site | DOMAIN |
