# Supplementary material for: The genome of the Antarctic-endemic copepod, Tigriopus kingsejongensis
Source: Gigascience. 2017 Jan 7;6(1):1–9. doi: 10.1093/gigascience/giw010 (PMC5467011; doi:10.1093/gigascience/giw010)
Supplement: Table S6. — Gene Ontology (GO) of expanded gene families in the Tigriopus kingsejongensis genome. REVIGO software was used to cluster related GO terms (in bold letters) according to p-value. [file giw010_TableS6.docx]

Table S6.

| **GO ID** | **Term** | **No. of genes** | **Category** | ***P-*value** |
| --- | --- | --- | --- | --- |
| **GO:0046394** | **carboxylic acid biosynthetic process** | 11 | P | 1.1E-04 |
| GO:0016053 | organic acid biosynthetic process | 11 | P | 1.1E-04 |
| GO:0043436 | oxoacid metabolic process | 21 | P | 2.0E-04 |
| GO:0032787 | monocarboxylic acid metabolic process | 8 | P | 1.3E-04 |
| GO:0019752 | carboxylic acid metabolic process | 21 | P | 2.0E-04 |
| GO:0044283 | small molecule biosynthetic process | 14 | P | 1.6E-06 |
| **GO:0045333** | **cellular respiration** | 10 | P | 3.4E-05 |
| GO:0022900 | electron transport chain | 6 | P | 1.9E-04 |
| GO:0022904 | respiratory electron transport chain | 5 | P | 2.9E-04 |
| GO:0015980 | energy derivation by oxidation of organic compounds | 10 | P | 8.1E-05 |
| **GO:0016620** | **oxidoreductase activity, acting on the aldehyde or oxo group of donors, NAD or NADP as acceptor** | 8 | F | 9.2E-05 |
| GO:0008746 | NAD(P)+ transhydrogenase activity | 4 | F | 3.0E-04 |
| **GO:0004494** | **methylmalonyl-CoA mutase activity** | 5 | F | 1.5E-06 |
| GO:0016866 | intramolecular transferase activity | 5 | F | 2.9E-05 |
| **GO:0016652** | **oxidoreductase activity, acting on NAD(P)H, NAD(P) as acceptor** | 4 | F | 3.0E-04 |
| GO:0009013 | succinate-semialdehyde dehydrogenase [NAD(P)+] activity | 3 | F | 3.3E-04 |
| **GO:0003824** | **catalytic activity** | 110 | F | 9.7E-06 |
| **GO:0008152** | **metabolic process** | 108 | P | 9.4E-05 |
| **GO:0044710** | **single-organism metabolic process** | 56 | P | 1.7E-05 |
| **GO:0016491** | **oxidoreductase activity** | 31 | F | 4.0E-04 |
| **GO:0055114** | **oxidation-reduction process** | 28 | P | 9.3E-05 |
| **GO:0006082** | **organic acid metabolic process** | 21 | P | 2.0E-04 |
| **GO:0044711** | **single-organism biosynthetic process** | 14 | P | 2.1E-06 |
| **GO:0016903** | **oxidoreductase activity, acting on the aldehyde or oxo group of donors** | 11 | F | 6.7E-06 |
| **GO:0019842** | **vitamin binding** | 10 | F | 1.2E-10 |
| **GO:0031419** | **cobalamin binding** | 8 | F | 4.6E-10 |
| **GO:0046906** | **tetrapyrrole binding** | 8 | F | 1.8E-05 |
| **GO:0051539** | **4 iron, 4 sulfur cluster binding** | 7 | F | 1.7E-04 |
| **GO:0044272** | **sulfur compound biosynthetic process** | 6 | P | 3.6E-05 |
| **GO:0006790** | **sulfur compound metabolic process** | 6 | P | 1.9E-04 |
| **GO:0008172** | **S-methyltransferase activity** | 4 | F | 2.3E-05 |
| **GO:0003994** | **aconitate hydratase activity** | 4 | F | 1.1E-04 |
| **GO:0008705** | **methionine synthase activity** | 3 | F | 3.3E-04 |
| **GO:0042084** | **5-methyltetrahydrofolate-dependent methyltransferase activity** | 3 | F | 3.3E-04 |

F: molecular function; P: biological process; C: cellular component
