# Supplementary material for: The genome of the Antarctic-endemic copepod, Tigriopus kingsejongensis
Source: Gigascience. 2017 Jan 7;6(1):1–9. doi: 10.1093/gigascience/giw010 (PMC5467011; doi:10.1093/gigascience/giw010)
Supplement: Table S7. — Gene annotation of the expanded genes in the Tigriopus kingsejongensis genome. [file giw010_TableS7.docx]

Table S7.

| **Gene ID** | **Description** | ***T. kingsejongensis*** | | ***A. aegypti*** | | ***D. melanogaster*** | | ***I. scapularis*** | | ***M. martensii*** | | ***S. maritima*** | | ***T. urticae*** | | ***D. pulex*** | |
| --- | --- | --- | --- | --- | --- | --- | --- | --- | --- | --- | --- | --- | --- | --- | --- | --- | --- |
| Tk01365 | myosin heavy muscle isoform x29 | 16 | 3 | | 1 | | 1 | | 5 | | 2 | | 3 | | 2 | |  |
| Tk00295 | cuticle protein | 13 | 0 | | 0 | | 1 | | 0 | | 4 | | 0 | | 0 | |  |
| Tk00851 | methylmalonyl- mutase | 11 | 0 | | 0 | | 1 | | 1 | | 1 | | 0 | | 1 | |  |
| Tk00078 | succinate-semialdehyde dehydrogenase | 9 | 1 | | 1 | | 1 | | 0 | | 1 | | 1 | | 1 | |  |
| Tk00623 | GL12416 | 9 | 2 | | 4 | | 5 | | 5 | | 4 | | 2 | | 7 | |  |
| Tk01101 | methionine synthase | 8 | 0 | | 0 | | 0 | | 1 | | 1 | | 0 | | 1 | |  |
| Tk02209 | udp-glucose 6-dehydrogenase | 8 | 0 | | 0 | | 1 | | 0 | | 0 | | 0 | | 1 | |  |
| Tk00033 | lipoyl synthase | 7 | 1 | | 1 | | 2 | | 1 | | 0 | | 1 | | 1 | |  |
| Tk01209 | methylmalonyl-carboxyltransferase | 7 | 0 | | 0 | | 1 | | 0 | | 1 | | 0 | | 1 | |  |
| Tk01292 | trypsin partial | 7 | 2 | | 1 | | 0 | | 0 | | 0 | | 0 | | 3 | |  |
| Tk04367 | slit homolog 3 protein | 7 | 4 | | 4 | | 2 | | 4 | | 1 | | 2 | | 3 | |  |
| Tk00185 | Long-chain-fatty-acid--CoA ligase, putative | 6 | 1 | | 1 | | 2 | | 2 | | 1 | | 2 | | 3 | |  |
| Tk00642 | sodium bicarbonate cotransporter | 6 | 2 | | 1 | | 1 | | 2 | | 2 | | 1 | | 2 | |  |
| Tk00863 | aconitase, putative | 6 | 1 | | 2 | | 2 | | 1 | | 1 | | 1 | | 1 | |  |
| Tk01051 | alpha-ketoglutarate partial | 6 | 2 | | 2 | | 1 | | 0 | | 1 | | 1 | | 1 | |  |
| Tk01143 | 5-oxoprolinase | 6 | 1 | | 1 | | 1 | | 1 | | 1 | | 1 | | 2 | |  |
| Tk03086 | voltage-dependent calcium channel type a subunit alpha-1 isoform x7 | 6 | 3 | | 2 | | 1 | | 3 | | 3 | | 3 | | 2 | |  |
| Tk00023 | glutaminyl-tRNA synthetase | 5 | 0 | | 1 | | 1 | | 1 | | 1 | | 1 | | 1 | |  |
| Tk00025 | lon protease | 5 | 1 | | 1 | | 1 | | 0 | | 1 | | 2 | | 2 | |  |
| Tk00044 | fumarate reductase | 5 | 1 | | 2 | | 2 | | 1 | | 1 | | 1 | | 3 | |  |
| Tk00116 | acetyl-synthetase | 5 | 2 | | 1 | | 1 | | 1 | | 2 | | 1 | | 2 | |  |
| Tk01808 | fibroblast growth factor receptor-like 1 isoform x2 | 5 | 0 | | 0 | | 0 | | 0 | | 1 | | 1 | | 1 | |  |
| Tk02003 | NAD transhydrogenase subunit alpha | 5 | 1 | | 0 | | 1 | | 2 | | 1 | | 0 | | 1 | |  |
| Tk02623 | solute carrier family 22 member 15-like | 5 | 0 | | 0 | | 0 | | 0 | | 1 | | 0 | | 5 | |  |
| Tk02753 | hypothetical protein CAPTEDRAFT_228436 | 5 | 0 | | 0 | | 0 | | 0 | | 1 | | 0 | | 0 | |  |
| Tk03767 | ATP-dependent DNA helicase | 5 | 1 | | 1 | | 1 | | 0 | | 1 | | 1 | | 1 | |  |
| Tk04893 | glutamate synthase subunit alpha | 5 | 0 | | 0 | | 1 | | 1 | | 3 | | 1 | | 1 | |  |
| Tk04907 | methylmalonate-semialdehyde dehydrogenase | 5 | 1 | | 1 | | 1 | | 2 | | 1 | | 1 | | 1 | |  |
| Tk05235 | tRNA 2-thiouridylase | 5 | 4 | | 1 | | 1 | | 0 | | 1 | | 0 | | 1 | |  |
| Tk06211 | tyrosine-protein phosphatase 99a | 5 | 1 | | 1 | | 1 | | 2 | | 1 | | 1 | | 1 | |  |
| Tk09475 | hypothetical protein DAPPUDRAFT_306563 | 5 | 1 | | 0 | | 1 | | 1 | | 0 | | 1 | | 1 | |  |
| Tk00037 | glycine dehydrogenase | 4 | 2 | | 1 | | 1 | | 1 | | 1 | | 2 | | 2 | |  |
| Tk00348 | propionyl-synthetase | 4 | 1 | | 0 | | 1 | | 0 | | 0 | | 0 | | 1 | |  |
| Tk00895 | pyruvate carboxylase | 4 | 1 | | 1 | | 0 | | 1 | | 1 | | 1 | | 2 | |  |
| Tk01301 | aconitate hydratase | 4 | 2 | | 1 | | 0 | | 2 | | 1 | | 1 | | 1 | |  |
| Tk02698 | pyruvate dehydrogenase e1 subunit alpha | 4 | 4 | | 2 | | 1 | | 1 | | 1 | | 1 | | 1 | |  |
| Tk03091 | kin of irre-like protein 2 | 4 | 0 | | 0 | | 0 | | 1 | | 0 | | 1 | | 0 | |  |
| Tk03469 | propionyl-carboxylase alpha mitochondrial | 4 | 0 | | 0 | | 1 | | 1 | | 1 | | 0 | | 1 | |  |
| Tk03588 | cytochrome b subunit of cytochrome bc1 | 4 | 1 | | 1 | | 1 | | 0 | | 0 | | 0 | | 1 | |  |
| Tk04125 | predicted protein | 4 | 0 | | 0 | | 0 | | 0 | | 0 | | 0 | | 3 | |  |
| Tk06230 | aael003425- partial | 4 | 3 | | 1 | | 1 | | 1 | | 2 | | 2 | | 2 | |  |
| Tk06886 | PREDICTED: uncharacterized protein LOC103514618 | 4 | 2 | | 1 | | 2 | | 3 | | 2 | | 2 | | 2 | |  |
| Tk08400 | hypothetical protein DAPPUDRAFT_312019 | 4 | 1 | | 1 | | 1 | | 1 | | 2 | | 1 | | 3 | |  |
| Tk09835 | hypothetical protein DAPPUDRAFT_45422 | 4 | 0 | | 0 | | 1 | | 0 | | 1 | | 5 | | 0 | |  |
| Tk11890 | hypothetical protein D910_00038 | 4 | 1 | | 1 | | 1 | | 1 | | 1 | | 0 | | 1 | |  |
| Tk00056 | cysteinyl-tRNA synthetase | 3 | 0 | | 0 | | 1 | | 2 | | 1 | | 1 | | 0 | |  |
| Tk00131 | hypothetical protein BRAFLDRAFT_127065 | 3 | 1 | | 1 | | 1 | | 3 | | 0 | | 1 | | 2 | |  |
| Tk00154 | molecular chaperone | 3 | 0 | | 1 | | 2 | | 0 | | 1 | | 1 | | 1 | |  |
| Tk01076 | charged multivesicular body protein 4c-like | 3 | 1 | | 1 | | 1 | | 0 | | 1 | | 1 | | 1 | |  |
| Tk01088 | hypothetical protein DAPPUDRAFT_306990 | 3 | 1 | | 0 | | 0 | | 0 | | 0 | | 0 | | 1 | |  |
| Tk01302 | NAD-dependent dehydratase | 3 | 1 | | 1 | | 1 | | 1 | | 1 | | 1 | | 1 | |  |
| Tk01363 | hypothetical protein DAPPUDRAFT_47227 | 3 | 1 | | 1 | | 1 | | 1 | | 1 | | 1 | | 2 | |  |
| Tk01630 | SAM domain and HD domain-containing protein 1-like | 3 | 1 | | 1 | | 1 | | 0 | | 1 | | 0 | | 0 | |  |
| Tk01791 | GTP cyclohydrolase i protein punch | 3 | 1 | | 1 | | 1 | | 1 | | 1 | | 1 | | 1 | |  |
| Tk01920 | aldehyde dehydrogenase | 3 | 0 | | 0 | | 0 | | 1 | | 1 | | 0 | | 0 | |  |
| Tk02025 | nadh-ubiquinone oxidoreductase chain l | 3 | 0 | | 0 | | 0 | | 0 | | 0 | | 0 | | 1 | |  |
| Tk02118 | mitochondrial ornithine transporter | 3 | 2 | | 1 | | 1 | | 2 | | 0 | | 1 | | 1 | |  |
| Tk02158 | hypothetical protein SPRG_16348, partial | 3 | 0 | | 0 | | 0 | | 0 | | 1 | | 0 | | 0 | |  |
| Tk02206 | paired box protein PAX-6-like | 3 | 0 | | 0 | | 0 | | 1 | | 1 | | 0 | | 0 | |  |
| Tk02297 | electron transfer flavoprotein subunit alpha | 3 | 1 | | 1 | | 1 | | 1 | | 1 | | 1 | | 1 | |  |
| Tk02454 | aldehyde oxidase 1 isoform x1 | 3 | 2 | | 1 | | 0 | | 0 | | 1 | | 0 | | 1 | |  |
| Tk02565 | integrator complex subunit 1-like | 3 | 1 | | 1 | | 1 | | 1 | | 1 | | 1 | | 2 | |  |
| Tk02676 | MFS transporter | 3 | 1 | | 1 | | 1 | | 0 | | 0 | | 0 | | 1 | |  |
| Tk03189 | groucho-like protein | 3 | 2 | | 0 | | 2 | | 2 | | 1 | | 1 | | 2 | |  |
| Tk03368 | phospholipase beta isoform | 3 | 3 | | 1 | | 1 | | 1 | | 1 | | 1 | | 2 | |  |
| Tk03378 | PREDICTED: neprilysin-2 | 3 | 1 | | 1 | | 0 | | 2 | | 1 | | 2 | | 1 | |  |
| Tk03550 | hypothetical protein Phum_PHUM025060 | 3 | 1 | | 1 | | 1 | | 1 | | 1 | | 2 | | 0 | |  |
| Tk03556 | trypsin, putative | 3 | 1 | | 1 | | 1 | | 1 | | 1 | | 1 | | 0 | |  |
| Tk03683 | GI20085 | 3 | 0 | | 0 | | 1 | | 1 | | 0 | | 2 | | 0 | |  |
| Tk03747 | tropomyosin | 3 | 0 | | 0 | | 1 | | 0 | | 0 | | 0 | | 0 | |  |
| Tk04025 | hexokinase type | 3 | 2 | | 1 | | 0 | | 1 | | 1 | | 1 | | 1 | |  |
| Tk04056 | hypothetical protein LOTGIDRAFT_190462 | 3 | 1 | | 0 | | 2 | | 0 | | 2 | | 2 | | 2 | |  |
| Tk04058 | hypothetical protein GLOINDRAFT_9379 | 3 | 0 | | 1 | | 1 | | 2 | | 2 | | 0 | | 2 | |  |
| Tk04315 | hypothetical protein DAPPUDRAFT_128092 | 3 | 0 | | 0 | | 1 | | 2 | | 1 | | 0 | | 3 | |  |
| Tk04536 | HIV-1 TAT interactive protein 30kda | 3 | 0 | | 0 | | 0 | | 0 | | 0 | | 0 | | 1 | |  |
| Tk04599 | transcription initiation protein spt3 homolog | 3 | 0 | | 0 | | 1 | | 0 | | 1 | | 1 | | 1 | |  |
| Tk04670 | guanine nucleotide-binding protein | 3 | 1 | | 1 | | 1 | | 0 | | 1 | | 1 | | 1 | |  |
| Tk05299 | monocarboxylate transporter 12-like | 3 | 1 | | 0 | | 1 | | 0 | | 1 | | 1 | | 1 | |  |
| Tk05663 | peptidoglycan recognition protein | 3 | 2 | | 2 | | 1 | | 0 | | 2 | | 0 | | 0 | |  |
| Tk05773 | octanoyltransferase | 3 | 1 | | 1 | | 1 | | 1 | | 1 | | 1 | | 1 | |  |
| Tk06070 | cell adhesion molecule 3 precursor | 3 | 1 | | 1 | | 1 | | 1 | | 2 | | 2 | | 1 | |  |
| Tk06095 | gamma-aminobutyric acid receptor alpha-like | 3 | 0 | | 0 | | 0 | | 1 | | 0 | | 0 | | 0 | |  |
| Tk06617 | pyruvate dehydrogenase | 3 | 1 | | 1 | | 2 | | 2 | | 1 | | 1 | | 1 | |  |
| Tk06811 | peroxisomal-trans-enoyl-partial | 3 | 1 | | 1 | | 2 | | 2 | | 1 | | 1 | | 3 | |  |
| Tk07608 | hypothetical protein DAPPUDRAFT_130439 | 3 | 1 | | 0 | | 1 | | 1 | | 1 | | 1 | | 1 | |  |
| Tk08286 | hypothetical protein BRAFLDRAFT_118340 | 3 | 0 | | 0 | | 0 | | 0 | | 1 | | 0 | | 1 | |  |
| Tk08432 | hypothetical protein BRAFLDRAFT_278711 | 3 | 1 | | 1 | | 2 | | 2 | | 1 | | 0 | | 1 | |  |
| Tk08908 | S-formylglutathione hydrolase | 3 | 1 | | 1 | | 1 | | 2 | | 0 | | 1 | | 1 | |  |
| Tk09328 | T-box transcription factor tbx20 | 3 | 2 | | 1 | | 1 | | 1 | | 2 | | 2 | | 2 | |  |
| Tk12475 | TPA:arylsulfatase d-like | 3 | 0 | | 0 | | 1 | | 1 | | 1 | | 1 | | 1 | |  |
