# Supplementary material for: The genome of the Antarctic-endemic copepod, Tigriopus kingsejongensis
Source: Gigascience. 2017 Jan 7;6(1):1–9. doi: 10.1093/gigascience/giw010 (PMC5467011; doi:10.1093/gigascience/giw010)
Supplement: Table S8. — Gene Ontology (GO) of contracted genes in the Tigriopus kingsejongensis genome. REVIGO software was used to cluster related GO terms (in bold letters) according to P-value. [file giw010_TableS8.docx]

Table S8.

| **GO ID** | **Term** | **No. of genes** | **Category** | ***p*-value** |
| --- | --- | --- | --- | --- |
| **GO:0009207** | **purine ribonucleoside triphosphate catabolic process** | 3 | P | 1.3E-02 |
| GO:0019439 | aromatic compound catabolic process | 3 | P | 1.7E-02 |
| GO:0006200 | ATP catabolic process | 2 | P | 2.9E-02 |
| GO:0046034 | ATP metabolic process | 2 | P | 3.4E-02 |
| GO:1901136 | carbohydrate derivative catabolic process | 3 | P | 1.4E-02 |
| GO:0044248 | cellular catabolic process | 3 | P | 3.4E-02 |
| GO:0044270 | cellular nitrogen compound catabolic process | 3 | P | 1.6E-02 |
| GO:1901658 | glycosyl compound catabolic process | 3 | P | 1.3E-02 |
| GO:0046700 | heterocycle catabolic process | 3 | P | 1.6E-02 |
| GO:0034655 | nucleobase-containing compound catabolic process | 3 | P | 1.4E-02 |
| GO:0009164 | nucleoside catabolic process | 3 | P | 1.3E-02 |
| GO:0009116 | nucleoside metabolic process | 3 | P | 2.3E-02 |
| GO:0009125 | nucleoside monophosphate catabolic process | 2 | P | 2.9E-02 |
| GO:0009123 | nucleoside monophosphate metabolic process | 2 | P | 4.9E-02 |
| GO:1901292 | nucleoside phosphate catabolic process | 3 | P | 1.4E-02 |
| GO:0006753 | nucleoside phosphate metabolic process | 3 | P | 3.5E-02 |
| GO:0009143 | nucleoside triphosphate catabolic process | 3 | P | 1.3E-02 |
| GO:0009141 | nucleoside triphosphate metabolic process | 3 | P | 1.7E-02 |
| GO:0009166 | nucleotide catabolic process | 3 | P | 1.4E-02 |
| GO:0009117 | nucleotide metabolic process | 3 | P | 3.4E-02 |
| GO:1901361 | organic cyclic compound catabolic process | 3 | P | 1.7E-02 |
| GO:1901565 | organonitrogen compound catabolic process | 3 | P | 2.1E-02 |
| GO:0046434 | organophosphate catabolic process | 3 | P | 1.4E-02 |
| GO:0006152 | purine nucleoside catabolic process | 3 | P | 1.3E-02 |
| GO:0042278 | purine nucleoside metabolic process | 3 | P | 1.8E-02 |
| GO:0009128 | purine nucleoside monophosphate catabolic process | 2 | P | 2.9E-02 |
| GO:0009126 | purine nucleoside monophosphate metabolic process | 2 | P | 4.1E-02 |
| GO:0009146 | purine nucleoside triphosphate catabolic process | 3 | P | 1.3E-02 |
| GO:0009144 | purine nucleoside triphosphate metabolic process | 3 | P | 1.5E-02 |
| GO:0006195 | purine nucleotide catabolic process | 3 | P | 1.3E-02 |
| GO:0006163 | purine nucleotide metabolic process | 3 | P | 1.9E-02 |
| GO:0046130 | purine ribonucleoside catabolic process | 3 | P | 1.3E-02 |
| GO:0046128 | purine ribonucleoside metabolic process | 3 | P | 1.8E-02 |
| GO:0009169 | purine ribonucleoside monophosphate catabolic process | 2 | P | 2.9E-02 |
| GO:0009167 | purine ribonucleoside monophosphate metabolic process | 2 | P | 4.1E-02 |
| GO:0009205 | purine ribonucleoside triphosphate metabolic process | 3 | P | 1.5E-02 |
| GO:0009154 | purine ribonucleotide catabolic process | 3 | P | 1.3E-02 |
| GO:0009150 | purine ribonucleotide metabolic process | 3 | P | 1.8E-02 |
| GO:0072523 | purine-containing compound catabolic process | 3 | P | 1.3E-02 |
| GO:0042454 | ribonucleoside catabolic process | 3 | P | 1.3E-02 |
| GO:0009119 | ribonucleoside metabolic process | 3 | P | 2.2E-02 |
| GO:0009158 | ribonucleoside monophosphate catabolic process | 2 | P | 2.9E-02 |
| GO:0009161 | ribonucleoside monophosphate metabolic process | 2 | P | 4.8E-02 |
| GO:0009203 | ribonucleoside triphosphate catabolic process | 3 | P | 1.3E-02 |
| GO:0009199 | ribonucleoside triphosphate metabolic process | 3 | P | 1.7E-02 |
| GO:0009261 | ribonucleotide catabolic process | 3 | P | 1.3E-02 |
| GO:0009259 | ribonucleotide metabolic process | 3 | P | 2.1E-02 |
| **GO:0042623** | **ATPase activity, coupled** | 2 | F | 2.6E-02 |
| GO:0016462 | pyrophosphatase activity | 3 | F | 3.4E-02 |
| GO:0016887 | ATPase activity | 2 | F | 4.6E-02 |
| GO:0016818 | hydrolase activity, acting on acid anhydrides, in phosphorus-containing anhydrides | 4 | F | 5.0E-03 |
| GO:0017111 | nucleoside-triphosphatase activity | 3 | F | 3.3E-02 |
| **GO:0006486** | **protein glycosylation** | 1 | P | 4.9E-03 |
| GO:0009101 | glycoprotein biosynthetic process | 1 | P | 4.9E-03 |
| GO:0043413 | macromolecule glycosylation | 1 | P | 4.9E-03 |
| **GO:0019693** | **ribose phosphate metabolic process** | 3 | P | 2.1E-02 |
| GO:1901657 | glycosyl compound metabolic process | 3 | P | 2.3E-02 |
| **GO:0004791** | **thioredoxin-disulfide reductase activity** | 1 | F | 4.9E-03 |
| GO:0016668 | oxidoreductase activity, acting on a sulfur group of donors, NAD(P) as acceptor | 1 | F | 2.9E-02 |
| **GO:0003918** | **DNA topoisomerase type II (ATP-hydrolyzing) activity** | 1 | F | 4.9E-03 |
| GO:0061505 | DNA topoisomerase II activity | 1 | F | 4.9E-03 |
| **GO:0004112** | **cyclic-nucleotide phosphodiesterase activity** | 1 | F | 1.5E-02 |
| GO:0004114 | 3',5'-cyclic-nucleotide phosphodiesterase activity | 1 | F | 1.5E-02 |
| **GO:0009100** | **glycoprotein metabolic process** | 1 | P | 4.9E-03 |
| **GO:0070085** | **glycosylation** | 1 | P | 4.9E-03 |
| **GO:1901135** | **carbohydrate derivative metabolic process** | 4 | P | 5.2E-03 |
| **GO:0006265** | **DNA topological change** | 1 | P | 9.7E-03 |
| **GO:0072521** | **purine-containing compound metabolic process** | 3 | P | 2.1E-02 |
| **GO:0055086** | **nucleobase-containing small molecule metabolic process** | 3 | P | 4.0E-02 |
| **GO:0008094** | **DNA-dependent ATPase activity** | 1 | F | 3.8E-02 |
| **GO:0008081** | **phosphoric diester hydrolase activity** | 1 | F | 4.3E-02 |
| **GO:0005743** | **mitochondrial inner membrane** | 1 | C | 4.8E-02 |
| **GO:0004222** | **metalloendopeptidase activity** | 1 | F | 2.4E-02 |
| **GO:0016817** | **hydrolase activity, acting on acid anhydrides** | 4 | F | 5.3E-03 |
| **GO:0016758** | **transferase activity, transferring hexosyl groups** | 1 | F | 4.3E-02 |
| **GO:0003916** | **DNA topoisomerase activity** | 1 | F | 1.5E-02 |
| **GO:0019866** | **organelle inner membrane** | 1 | C | 4.8E-02 |
| **GO:0019637** | **organophosphate metabolic process** | 3 | P | 4.5E-02 |
| **GO:0003676** | **nucleic acid binding** | 4 | F | 1.9E-02 |
| **GO:0004576** | **oligosaccharyl transferase activity** | 1 | F | 9.7E-03 |
| **GO:0016787** | **hydrolase activity** | 6 | F | 5.6E-03 |

F: molecular function; P: biological process; C: cellular component
