# Supplementary material for: The genome of the Antarctic-endemic copepod, Tigriopus kingsejongensis
Source: Gigascience. 2017 Jan 7;6(1):1–9. doi: 10.1093/gigascience/giw010 (PMC5467011; doi:10.1093/gigascience/giw010)
Supplement: Table S9. — Kyoto Encyclopedia of Genes and Genomes (KEGG) pathway of expanded genes in the Tigriopus kingsejongensis genome. [file giw010_TableS9.docx]

Table S9.

| **Pathways** | | **Number of orthologous genes** | **Sequences in Pathway** | **Enzyme** | **Enzyme Commission** |
| --- | --- | --- | --- | --- | --- |
| **Amino acid metabolism** | |  |  |  |  |
|  | Phenylalanine metabolism | 2 | 2 | transaminase | EC:2.6.1.5 |
|  | Purine metabolism | 4 | 1 | reductase | EC:1.17.4.1 |
|  | Purine metabolism | 4 | 1 | phosphoribosyltransferase | EC:2.4.2.7 |
|  | Purine metabolism | 4 | 1 | kinase | EC:2.7.4.3 |
|  | Purine metabolism | 4 | 1 | adenylpyrophosphatase | EC:3.6.1.3 |
|  | Tyrosine metabolism | 5 | 2 | transaminase | EC:2.6.1.5 |
|  | Tyrosine metabolism | 5 | 3 | dehydrogenase [NAD(P)+] | EC:1.2.1.16 |
|  | Tryptophan metabolism | 2 | 2 | dehydrogenase (succinyl-transferring) | EC:1.2.4.2 |
|  | Arginine biosynthesis | 1 | 1 | lyase | EC:4.3.2.1 |
|  | Arginine biosynthesis | 1 | 1 | synthase | EC:6.3.4.5 |
|  | Alanine, aspartate and glutamate metabolism | 4 | 1 | lyase | EC:4.3.2.1 |
|  | Alanine, aspartate and glutamate metabolism | 4 | 3 | dehydrogenase [NAD(P)+] | EC:1.2.1.16 |
|  | Alanine, aspartate and glutamate metabolism | 4 | 1 | synthase | EC:6.3.4.5 |
|  | Valine, leucine and isoleucine degradation | 8 | 2 | dehydrogenase (CoA-acylating) | EC:1.2.1.27 |
|  | Valine, leucine and isoleucine degradation | 8 | 1 | carboxylase | EC:6.4.1.3 |
|  | Valine, leucine and isoleucine degradation | 8 | 5 | mutase | EC:5.4.99.2 |
|  | Lysine degradation | 2 | 2 | dehydrogenase (succinyl-transferring) | EC:1.2.4.2 |
|  | Cysteine and methionine metabolism | 5 | 2 | transaminase | EC:2.6.1.5 |
|  | Cysteine and methionine metabolism | 5 | 3 | synthase | EC:2.1.1.13 |
|  | Cysteine and methionine metabolism | 5 | 2 | S-methyltransferase | EC:2.1.1.10 |
|  | Phenylalanine, tyrosine and tryptophan biosynthesis | 2 | 2 | transaminase | EC:2.6.1.5 |
| **Carbohydrate metabolism** | |  |  |  |  |
|  | Pyruvate metabolism | 6 | 1 | acetyltransferase | EC:2.3.1.12 |
|  | Pyruvate metabolism | 6 | 1 | ligase | EC:6.2.1.1 |
|  | Pyruvate metabolism | 6 | 1 | carboxylase | EC:6.4.1.2 |
|  | Pyruvate metabolism | 6 | 3 | carboxylase | EC:6.4.1.1 |
|  | Butanoate metabolism | 3 | 3 | dehydrogenase [NAD(P)+] | EC:1.2.1.16 |
|  | Glycolysis / Gluconeogenesis | 2 | 1 | acetyltransferase | EC:2.3.1.12 |
|  | Glycolysis / Gluconeogenesis | 2 | 1 | ligase | EC:6.2.1.1 |
|  | Pentose and glucuronate interconversions | 2 | 2 | 6-dehydrogenase | EC:1.1.1.22 |
|  | Amino sugar and nucleotide sugar metabolism | 2 | 2 | 6-dehydrogenase | EC:1.1.1.22 |
|  | Glyoxylate and dicarboxylate metabolism | 11 | 4 | hydratase | EC:4.2.1.3 |
|  | Glyoxylate and dicarboxylate metabolism | 11 | 1 | synthase (ferredoxin) | EC:1.4.7.1 |
|  | Glyoxylate and dicarboxylate metabolism | 11 | 1 | carboxylase | EC:6.4.1.3 |
|  | Glyoxylate and dicarboxylate metabolism | 11 | 5 | mutase | EC:5.4.99.2 |
|  | Ascorbate and aldarate metabolism | 2 | 2 | 6-dehydrogenase | EC:1.1.1.22 |
|  | Citrate cycle (TCA cycle) | 10 | 1 | acetyltransferase | EC:2.3.1.12 |
|  | Citrate cycle (TCA cycle) | 10 | 4 | hydratase | EC:4.2.1.3 |
|  | Citrate cycle (TCA cycle) | 10 | 2 | dehydrogenase (succinyl-transferring) | EC:1.2.4.2 |
|  | Citrate cycle (TCA cycle) | 10 | 3 | carboxylase | EC:6.4.1.1 |
|  | Starch and sucrose metabolism | 2 | 2 | 6-dehydrogenase | EC:1.1.1.22 |
| **Metabolism of cofactors and vitamins** | |  |  |  |  |
|  | Ubiquinone and other terpenoid-quinone biosynthesis | 2 | 2 | transaminase | EC:2.6.1.5 |
|  | One carbon pool by folate | 3 | 3 | synthase | EC:2.1.1.13 |
|  | Nicotinate and nicotinamide metabolism | 3 | 3 | transhydrogenase (Re/Si-specific) | EC:1.6.1.2 |
|  | Folate biosynthesis | 2 | 2 | cyclohydrolase I | EC:3.5.4.16 |
|  | Lipoic acid metabolism | 3 | 3 | synthase | EC:2.8.1.8 |
| **Energy metabolism** | |  |  |  |  |
|  | Nitrogen metabolism | 1 | 1 | synthase (ferredoxin) | EC:1.4.7.1 |
|  | Methane metabolism | 1 | 1 | ligase | EC:6.2.1.1 |
|  | Oxidative phosphorylation | 2 | 1 | oxidase | EC:1.9.3.1 |
|  | Oxidative phosphorylation | 2 | 1 | reductase (H+-translocating) | EC:1.6.5.3 |
| **Biosynthesis of other secondary metabolites** | |  |  |  |  |
|  | Tropane, piperidine and pyridine alkaloid biosynthesis | 2 | 2 | transaminase | EC:2.6.1.5 |
|  | Aflatoxin biosynthesis | 1 | 1 | carboxylase | EC:6.4.1.2 |
|  | Isoquinoline alkaloid biosynthesis | 2 | 2 | transaminase | EC:2.6.1.5 |
|  | Novobiocin biosynthesis | 2 | 2 | transaminase | EC:2.6.1.5 |
| **Others** | |  |  |  |  |
|  | Selenocompound metabolism | 3 | 3 | synthase | EC:2.1.1.13 |
|  | Glutathione metabolism | 1 | 1 | reductase | EC:1.17.4.1 |
|  | Fatty acid biosynthesis | 1 | 1 | carboxylase | EC:6.4.1.2 |
|  | Tetracycline biosynthesis | 1 | 1 | carboxylase | EC:6.4.1.2 |
|  | Pyrimidine metabolism | 1 | 1 | reductase | EC:1.17.4.1 |
