# Supplementary material for: The genome of the Antarctic-endemic copepod, Tigriopus kingsejongensis
Source: Gigascience. 2017 Jan 7;6(1):1–9. doi: 10.1093/gigascience/giw010 (PMC5467011; doi:10.1093/gigascience/giw010)
Supplement: Table S10. — Gene Ontology (GO) categories displaying w (ratio of nonsynonymous (dN) to synonymous mutations (dS)) in the genomes of Tigriopus kingsejongensis and T. japonicus. [file giw010_TableS10.docx]

Table S10.

| **GO IDs** | **GO descriptions** | **Category** | **Gene**  **count** | ***Tigriopus kingsejongensis*** | | ***Tigriopus japonicus*** | |
| --- | --- | --- | --- | --- | --- | --- | --- |
|  |  |  |  | ***w*** | **Test statistic** | ***w*** | **Test statistic** |
| GO:0031974 | membrane-enclosed lumen | C | 131 | 0.00307 | 0.00014 | 0.00183 | 0.99991 |
| GO:0070013 | intracellular organelle lumen | C | 129 | 0.00306 | 0.00015 | 0.00182 | 0.99991 |
| GO:0043233 | organelle lumen | C | 129 | 0.00306 | 0.00015 | 0.00182 | 0.99991 |
| GO:0005739 | mitochondrion | C | 155 | 0.00350 | 0.00023 | 0.00237 | 0.99985 |
| GO:0031980 | mitochondrial lumen | C | 60 | 0.00347 | 0.00127 | 0.00198 | 0.99932 |
| GO:0005759 | mitochondrial matrix | C | 60 | 0.00347 | 0.00127 | 0.00198 | 0.99932 |
| GO:0044429 | mitochondrial part | C | 116 | 0.00336 | 0.00164 | 0.00236 | 0.99894 |
| GO:0044451 | nucleoplasm part | C | 42 | 0.00244 | 0.00474 | 0.00133 | 0.99755 |
| GO:0005996 | monosaccharide metabolic process | P | 28 | 0.00316 | 0.00545 | 0.00166 | 0.99757 |
| GO:0019318 | hexose metabolic process | P | 24 | 0.00331 | 0.00607 | 0.00150 | 0.99744 |
| GO:0005811 | lipid particle | C | 86 | 0.00313 | 0.00728 | 0.00192 | 0.99529 |
| GO:0016779 | nucleotidyltransferase activity | F | 20 | 0.00411 | 0.00876 | 0.00170 | 0.99648 |
| GO:0019842 | vitamin binding | F | 28 | 0.00347 | 0.00982 | 0.00137 | 0.99537 |
| GO:0005654 | nucleoplasm | C | 46 | 0.00280 | 0.00989 | 0.00165 | 0.99446 |
| GO:0055114 | oxidation reduction | P | 108 | 0.00311 | 0.01127 | 0.00232 | 0.99219 |
| GO:0006732 | coenzyme metabolic process | P | 35 | 0.00349 | 0.01332 | 0.00233 | 0.99300 |
| GO:0051186 | cofactor metabolic process | P | 43 | 0.00344 | 0.01573 | 0.00267 | 0.99105 |
| GO:0048037 | cofactor binding | F | 66 | 0.00267 | 0.01733 | 0.00203 | 0.98890 |
| GO:0050662 | coenzyme binding | F | 43 | 0.00302 | 0.01991 | 0.00219 | 0.98846 |
| GO:0017076 | purine nucleotide binding | F | 213 | 0.00263 | 0.02394 | 0.00205 | 0.98103 |
| GO:0000166 | nucleotide binding | F | 246 | 0.00262 | 0.02417 | 0.00215 | 0.98052 |
| GO:0006091 | generation of precursor metabolites and energy | P | 54 | 0.00352 | 0.02708 | 0.00252 | 0.98293 |
| GO:0045333 | cellular respiration | P | 29 | 0.00419 | 0.02868 | 0.00225 | 0.98478 |
| GO:0015980 | energy derivation by oxidation of organic compounds | P | 31 | 0.00383 | 0.02978 | 0.00216 | 0.98378 |
| GO:0001882 | nucleoside binding | F | 170 | 0.00277 | 0.03034 | 0.00212 | 0.97640 |
| GO:0001883 | purine nucleoside binding | F | 169 | 0.00273 | 0.03178 | 0.00212 | 0.97524 |
| GO:0030554 | adenyl nucleotide binding | F | 168 | 0.00273 | 0.03227 | 0.00212 | 0.97486 |
| GO:0032555 | purine ribonucleotide binding | F | 197 | 0.00265 | 0.03529 | 0.00206 | 0.97187 |
| GO:0032553 | ribonucleotide binding | F | 197 | 0.00265 | 0.03529 | 0.00206 | 0.97187 |
| GO:0019725 | cellular homeostasis | P | 25 | 0.00304 | 0.04355 | 0.00204 | 0.97697 |
| GO:0032559 | adenyl ribonucleotide binding | F | 152 | 0.00276 | 0.04689 | 0.00213 | 0.96332 |
| GO:0005524 | ATP binding | F | 151 | 0.00276 | 0.04735 | 0.00213 | 0.96297 |
| GO:0003779 | actin binding | F | 27 | 0.00287 | 0.05268 | 0.00244 | 0.97078 |
| GO:0019941 | modification-dependent protein catabolic process | P | 47 | 0.00250 | 0.05544 | 0.00206 | 0.96414 |
| GO:0043632 | modification-dependent macromolecule catabolic process | P | 47 | 0.00250 | 0.05544 | 0.00206 | 0.96414 |
| GO:0016879 | ligase activity, forming carbon-nitrogen bonds | F | 28 | 0.00321 | 0.06427 | 0.00231 | 0.96309 |
| GO:0033043 | regulation of organelle organization | P | 20 | 0.00236 | 0.06506 | 0.00104 | 0.96645 |
| GO:0030036 | actin cytoskeleton organization | P | 26 | 0.00149 | 0.06691 | 0.00187 | 0.96224 |
| GO:0030029 | actin filament-based process | P | 26 | 0.00149 | 0.06691 | 0.00187 | 0.96224 |
| GO:0044257 | cellular protein catabolic process | P | 52 | 0.00263 | 0.06908 | 0.00218 | 0.95343 |
| GO:0051603 | proteolysis involved in cellular protein catabolic process | P | 52 | 0.00263 | 0.06908 | 0.00218 | 0.95343 |
| GO:0044265 | cellular macromolecule catabolic process | P | 55 | 0.00262 | 0.08678 | 0.00233 | 0.93963 |
| GO:0030163 | protein catabolic process | P | 59 | 0.00236 | 0.09509 | 0.00198 | 0.93245 |
| GO:0016881 | acid-amino acid ligase activity | F | 25 | 0.00334 | 0.10044 | 0.00259 | 0.94089 |
| GO:0004842 | ubiquitin-protein ligase activity | F | 21 | 0.00350 | 0.10172 | 0.00226 | 0.94306 |
| GO:0019787 | small conjugating protein ligase activity | F | 21 | 0.00350 | 0.10251 | 0.00227 | 0.94255 |
| GO:0008092 | cytoskeletal protein binding | F | 34 | 0.00293 | 0.10833 | 0.00282 | 0.93033 |
| GO:0009057 | macromolecule catabolic process | P | 63 | 0.00238 | 0.11318 | 0.00212 | 0.91744 |
| GO:0007010 | cytoskeleton organization | P | 90 | 0.00270 | 0.13127 | 0.00297 | 0.89787 |
| GO:0051188 | cofactor biosynthetic process | P | 22 | 0.00315 | 0.14168 | 0.00363 | 0.91537 |
| GO:0008104 | protein localization | P | 71 | 0.00310 | 0.14204 | 0.00250 | 0.89234 |
| GO:0006457 | protein folding | P | 25 | 0.00227 | 0.17006 | 0.00234 | 0.89193 |
| GO:0034660 | ncRNA metabolic process | P | 30 | 0.00351 | 0.19162 | 0.00300 | 0.87079 |
| GO:0006399 | tRNA metabolic process | P | 23 | 0.00375 | 0.19187 | 0.00279 | 0.87817 |
| GO:0031967 | organelle envelope | C | 71 | 0.00323 | 0.22551 | 0.00273 | 0.82161 |
| GO:0031975 | envelope | C | 71 | 0.00323 | 0.22551 | 0.00273 | 0.82161 |
| GO:0006119 | oxidative phosphorylation | P | 27 | 0.00296 | 0.23710 | 0.00297 | 0.83835 |
| GO:0065003 | macromolecular complex assembly | P | 41 | 0.00185 | 0.24942 | 0.00182 | 0.81517 |
| GO:0008380 | RNA splicing | P | 28 | 0.00215 | 0.25090 | 0.00190 | 0.82608 |
| GO:0032561 | guanyl ribonucleotide binding | F | 49 | 0.00224 | 0.25630 | 0.00186 | 0.80421 |
| GO:0019001 | guanyl nucleotide binding | F | 49 | 0.00224 | 0.25630 | 0.00186 | 0.80421 |
| GO:0043933 | macromolecular complex subunit organization | P | 46 | 0.00184 | 0.25915 | 0.00194 | 0.80356 |
| GO:0006461 | protein complex assembly | P | 28 | 0.00174 | 0.26181 | 0.00179 | 0.81723 |
| GO:0070271 | protein complex biogenesis | P | 28 | 0.00174 | 0.26181 | 0.00179 | 0.81723 |
| GO:0007267 | cell-cell signaling | P | 39 | 0.00208 | 0.26623 | 0.00305 | 0.80250 |
| GO:0007268 | synaptic transmission | P | 37 | 0.00208 | 0.28116 | 0.00306 | 0.79154 |
| GO:0019226 | transmission of nerve impulse | P | 37 | 0.00208 | 0.28116 | 0.00306 | 0.79154 |
| GO:0022900 | electron transport chain | P | 20 | 0.00287 | 0.28677 | 0.00199 | 0.81023 |
| GO:0016310 | phosphorylation | P | 59 | 0.00239 | 0.28927 | 0.00237 | 0.77022 |
| GO:0005743 | mitochondrial inner membrane | C | 51 | 0.00330 | 0.30547 | 0.00327 | 0.76011 |
| GO:0005740 | mitochondrial envelope | C | 59 | 0.00324 | 0.30761 | 0.00322 | 0.75386 |
| GO:0009055 | electron carrier activity | F | 34 | 0.00253 | 0.30778 | 0.00251 | 0.77172 |
| GO:0019866 | organelle inner membrane | C | 52 | 0.00303 | 0.31382 | 0.00298 | 0.75208 |
| GO:0000398 | nuclear mRNA splicing, via spliceosome | P | 24 | 0.00182 | 0.31571 | 0.00166 | 0.77879 |
| GO:0000377 | RNA splicing, via transesterification reactions with bulged adenosine as nucleophile | P | 24 | 0.00182 | 0.31571 | 0.00166 | 0.77879 |
| GO:0000375 | RNA splicing, via transesterification reactions | P | 24 | 0.00182 | 0.31571 | 0.00166 | 0.77879 |
| GO:0016053 | organic acid biosynthetic process | P | 21 | 0.00369 | 0.31764 | 0.00161 | 0.78305 |
| GO:0046394 | carboxylic acid biosynthetic process | P | 21 | 0.00369 | 0.31764 | 0.00161 | 0.78305 |
| GO:0031966 | mitochondrial membrane | C | 54 | 0.00323 | 0.32779 | 0.00325 | 0.73838 |
| GO:0030170 | pyridoxal phosphate binding | F | 22 | 0.00186 | 0.33643 | 0.00163 | 0.76527 |
| GO:0070279 | vitamin B6 binding | F | 22 | 0.00186 | 0.33643 | 0.00163 | 0.76527 |
| GO:0031090 | organelle membrane | C | 89 | 0.00224 | 0.33723 | 0.00238 | 0.71563 |
| GO:0044455 | mitochondrial membrane part | C | 27 | 0.00320 | 0.34448 | 0.00269 | 0.74925 |
| GO:0010324 | membrane invagination | P | 46 | 0.00184 | 0.35174 | 0.00182 | 0.72207 |
| GO:0006897 | endocytosis | P | 46 | 0.00184 | 0.35174 | 0.00182 | 0.72207 |
| GO:0034622 | cellular macromolecular complex assembly | P | 28 | 0.00199 | 0.35831 | 0.00220 | 0.73563 |
| GO:0034621 | cellular macromolecular complex subunit organization | P | 35 | 0.00180 | 0.35923 | 0.00208 | 0.72553 |
| GO:0006793 | phosphorus metabolic process | P | 78 | 0.00224 | 0.36188 | 0.00204 | 0.69620 |
| GO:0006796 | phosphate metabolic process | P | 78 | 0.00224 | 0.36188 | 0.00204 | 0.69620 |
| GO:0003924 | GTPase activity | F | 39 | 0.00151 | 0.36350 | 0.00167 | 0.71748 |
| GO:0006511 | ubiquitin-dependent protein catabolic process | P | 21 | 0.00227 | 0.37423 | 0.00331 | 0.73526 |
| GO:0006396 | RNA processing | P | 54 | 0.00213 | 0.40368 | 0.00264 | 0.66864 |
| GO:0006909 | phagocytosis | P | 34 | 0.00160 | 0.40408 | 0.00161 | 0.68619 |
| GO:0006397 | mRNA processing | P | 37 | 0.00220 | 0.40410 | 0.00206 | 0.68261 |
| GO:0016071 | mRNA metabolic process | P | 38 | 0.00222 | 0.41075 | 0.00209 | 0.67538 |
| GO:0012505 | endomembrane system | C | 39 | 0.00185 | 0.41535 | 0.00164 | 0.67005 |
| GO:0008135 | translation factor activity, nucleic acid binding | F | 30 | 0.00230 | 0.42451 | 0.00255 | 0.67301 |
| GO:0016044 | membrane organization | P | 56 | 0.00179 | 0.42687 | 0.00185 | 0.64550 |
| GO:0001505 | regulation of neurotransmitter levels | P | 22 | 0.00307 | 0.42952 | 0.00347 | 0.68388 |
| GO:0042592 | homeostatic process | P | 33 | 0.00217 | 0.43600 | 0.00217 | 0.65800 |
| GO:0006412 | translation | P | 84 | 0.00321 | 0.43744 | 0.00294 | 0.62237 |
| GO:0016192 | vesicle-mediated transport | P | 73 | 0.00228 | 0.44127 | 0.00216 | 0.62292 |
| GO:0007269 | neurotransmitter secretion | P | 20 | 0.00516 | 0.44294 | 0.00314 | 0.67693 |
| GO:0003001 | generation of a signal involved in cell-cell signaling | P | 20 | 0.00516 | 0.44294 | 0.00314 | 0.67693 |
| GO:0006911 | phagocytosis, engulfment | P | 33 | 0.00159 | 0.45187 | 0.00158 | 0.64312 |
| GO:0004386 | helicase activity | F | 20 | 0.00200 | 0.45847 | 0.00331 | 0.66271 |
| GO:0008289 | lipid binding | F | 24 | 0.00388 | 0.46652 | 0.00238 | 0.64513 |
| GO:0008610 | lipid biosynthetic process | P | 25 | 0.00228 | 0.48953 | 0.00180 | 0.62119 |
| GO:0007052 | mitotic spindle organization | P | 48 | 0.00716 | 0.50468 | 0.00486 | 0.57622 |
| GO:0007051 | spindle organization | P | 49 | 0.00726 | 0.52618 | 0.00497 | 0.55416 |
| GO:0006413 | translational initiation | P | 22 | 0.00258 | 0.53874 | 0.00224 | 0.58070 |
| GO:0003743 | translation initiation factor activity | F | 22 | 0.00223 | 0.54289 | 0.00227 | 0.57660 |
| GO:0022402 | cell cycle process | P | 77 | 0.00289 | 0.57019 | 0.00252 | 0.49371 |
| GO:0022403 | cell cycle phase | P | 69 | 0.00339 | 0.57408 | 0.00281 | 0.49337 |
| GO:0046907 | intracellular transport | P | 63 | 0.00345 | 0.57821 | 0.00307 | 0.49232 |
| GO:0000279 | M phase | P | 67 | 0.00345 | 0.57976 | 0.00286 | 0.48859 |
| GO:0044432 | endoplasmic reticulum part | C | 22 | 0.00167 | 0.58100 | 0.00147 | 0.53843 |
| GO:0000278 | mitotic cell cycle | P | 70 | 0.00372 | 0.58214 | 0.00290 | 0.48467 |
| GO:0005525 | GTP binding | F | 48 | 0.00185 | 0.59281 | 0.00168 | 0.48770 |
| GO:0000226 | microtubule cytoskeleton organization | P | 59 | 0.00472 | 0.59399 | 0.00426 | 0.47854 |
| GO:0006886 | intracellular protein transport | P | 35 | 0.00308 | 0.59721 | 0.00313 | 0.49706 |
| GO:0015031 | protein transport | P | 54 | 0.00290 | 0.59759 | 0.00245 | 0.47815 |
| GO:0045184 | establishment of protein localization | P | 54 | 0.00290 | 0.59759 | 0.00245 | 0.47815 |
| GO:0034613 | cellular protein localization | P | 36 | 0.00331 | 0.61188 | 0.00338 | 0.48054 |
| GO:0070727 | cellular macromolecule localization | P | 42 | 0.00344 | 0.61924 | 0.00358 | 0.46594 |
| GO:0007049 | cell cycle | P | 85 | 0.00286 | 0.61978 | 0.00244 | 0.43975 |
| GO:0000502 | proteasome complex | C | 22 | 0.00122 | 0.63291 | 0.00131 | 0.48460 |
| GO:0046903 | secretion | P | 24 | 0.00400 | 0.64371 | 0.00284 | 0.46804 |
| GO:0016887 | ATPase activity | F | 62 | 0.00258 | 0.65489 | 0.00310 | 0.41330 |
| GO:0032940 | secretion by cell | P | 23 | 0.00459 | 0.65565 | 0.00358 | 0.45769 |
| GO:0005794 | Golgi apparatus | C | 32 | 0.00176 | 0.68792 | 0.00152 | 0.40507 |
| GO:0030529 | ribonucleoprotein complex | C | 74 | 0.00300 | 0.73315 | 0.00275 | 0.32347 |
| GO:0007017 | microtubule-based process | P | 71 | 0.00512 | 0.74190 | 0.00448 | 0.31510 |
| GO:0042623 | ATPase activity, coupled | F | 53 | 0.00276 | 0.78366 | 0.00348 | 0.27745 |
| GO:0006163 | purine nucleotide metabolic process | P | 21 | 0.00221 | 0.84044 | 0.00289 | 0.24577 |
| GO:0015399 | primary active transmembrane transporter activity | F | 26 | 0.00320 | 0.87421 | 0.00325 | 0.19237 |
| GO:0015405 | P-P-bond-hydrolysis-driven transmembrane transporter activity | F | 26 | 0.00320 | 0.87421 | 0.00325 | 0.19237 |
| GO:0015934 | large ribosomal subunit | C | 22 | 0.00266 | 0.88941 | 0.00352 | 0.17831 |
| GO:0044445 | cytosolic part | C | 27 | 0.00684 | 0.90969 | 0.00586 | 0.14310 |
| GO:0043492 | ATPase activity, coupled to movement of substances | F | 24 | 0.00273 | 0.91769 | 0.00300 | 0.13549 |
| GO:0042626 | ATPase activity, coupled to transmembrane movement of substances | F | 24 | 0.00273 | 0.91769 | 0.00300 | 0.13549 |
| GO:0016820 | hydrolase activity, acting on acid anhydrides, catalyzing transmembrane movement of substances | F | 24 | 0.00273 | 0.91769 | 0.00300 | 0.13549 |
| GO:0022890 | inorganic cation transmembrane transporter activity | F | 24 | 0.00231 | 0.92085 | 0.00314 | 0.13096 |
| GO:0033279 | ribosomal subunit | C | 37 | 0.00351 | 0.93106 | 0.00374 | 0.10546 |
| GO:0005840 | ribosome | C | 37 | 0.00351 | 0.93106 | 0.00374 | 0.10546 |
| GO:0003735 | structural constituent of ribosome | F | 37 | 0.00351 | 0.93106 | 0.00374 | 0.10546 |
| GO:0034654 | nucleobase, nucleoside, nucleotide and nucleic acid biosynthetic process | P | 28 | 0.00216 | 0.94917 | 0.00295 | 0.08547 |
| GO:0034404 | nucleobase, nucleoside and nucleotide biosynthetic process | P | 28 | 0.00216 | 0.94917 | 0.00295 | 0.08547 |
| GO:0044271 | nitrogen compound biosynthetic process | P | 48 | 0.00288 | 0.95613 | 0.00313 | 0.06641 |
| GO:0022626 | cytosolic ribosome | C | 21 | 0.00470 | 0.98422 | 0.00481 | 0.03291 |
| GO:0009165 | nucleotide biosynthetic process | P | 24 | 0.00172 | 0.98615 | 0.00299 | 0.02798 |
| GO:0009260 | ribonucleotide biosynthetic process | P | 20 | 0.00179 | 0.98683 | 0.00319 | 0.02854 |
| GO:0009259 | ribonucleotide metabolic process | P | 21 | 0.00182 | 0.98766 | 0.00322 | 0.02646 |
| GO:0000022 | mitotic spindle elongation | P | 23 | 0.00459 | 0.99228 | 0.00617 | 0.01681 |
| GO:0051231 | spindle elongation | P | 23 | 0.00459 | 0.99228 | 0.00617 | 0.01681 |

F: molecular function; P: biological process; C: cellular component
