# Supplementary material for: The genome of the Antarctic-endemic copepod, Tigriopus kingsejongensis
Source: Gigascience. 2017 Jan 7;6(1):1–9. doi: 10.1093/gigascience/giw010 (PMC5467011; doi:10.1093/gigascience/giw010)
Supplement: Table S11. — Lists and annotations of positively selected genes in the Tigriopus kingsejongensis genome. [file giw010_TableS11.docx]

Table S11.

| **Gene ID** | **Flybase ID** | **Gene symbol** | **Gene description** | ***P*-value** | ***q*-value (FDR 10%)** |
| --- | --- | --- | --- | --- | --- |
| Tk08564 | FBgn0010352 | Nc73EF | Neural conserved at 73EF | 3.7E-11 | 0 |
| Tk09239 | FBgn0000212 | brm | brahma | 1.3E-10 | 0 |
| Tk07819 | FBgn0039959 | CG17514 | Dmel_CG17514 | 2.2E-09 | 0 |
| Tk01832 | FBgn0038826 | Syp | Dmel_CG17838 | 7.0E-08 | 5.7E-06 |
| Tk01804 | FBgn0032690 | CG10333 | Dmel_CG10333 | 2.6E-07 | 1.3E-05 |
| Tk05312 | FBgn0024191 | sip1 | no description | 1.1E-06 | 4.1E-05 |
| Tk01960 | FBgn0000559 | EF2 | Elongation factor 2b | 1.8E-06 | 5.7E-05 |
| Tk06425 | FBgn0037051 | CG10565 | Dmel_CG10565 | 4.4E-06 | 1.1E-04 |
| Tk12018 | FBgn0039874 | betaGlu | Beta-glucuronidase | 5.7E-06 | 1.3E-04 |
| Tk01342 | FBgn0026702 | l(1)G0045 | lethal (1) G0045 | 8.3E-06 | 1.7E-04 |
| Tk01315 | FBgn0021768 | nudC | Dmel_CG9710 | 2.6E-05 | 3.8E-04 |
| Tk10596 | FBgn0039688 | Kul | Kuzbanian-like | 4.6E-05 | 5.2E-04 |
| Tk02580 | FBgn0030718 | CG9172 | Dmel_CG9172 | 7.9E-05 | 7.6E-04 |
| Tk03943 | FBgn0267385 | PyK | no description | 8.1E-05 | 7.7E-04 |
| Tk01236 | FBgn0032635 | CG15141 | Dmel_CG15141 | 2.3E-04 | 1.7E-03 |
| Tk04720 | FBgn0033998 | row | Dmel_CG8092 | 2.8E-04 | 1.9E-03 |
| Tk09657 | FBgn0028699 | Rh50 | Dmel_CG7499 | 3.9E-04 | 2.5E-03 |
| Tk09525 | FBgn0027052 | CHIP | Dmel_CG5203 | 4.9E-04 | 3.0E-03 |
| Tk11036 | FBgn0039252 | CG11771 | Dmel_CG11771 | 5.5E-04 | 3.3E-03 |
| Tk12598 | FBgn0053196 | dp | no description | 7.6E-04 | 3.9E-03 |
| Tk01697 | FBgn0036207 | CG10907 | Dmel_CG10907 | 9.0E-04 | 4.4E-03 |
| Tk06384 | FBgn0000150 | ndk | nucleoside diphosphate kinase b | 1.2E-03 | 7.7E-03 |
| Tk08174 | FBgn0086372 | lap | like-AP180 | 1.5E-03 | 6.5E-03 |
| Tk11865 | FBgn0034237 | eIF3-S9 | Eukaryotic translation initiation factor 3 subunit B | 1.7E-03 | 6.9E-03 |
| Tk06395 | FBgn0033428 | Updo | Uroporphyrinogen decarboxylase | 2.5E-03 | 8.6E-03 |
| Tk05687 | FBgn0010422 | TfIIS | RNA polymerase II elongation factor | 3.0E-03 | 9.6E-03 |
| Tk00731 | FBgn0032987 | RpL21 | Ribosomal protein L21 | 3.0E-03 | 9.7E-03 |
| Tk10975 | FBgn0086691 | UK114 | Dmel_CG15261 | 3.1E-03 | 9.7E-03 |
| Tk07082 | FBgn0263352 | Unr | no description | 3.5E-03 | 1.1E-02 |
| Tk08051 | FBgn0033339 | Sec31 | Dmel_CG8266 | 3.9E-03 | 1.1E-02 |
| Tk05720 | FBgn0036169 | Fuca | alpha-L-fucosidase | 4.2E-03 | 1.2E-02 |
| Tk02672 | FBgn0024889 | Kap-alpha1 | karyopherin alpha1 | 5.3E-03 | 1.4E-02 |
| Tk12713 | FBgn0011703 | RnrL | Ribonucleoside diphosphate reductase large subunit | 5.8E-03 | 1.4E-02 |
| Tk05916 | FBgn0032955 | chk | choline kinase alpha isoform | 7.1E-03 | 1.6E-02 |
| Tk08357 | FBgn0034109 | CG7747 | Dmel_CG7747 | 7.9E-03 | 1.7E-02 |
| Tk05319 | FBgn0005536 | Mbs | Myosin binding subunit | 8.4E-03 | 1.8E-02 |
| Tk06713 | FBgn0030151 | CG1354 | GTP-binding protein CG1354 | 8.6E-03 | 1.8E-02 |
| Tk12047 | FBgn0038234 | mRpL11 | mitochondrial ribosomal protein L11 | 9.3E-03 | 1.9E-02 |
| Tk05027 | FBgn0011202 | dia | diaphanous | 9.4E-03 | 1.9E-02 |
| Tk04694 | FBgn0025678 | CaBP1 | Dmel_CG5809 | 1.0E-02 | 2.0E-02 |
| Tk00257 | FBgn0032634 | Rpb11 | DNA-directed RNA polymerase II subunit RPB11 | 1.0E-02 | 2.0E-02 |
| Tk01522 | FBgn0052500 | CG32500 | Dmel_CG32857; Dmel_CG32500; Dmel_CG33502 | 1.1E-02 | 2.1E-02 |
| Tk04854 | FBgn0030460 | CG2453 | Ubiquinone biosynthesis methyltransferase COQ5, mitochondrial | 1.1E-02 | 2.1E-02 |
| Tk08219 | FBgn0035111 | CG16940 | Dmel_CG16940 | 1.1E-02 | 2.1E-02 |
| Tk08858 | FBgn0033902 | Tango7 | Transport and Golgi organization 7 | 1.1E-02 | 2.2E-02 |
| Tk12379 | FBgn0020626 | Osbp | Oxysterol binding protein | 1.2E-02 | 2.3E-02 |
| Tk05810 | FBgn0053203 | CG33203 | Dmel_CG33203 | 1.3E-02 | 2.3E-02 |
| Tk04582 | FBgn0026616 | alpha-Man-IIb | Dmel_CG4606 | 1.3E-02 | 2.3E-02 |
| Tk04050 | FBgn0029737 | CG6903 | Dmel_CG6903 | 1.3E-02 | 2.4E-02 |
| Tk11237 | FBgn0033196 | CG1358 | Dmel_CG1358 | 1.4E-02 | 2.5E-02 |
| Tk04109 | FBgn0034372 | Gint3 | GDI interacting protein 3 | 1.5E-02 | 2.6E-02 |
| Tk05877 | FBgn0025864 | Crag | Calmodulin-binding protein related to a Rab3 GDP/GTP exchange protein | 1.7E-02 | 2.8E-02 |
| Tk11784 | FBgn0010382 | CycE | Cyclin E | 1.7E-02 | 2.8E-02 |
| Tk06389 | FBgn0039016 | Dcr-1 | Dicer-1 | 1.7E-02 | 2.9E-02 |
| Tk01701 | FBgn0032050 | CG13096 | Ribosomal L1 domain-containing protein CG13096 | 1.8E-02 | 3.0E-02 |
| Tk12222 | FBgn0038401 | CG5916 | Dmel_CG5916 | 1.8E-02 | 3.0E-02 |
| Tk08074 | FBgn0024362 | CG11412 | N-acetyltransferase MAK3 homolog | 1.9E-02 | 3.0E-02 |
| Tk08316 | FBgn0037534 | CG2781 | Dmel_CG2781 | 2.0E-02 | 3.2E-02 |
| Tk03338 | FBgn0035533 | Cip4 | Dmel_CG15015 | 2.0E-02 | 3.2E-02 |
| Tk07649 | FBgn0031606 | CG15439 | Dmel_CG15439 | 2.2E-02 | 3.3E-02 |
| Tk10901 | FBgn0031779 | CG9175 | Dmel_CG9175 | 2.5E-02 | 3.6E-02 |
| Tk02297 | FBgn0010516 | ETFA | electron transfer flavoprotein subunit alpha | 2.5E-02 | 3.6E-02 |
| Tk00308 | FBgn0015477 | Rme-8 | Receptor mediated endocytosis 8 | 2.8E-02 | 3.9E-02 |
| Tk05833 | FBgn0025742 | mtm | myotubularin | 2.9E-02 | 4.0E-02 |
| Tk12338 | FBgn0027509 | CG7261 | Dmel_CG7261 | 3.0E-02 | 4.1E-02 |
| Tk05043 | FBgn0032429 | CG5446 | Dmel_CG5446 | 3.1E-02 | 4.1E-02 |
| Tk08058 | FBgn0021765 | scu | scully | 3.4E-02 | 4.3E-02 |
| Tk04105 | FBgn0034590 | Magi | Dmel_CG30388 | 3.4E-02 | 4.4E-02 |
| Tk11121 | FBgn0003308 | ry | rosy | 3.5E-02 | 4.4E-02 |
| Tk05297 | FBgn0011211 | ATP5A | mitochondrial atp synthase subunit alpha precursor | 3.5E-02 | 4.4E-02 |
| Tk00382 | FBgn0030578 | CG5347 | Dmel_CG5347 | 4.2E-02 | 5.1E-02 |
| Tk12689 | FBgn0261675 | Npc1b | no description | 4.5E-02 | 5.3E-02 |
| Tk07627 | FBgn0022764 | Sin3A | Dmel_CG8815 | 4.8E-02 | 5.5E-02 |
| Tk06311 | FBgn0038271 | UQCRC1 | mitochondrial-processing peptidase subunit beta | 4.9E-02 | 5.6E-02 |
