# Supplementary material for: The genome of the Antarctic-endemic copepod, Tigriopus kingsejongensis
Source: Gigascience. 2017 Jan 7;6(1):1–9. doi: 10.1093/gigascience/giw010 (PMC5467011; doi:10.1093/gigascience/giw010)
Supplement: Table S12. — Lists and annotations of positively selected genes in the Tigriopus japonicus genome. [file giw010_TableS12.docx]

Table S12.

| **Gene ID** | **Flybase ID** | **Gene symbol** | **Gene description** | ***P*-value** | ***q*-value (FDR 10%)** |
| --- | --- | --- | --- | --- | --- |
| Tj08472 | FBgn0001248 | Idh | Isocitrate dehydrogenase | 8.7E-10 | 4.3E-07 |
| Tj00582 | FBgn0000559 | EF2 | Elongation factor 2b | 6.9E-08 | 8.4E-06 |
| Tj01681 | FBgn0004656 | fs(1)h | female sterile (1) homeotic | 1.0E-07 | 1.1E-05 |
| Tj23687 | FBgn0010352 | Nc73EF | Neural conserved at 73EF | 1.8E-07 | 1.8E-05 |
| Tj00891 | FBgn0039959 | CG17514 | Dmel_CG17514 | 4.8E-07 | 3.9E-05 |
| Tj06989 | FBgn0003360 | sesB | stress-sensitive B | 9.6E-07 | 5.7E-05 |
| Tj05258 | FBgn0265297 | pAbp | no description | 1.3E-06 | 7.0E-05 |
| Tj37264 | FBgn0010247 | Parp | Poly-(ADP-ribose) polymerase | 1.6E-06 | 7.7E-05 |
| Tj02691 | FBgn0025574 | Pli | Pellino | 2.1E-06 | 9.4E-05 |
| Tj10850 | FBgn0037051 | CG10565 | Dmel_CG10565 | 3.9E-06 | 1.5E-04 |
| Tj00713 | FBgn0261985 | Ptpmeg | no description | 9.7E-06 | 2.9E-04 |
| Tj00243 | FBgn0039688 | Kul | Kuzbanian-like | 1.8E-05 | 4.8E-04 |
| Tj05123 | FBgn0030268 | Klp10A | Kinesin-like protein Klp10A | 2.2E-05 | 5.7E-04 |
| Tj10519 | FBgn0010408 | RpS9 | Ribosomal protein S9 | 2.6E-05 | 6.3E-04 |
| Tj01855 | FBgn0263006 | Ca-P60A | no description | 4.0E-05 | 8.1E-04 |
| Tj14139 | FBgn0038617 | CG12333 | Dmel_CG12333 | 1.0E-04 | 1.6E-03 |
| Tj08302 | FBgn0020626 | Osbp | Oxysterol binding protein | 1.4E-04 | 2.0E-03 |
| Tj02908 | FBgn0020621 | Pkn | Protein kinase related to protein kinase N | 1.5E-04 | 2.1E-03 |
| Tj00756 | FBgn0039627 | CG11837 | Probable dimethyladenosine transferase | 2.9E-04 | 3.4E-03 |
| Tj03364 | FBgn0033342 | CG8258 | Dmel_CG8258 | 3.0E-04 | 3.4E-03 |
| Tj18070 | FBgn0029737 | CG6903 | Dmel_CG6903 | 3.8E-04 | 3.8E-03 |
| Tj07251 | FBgn0033316 | CG14749 | Nucleoporin GLE1 | 5.6E-04 | 4.9E-03 |
| Tj13984 | FBgn0001139 | gro | groucho | 6.0E-04 | 5.2E-03 |
| Tj24438 | FBgn0039139 | Ime4 | Probable N6-adenosine-methyltransferase MT-A70-like protein | 6.7E-04 | 5.5E-03 |
| Tj06681 | FBgn0028699 | Rh50 | Dmel_CG7499 | 7.9E-04 | 6.0E-03 |
| Tj01236 | FBgn0025742 | mtm | myotubularin | 8.5E-04 | 6.2E-03 |
| Tj04229 | FBgn0036648 | CG4098 | Dmel_CG4098 | 8.6E-04 | 6.2E-03 |
| Tj12989 | FBgn0040493 | grsm | granny smith | 9.0E-04 | 6.4E-03 |
| Tj00402 | FBgn0035464 | CG12006 | GPI mannosyltransferase 3 | 9.2E-04 | 6.4E-03 |
| Tj07935 | FBgn0030482 | CG1673 | Branched-chain-amino-acid aminotransferase | 9.9E-04 | 6.6E-03 |
| Tj06965 | FBgn0038271 | CG3731 | Dmel_CG3731 | 1.0E-03 | 6.9E-03 |
| Tj00640 | FBgn0266465 | GckIII | no description | 1.1E-03 | 7.0E-03 |
| Tj06342 | FBgn0038269 | Rrp6 | Dmel_CG7292 | 1.2E-03 | 7.7E-03 |
| Tj08056 | FBgn0053094 | Synd | Syndapin | 1.3E-03 | 8.0E-03 |
| Tj21595 | FBgn0027549 | CG7927 | Dmel_CG7927 | 1.3E-03 | 8.0E-03 |
| Tj02349 | FBgn0263352 | Unr | no description | 1.5E-03 | 8.9E-03 |
| Tj05421 | FBgn0028687 | Rpt1 | Dmel_CG1341 | 1.7E-03 | 9.4E-03 |
| Tj40085 | FBgn0022153 | l(2)k05819 | lethal (2) k05819 | 1.7E-03 | 9.4E-03 |
| Tj03671 | FBgn0034237 | eIF3-S9 | Eukaryotic translation initiation factor 3 subunit B | 2.0E-03 | 1.0E-02 |
| Tj01044 | FBgn0031057 | Ubqn | Dmel_CG14224 | 2.2E-03 | 1.1E-02 |
| Tj09164 | FBgn0032997 | CG17486 | Asparagine synthetase domain-containing protein CG17486 | 2.5E-03 | 1.2E-02 |
| Tj29160 | FBgn0020270 | mre11 | meiotic recombination 11 | 2.7E-03 | 1.3E-02 |
| Tj08461 | FBgn0028325 | l(1)G0334 | lethal (1) G0334 | 2.8E-03 | 1.3E-02 |
| Tj03460 | FBgn0036184 | PCID2 | PCI domain-containing protein 2 homolog | 3.1E-03 | 1.4E-02 |
| Tj01525 | FBgn0036546 | elgi | Dmel_CG17033 | 3.2E-03 | 1.4E-02 |
| Tj07227 | FBgn0016123 | Aph-4 | Alkaline phosphatase 4 | 3.2E-03 | 1.4E-02 |
| Tj14885 | FBgn0017545 | RpS3A | Ribosomal protein S3A | 3.8E-03 | 1.6E-02 |
| Tj18326 | FBgn0032247 | CG5188 | Methionine aminopeptidase | 4.0E-03 | 1.6E-02 |
| Tj12964 | FBgn0034405 | Jheh2 | Juvenile hormone epoxide hydrolase 2 | 4.5E-03 | 1.8E-02 |
| Tj07993 | FBgn0035947 | Srp68 | Signal recognition particle 68 kDa protein | 4.5E-03 | 1.8E-02 |
| Tj06613 | FBgn0037580 | DppIII | Dipeptidyl aminopeptidase III | 4.9E-03 | 1.9E-02 |
| Tj17433 | FBgn0037270 | CG9769 | Eukaryotic translation initiation factor 3 subunit F-1 | 6.1E-03 | 2.3E-02 |
| Tj08113 | FBgn0265778 | PDZ-GEF | no description | 6.3E-03 | 2.3E-02 |
| Tj00752 | FBgn0020443 | Elf | Ef1alpha-like factor | 6.3E-03 | 2.3E-02 |
| Tj03319 | FBgn0003165 | pum | pumilio | 7.4E-03 | 2.7E-02 |
| Tj11864 | FBgn0027509 | CG7261 | Dmel_CG7261 | 7.7E-03 | 2.7E-02 |
| Tj02364 | FBgn0000455 | Dip-C | small non-messenger RNA 649; Dipeptidase C | 8.5E-03 | 2.9E-02 |
| Tj13184 | FBgn0038869 | CG3353 | Dmel_CG3353 | 8.6E-03 | 2.9E-02 |
| Tj11772 | FBgn0267790 | rump | no description | 8.6E-03 | 2.9E-02 |
| Tj14430 | FBgn0038956 | CG5379 | Dmel_CG5379 | 8.6E-03 | 2.9E-02 |
| Tj00894 | FBgn0261618 | larp | no description | 9.8E-03 | 3.2E-02 |
| Tj00068 | FBgn0033339 | Sec31 | Dmel_CG8266 | 1.0E-02 | 3.3E-02 |
| Tj33740 | FBgn0039111 | Plip | PTEN-like phosphatase | 1.0E-02 | 3.3E-02 |
| Tj04776 | FBgn0039773 | CG2224 | Dmel_CG2224 | 1.1E-02 | 3.5E-02 |
| Tj08373 | FBgn0002022 | Catsup | Catecholamines up | 1.4E-02 | 4.0E-02 |
| Tj00308 | FBgn0014002 | Pdi | Protein disulfide isomerase | 1.5E-02 | 4.2E-02 |
| Tj31490 | FBgn0020269 | mspo | M-spondin | 1.7E-02 | 4.5E-02 |
| Tj09265 | FBgn0030407 | CG2543 | Dmel_CG2543 | 1.9E-02 | 4.8E-02 |
| Tj00079 | FBgn0036125 | CG6279 | Dmel_CG6279 | 1.9E-02 | 4.9E-02 |
| Tj00377 | FBgn0259984 | kuz | kuzbanian | 2.2E-02 | 5.4E-02 |
| Tj24226 | FBgn0033060 | CG7849 | Dmel_CG7849 | 2.4E-02 | 5.8E-02 |
| Tj03066 | FBgn0031450 | Hrs | Hepatocyte growth factor regulated tyrosine kinase substrate | 2.5E-02 | 5.9E-02 |
| Tj06835 | FBgn0005777 | PpD3 | Protein phosphatase D3 | 3.0E-02 | 6.7E-02 |
| Tj07564 | FBgn0011300 | babo | baboon | 3.1E-02 | 6.8E-02 |
| Tj24271 | FBgn0028697 | RpL15 | Ribosomal protein L15 | 3.4E-02 | 7.3E-02 |
| Tj05632 | FBgn0031779 | CG9175 | Dmel_CG9175 | 3.4E-02 | 7.3E-02 |
| Tj02249 | FBgn0025936 | Eph | Eph receptor tyrosine kinase | 3.5E-02 | 7.4E-02 |
| Tj03965 | FBgn0032781 | CG9987 | Dmel_CG9987 | 3.8E-02 | 7.9E-02 |
| Tj13328 | FBgn0262740 | Evi5 | no description | 4.0E-02 | 8.0E-02 |
