# Supplementary material for: The genome of the Antarctic-endemic copepod, Tigriopus kingsejongensis
Source: Gigascience. 2017 Jan 7;6(1):1–9. doi: 10.1093/gigascience/giw010 (PMC5467011; doi:10.1093/gigascience/giw010)
Supplement: Table S13. — Enriched Gene Ontology (GO) categories identified by positively selected genes from the Tigriopus kingsejongensis genome. REVIGO software was used to cluster related GO terms (in bold letters) according to P-value. [file giw010_TableS13.docx]

Table S13.

| GO IDs | GO descriptions | Category | No. of genes | No. of genes (%) | *P*-value |
| --- | --- | --- | --- | --- | --- |
| **GO:0006119** | **oxidative phosphorylation** | P | 3 | 7.04 | 8.8E-03 |
| GO:0015985 | energy coupled proton transport, down electrochemical  gradient | P | 3 | 4.23 | 1.1E-02 |
| GO:0015986 | ATP synthesis coupled proton transport | P | 6 | 8.45 | 1.6E-02 |
| **GO:0022890** | **inorganic cation transmembrane transporter activity** | F | 5 | 7.04 | 4.5E-02 |
| GO:0015078 | hydrogen ion transmembrane transporter activity | F | 4 | 5.63 | 1.3E-02 |
| GO:0015077 | monovalent inorganic cation transmembrane transporter  activity | F | 4 | 5.63 | 1.8E-02 |
| **GO:0045259** | **proton-transporting ATP synthase complex** | C | 3 | 4.23 | 3.5E-02 |
| GO:0005753 | mitochondrial proton-transporting ATP synthase complex | C | 3 | 4.23 | 3.7E-02 |
| **GO:0032268** | **regulation of cellular protein metabolic process** | P | 5 | 7.04 | 1.7E-02 |
| **GO:0007277** | **pole cell development** | P | 3 | 4.23 | 8.8E-03 |
| **GO:0006091** | **generation of precursor metabolites and energy** | P | 3 | 4.23 | 9.3E-03 |
| **GO:0010608** | **posttranscriptional regulation of gene expression** | P | 4 | 5.63 | 6.7E-03 |
| **GO:0005811** | **lipid particle** | C | 7 | 9.86 | 6.7E-03 |
| **GO:0044455** | **mitochondrial membrane part** | C | 5 | 7.04 | 2.5E-02 |
| **GO:0004386** | **helicase activity** | F | 4 | 5.63 | 4.9E-02 |
| **GO:0051536** | **iron-sulfur cluster binding** | F | 4 | 5.63 | 4.8E-02 |
| **GO:0008135** | **translation factor activity, nucleic acid binding** | F | 4 | 5.63 | 4.8E-02 |
| **GO:0051540** | **metal cluster binding** | F | 4 | 5.63 | 1.5E-02 |

F: molecular function; P: biological process; C: cellular component
