# Supplementary material for: The genome of the Antarctic-endemic copepod, Tigriopus kingsejongensis
Source: Gigascience. 2017 Jan 7;6(1):1–9. doi: 10.1093/gigascience/giw010 (PMC5467011; doi:10.1093/gigascience/giw010)
Supplement: Table S14. — Enriched Gene Ontology (GO) categories identified by positively selected genes from the Tigriopus japonicus genome. REVIGO software was used to cluster related GO terms (in bold letters) according to P-value. [file giw010_TableS14.docx]

Table S14.

| **GO IDs** | **GO descriptions** | **Category** | **No. of genes** | **No. of genes (%)** | ***P*-value** |
| --- | --- | --- | --- | --- | --- |
| **GO:0004177** | **aminopeptidase activity** | F | 4 | 5.71 | 3.5E-03 |
| GO:0008238 | exopeptidase activity | F | 4 | 5.71 | 3.5E-02 |
| GO:0008237 | metallopeptidase activity | F | 6 | 8.57 | 5.5E-03 |
| **GO:0005829** | **cytosol** | C | 10 | 14.29 | 4.1E-03 |
| **GO:0000278** | **mitotic cell cycle** | P | 8 | 11.43 | 1.0E-02 |
| **GO:0008237** | **metallopeptidase activity** | F | 6 | 8.57 | 5.5E-03 |
| **GO:0007052** | **mitotic spindle organization** | P | 5 | 7.14 | 4.2E-02 |
| **GO:0008135** | **translation factor activity, nucleic acid binding** | F | 4 | 5.71 | 1.9E-02 |

F: molecular function; P: biological process; C: cellular component
