# Supplementary material for: The genome of the Antarctic-endemic copepod, Tigriopus kingsejongensis
Source: Gigascience. 2017 Jan 7;6(1):1–9. doi: 10.1093/gigascience/giw010 (PMC5467011; doi:10.1093/gigascience/giw010)
Supplement: Response_to_Reviewer_Comments_Original_Submission.pdf [file giw010_Response_to_Reviewer_Comments_Original_Submission.pdf]

Subject: Your submission to GigaScience - GIGA-D-16-00040

GIGA-D-16-00040
The First Copepod Genome Reveals Evolutionary Adaptation to Extreme Environments in the Antartic-endemic Tigriopus Seunghyun Kang; Do-Hwan Ahn; Jun Hyuck Lee; Sung Gu Lee; Seung Chul Shin; Jungeun Lee; Gi-Sik Min; Hyoungseok Lee; Hyun-Woo Kim; Sanghee Kim; Hyun Park GigaScience

Dear Hans,
We would like to thank you and all the reviewers for your kind help to revise our manuscript and consider our manuscript for publication in GigaScience. We have decided to change the article type from "Research" to "Data Note" following your precious suggestion. Accordingly, we have reduced biological analysis part (especially transcriptome response to temperature stress), focused on genome part in response to Data Note criteria and wrote down response to reviewer comments about remaining parts. As we have changed the article type, we also modified the title as follows: "The genome of Antarctic-endemic Copepod Tigriopus kingsejongensis".
We appreciated all of the comments and suggestions and carefully considered all of them during the revision. All of inferred statements have been corrected, and also all of mistakes have been corrected in the revised manuscript. The corrected points were marked in yellow color in revised manuscript.
We did our best to address the comments from the reviewers. Hope the revised is acceptable for publication.
We look forward to hearing your decision.
Thanks and best regards.
Hyun Park

Reviewer reports:
Reviewer #1: The paper describes the genome of a recently described species of the harpacticoid genus Tigriopus. The species is interesting as it lives in the cold Antarctic environment. If published, it may well be the first paper describing a copepod genome; however, it should be noted that other copepod genomes are already available online, including the congener Tigriopus californicus (https://i5k.nal.usda.gov/Tigriopus_californicus). Some reference to this and other copepod genomes (Eurytemora affinis and Salmon louse) might be appropriate.

Response) We added publically available copepod genome data in data description part line 30.

The abstract and background of this paper both open with what is clearly a false statement - there are not more species of copepods than insects or nematodes; there are over 1 million species of insects vs. ~12,000 species of copepods, so it's not clear what the authors are stating. Perhaps this is a language problem, but it results in a very significant error.

Response) We made clear the sentences in abstract and data description line 21.

Background line 24 - outdated refs for temperature adaptation. Full transcriptome response to heat stress in T. californicus was published 4 years ago (Schoville et al 2012 BMC Evolutionary Biology) and would seem to be an especially appropriate reference here as the authors could contrast response to cold with the response to heat. (also consider Barreto et al. 2011 Interpopulation patterns of divergence and selection across the transcriptome of the copepod Tigriopus californicus. Molecular Ecology. 20:560-572). It might be interesting to see if there is any overlap between genes identified as under positive selection in this species versus those identified as under selection between T. californicus populations (see Pereira et al. 2016 Molecular Ecology).

Response) We thank the reviewer's valuable comments about transcriptome response to temperature stress. At this moment, we have changed article type from "research note" to "data note" and need to reduce biological analysis. Following your suggestions, we are going to compare not only T. japonicus but also T. californicus in our future study.

As for the genome data itself, the assembly reported is interesting as the genome size is substantially larger than that of T. californicus (size based on nuclear fluorescence is ~240Mb). The assembly is rather fragmented >11,000 scaffolds and I wonder if they might see significant improvement if they used a different assembler (maybe try ALLOPATHS instead of Celera - they have the necessary data)?

Response) We have used the three assemblers, Abyss, SOAP, and Celera, and all the assembly resulted in almost same genome size about 295 Mb in accordance with k-mer genome size estimation. The best statistical results were obtained from Celera and we have used Celera assembly results in the following analysis processes. We have constructed relatively long paired-end library datasets: 350bp, 400bp, 450bp, and 500bp. As far as we know, AllpathLG need at least 20 % overlapping "fragment library". Unfortunately, AllpathLG cannot be applied to our datasets.

The completeness statistic based on coverage of CEGMA is not great (82%) but they used the larger set of 458 conserved proteins rather than the more conservative set of 248 proteins often used. The authors do not report what percentage of the genes are fully (vs. partially) covered in their assembly - this might make it easier for readers to better interpret the results. I think this may be an issue if their assembly is rather incomplete in total coverage, the reported gains and losses of gene families may be unreliable. The number of annotated gene models 12,772 is a bit low (10% lower than the smaller T. californicus genome at ~14,100).
Response) We have modified and added complete and partial annotated gene numbers and percentages in data description line 129.

Reviewer #2:

However, I quickly became a bit perturbed. The authors claim that genomes and genomic resources are lacking for copepods, and that this is the first copepod genome paper. This is an odd statement, given that there are more genomes freely available for copepods than for any other crustacean, and a plethora of genomic resources available, relative to other non-insect arthropods. The salmon louse genome project had an official press release five years ago, and is available for analysis. In addition, two other copepod genomes, those of Tigriopus californicus and Eurytemora affinis, are freely available from the Arthropod i5K website, and have been available for over two years. And this study does make a comparison with the genome of Tigriopus japonicus, which has been around for a while. I have seen some comparative studies that incorporate analyses of these other copepod genomes, without calling them the "first genome." There are also many copepod transcriptomes freely available.

Response) The meaning of our previous title was the first genome paper describing copepods. Following your suggestions, we modified the title and added publically available copepod genome data in data description part line 30.

Given the availability of the other copepod genomes, this study would benefit from comparisons with at least the congener Tigriopus californicus, in addition to the congener T. japonicus.

Response) Thank you so much for your informative comments. At this moment, we have changed article type from "research note" to "data note" and need to reduce biological analysis. Following your valuable suggestions, we are going to compare T. californicus genome and transcriptome with T. kingsejongensis and T. japonicus in our future study.

The authors applied PAML to test for signatures of selection. I recommend that they also use HyPhy, which is more powerful, and able to make greater inferences than PAML.

Response) We have used PAML in this study according to following articles and we will apply HyPhy in the future study.
Cao, Z., et al. (2013). "The genome of Mesobuthus martensii reveals a unique adaptation model of

arthropods." Nature communications 4: 2602.

Neafsey, D. E., et al. (2015). "Highly evolvable malaria vectors: The genomes of 16 Anopheles mosquitoes." Science 347(6217): 1258522.

Qiu, Q., et al. (2012). "The yak genome and adaptation to life at high altitude." Nature Genetics 44(8): 946-949.

Yim, H.-S., et al. (2014). "Minke whale genome and aquatic adaptation in cetaceans." Nature Genetics 46(1): 88-92.
