# Supplementary material for: The genome of the Antarctic-endemic copepod, Tigriopus kingsejongensis
Source: Gigascience. 2017 Jan 7;6(1):1–9. doi: 10.1093/gigascience/giw010 (PMC5467011; doi:10.1093/gigascience/giw010)
Supplement: Response_to_Reviewer_Comments_Revision_2.pdf [file giw010_Response_to_Reviewer_Comments_Revision_2.pdf]

GIGA-D-16-00040
The genome of Antarctic-endemic Copepod, Tigriopus kingsejongensis
Seunghyun Kang; Do-Hwan Ahn; Jun Hyuck Lee; Sung Gu Lee; Seung Chul Shin; Jungeun Lee; Gi-Sik Min; Hyoungseok Lee; Hyun-Woo Kim; Sanghee Kim; Hyun Park GigaScience

Dear Hans,

We would like to thank you and the reviewers for your kind help to revise our manuscript and consider our manuscript for publication in GigaScience. We appreciated all of the comments and suggestions and carefully considered all of them during the revision. All of inferred statements have been corrected and we have included the photo of the specimen as a main figure as you suggested. The corrected points were marked in blue color in revised manuscript.

We did our best to address the comments from the reviewers. Hope the revised is acceptable for publication. We look forward to hearing your decision.

Thanks and best regards.

Reviewer reports:
Reviewer #1: This paper is in reasonably good shape and the data will be useful for comparison to temperate Tigriopus. My only remaining comment is that the coverage of the CEGMA is relatively low and I don't know how that impacts the estimates of turnover in gene families (which the authors estimate to be quite high). Could this be a result of the fact that the assembly is missing a sizable proportion of core eukaryotic genes?

Response) We have added table 6 which summarizes genome completeness reports of T. kingsejongensis and other arthropod genomes used in gene family assignments. Overall, non-insect arthropod genomes showed relatively low CEGMA and BUSCO assignment scores. This tendency is commonly observed in non-insect genomes [1, 2] because the gene sets are mainly made of widely studied insect genomes. In “Annotation” and “Gene Families” part (which is in blue color), we made comment about this tendency and need to be careful to examine gene turnover events in non-insect arthropod genomes. Finally, we remarked necessity of globally approved arthropod orthologous gene sets to the field.

1. Hoy M, Waterhouse R, Wu K, Estep A, Ioannidis P, Palmer W, Pomerantz A, Simão F, Thomas J, Jiggins F: Genome sequencing of the phytoseiid predatory mite Metaseiulus occidentalis reveals completely atomised Hox genes and super-dynamic intron evolution. Genome biology and evolution 2016.

2. Simão FA, Waterhouse RM, Ioannidis P, Kriventseva EV, Zdobnov EM: BUSCO: assessing genome assembly and annotation completeness with single-copy orthologs. Bioinformatics 2015:btv351.
