# Supplementary material for: The genome of the Antarctic-endemic copepod, Tigriopus kingsejongensis
Source: Gigascience. 2017 Jan 7;6(1):1–9. doi: 10.1093/gigascience/giw010 (PMC5467011; doi:10.1093/gigascience/giw010)
Supplement: Response_to_Review_Comments_Revision_3.pdf [file giw010_Response_to_Review_Comments_Revision_3.pdf]

Dear Hans,

We have tried to address all the comments and we have uploaded both clean and comment version of manuscripts.

Thanks and best regards.
