# Supplementary material for: The genome of the Antarctic-endemic copepod, Tigriopus kingsejongensis
Source: Gigascience. 2017 Jan 7;6(1):1–9. doi: 10.1093/gigascience/giw010 (PMC5467011; doi:10.1093/gigascience/giw010)
Supplement: Resposne_to_Reviewer_Comments_Revision_1.pdf [file giw010_Resposne_to_Reviewer_Comments_Revision_1.pdf]

GIGA-D-16-00040
The genome of Antarctic-endemic Copepod, Tigriopus kingsejongensis
Seunghyun Kang; Do-Hwan Ahn; Jun Hyuck Lee; Sung Gu Lee; Seung Chul Shin; Jungeun Lee; Gi-Sik Min; Hyoungseok Lee; Hyun-Woo Kim; Sanghee Kim; Hyun Park GigaScience

Reviewer reports:
Reviewer #1: This paper is in reasonably good shape and the data will be useful for comparison to temperate Tigriopus. My only remaining comment is that the coverage of the CEGMA is relatively low and I don't know how that impacts the estimates of turnover in gene families (which the authors estimate to be quite high). Could this be a result of the fact that the assembly is missing a sizable proportion of core eukaryotic genes?

Response) We have added BUSCO completeness results and two supplementary tables (Table S4 about CEGMA results and Table S5 about BUSCO results) in data description line 132.
