# Supplementary material for: The genome of the Antarctic-endemic copepod, Tigriopus kingsejongensis
Source: Gigascience. 2017 Jan 7;6(1):1–9. doi: 10.1093/gigascience/giw010 (PMC5467011; doi:10.1093/gigascience/giw010)
Supplement: Reviewer_1_Report_(Original_Submission).pdf [file giw010_Reviewer_1_Report_(Original_Submission).pdf]

Reviewer Report

Title: "The genome of the Antarctic-endemic copepod, Tigriopus kingsejongensis"

Version: Original Submission **Date:** 7/29/2016

Reviewer name: Ronald Burton

Reviewer Comments to Author:

The paper describes the genome of a recently described species of the harpacticoid genus Tigriopus. The species is interesting as it lives in the cold Antarctic environment. If published, it may well be the first paper describing a copepod genome; however, it should be noted that other copepod genomes are already available online, including the congener Tigriopus californicus (https://i5k.nal.usda.gov/Tigriopus_californicus). Some reference to this and other copepod genomes (Eurytemora affinis and Salmon louse) might be appropriate.

The abstract and background of this paper both open with what is clearly a false statement - there are not more species of copepods than insects or nematodes; there are over 1 million species of insects vs. ~12,000 species of copepods, so it's not clear what the authors are stating. Perhaps this is a language problem, but it results in a very significant error.

Background line 24 - outdated refs for temperature adaptation. Full transcriptome response to heat stress in T. californicus was published 4 years ago (Schoville et al 2012 BMC Evolutionary Biology) and would seem to be an especially appropriate reference here as the authors could contrast response to cold with the response to heat. (also consider Barreto et al. 2011 Interpopulation patterns of divergence and selection across the transcriptome of the copepod Tigriopus californicus. Molecular Ecology. 20:560-572). It might be interesting to see if there is any overlap between genes identified as under positive selection in this species versus those identified as under selection between T. californicus populations (see Pereira et al. 2016 Molecular Ecology).

As for the genome data itself, the assembly reported is interesting as the genome size is substantially larger than that of T. californicus (size based on nuclear fluorescence is ~240Mb). The assembly is rather fragmented >11,000 scaffolds and I wonder if they might see significant improvement if they used a different assembler (maybe try ALLOPATHS instead of Celera - they have the necessary data)? The completeness statistic based on coverage of CEGMA is not great (82%) but they used the larger set of 458 conserved proteins rather than the more conservative set of 248 proteins often used. The authors do not report what percentage of the genes are fully (vs. partially) covered in their assembly - this might make it easier for readers to better interpret the results. I think this may be an issue if their assembly is

rather incomplete in total coverage, the reported gains and losses of gene families may be unreliable. The number of annotated gene models 12,772 is a bit low (10% lower than the smaller T. californicus genome at ~14,100).

The authors present a variety of interesting analyses regarding adaptation to cold. The role of trehalose transporters, presents a testable hypothesis since RNAi methods are available in Tigriopus (Barreto et al 2015, Molecular Ecology Resources).

Overall this is an interesting paper! With some polishing, it is certainly appropriate for publication in GigaScience. Congratulations to the authors.

Level of Interest

Please indicate how interesting you found the manuscript: An article whose findings are important to those with closely related research interests

Quality of Written English

Please indicate the quality of language in the manuscript: Needs some language corrections before being published

Declaration of Competing Interests

Please complete a declaration of competing interests, considering the following questions:

- Have you in the past five years received reimbursements, fees, funding, or salary from an organisation that may in any way gain or lose financially from the publication of this manuscript, either now or in the future?
- Do you hold any stocks or shares in an organisation that may in any way gain or lose financially from the publication of this manuscript, either now or in the future?
- Do you hold or are you currently applying for any patents relating to the content of the manuscript?
- Have you received reimbursements, fees, funding, or salary from an organization that holds or has applied for patents relating to the content of the manuscript?
- Do you have any other financial competing interests?
- Do you have any non-financial competing interests in relation to this paper?

If you can answer no to all of the above, write 'I declare that I have no competing interests' below. If your reply is yes to any, please give details below.

My lab has sequenced the genome of a congener, Tigriopus californicus, and made the assembly publicly accessible over a year ago on a US government sponsored website (https://i5k.nal.usda.gov/Tigriopus_californicus). Our manuscript describing the work is still a month or more from submission.

I agree to the open peer review policy of the journal. I understand that my name will be included on my report to the authors and, if the manuscript is accepted for publication, my named report including any attachments I upload will be posted on the website along with the authors' responses. I agree for my report to be made available under an Open Access Creative Commons CC-BY license (http://creativecommons.org/licenses/by/4.0/). I understand that any comments which I do not wish to be included in my named report can be included as confidential comments to the editors, which will not be published.

I agree to the open peer review policy of the journal
