# Supplementary material for: The genome of the Antarctic-endemic copepod, Tigriopus kingsejongensis
Source: Gigascience. 2017 Jan 7;6(1):1–9. doi: 10.1093/gigascience/giw010 (PMC5467011; doi:10.1093/gigascience/giw010)
Supplement: Reviewer_2_Report_(Original_Submission).pdf [file giw010_Reviewer_2_Report_(Original_Submission).pdf]

Reviewer Report

Title: "The genome of the Antarctic-endemic copepod, Tigriopus kingsejongensis"

Version: Original Submission **Date:** 8/30/2016

Reviewer name: Carol Eunmi Lee, Ph.D.

Reviewer Comments to Author:

This study focuses on an interesting topic regarding the genome sequence of a copepod from an extreme habitat. I was initially intrigued by the topic and approached it with great interest, as I have an inherent interest in environmental adaptations, especially by copepods.

However, I quickly became a bit perturbed. The authors claim that genomes and genomic resources are lacking for copepods, and that this is the first copepod genome paper. This is an odd statement, given that there are more genomes freely available for copepods than for any other crustacean, and a plethora of genomic resources available, relative to other non-insect arthropods. The salmon louse genome project had an official press release five years ago, and is available for analysis. In addition, two other copepod genomes, those of Tigriopus californicus and Eurytemora affinis, are freely available from the Arthropod i5K website, and have been available for over two years. And this study does make a comparison with the genome of Tigriopus japonicus, which has been around for a while. I have seen some comparative studies that incorporate analyses of these other copepod genomes, without calling them the "first genome." There are also many copepod transcriptomes freely available.

Given the availability of the other copepod genomes, this study would benefit from comparisons with at least the congener Tigriopus californicus, in addition to the congener T. japonicus.

Here is a paper on the genome of Tigriopus japonicus:
https://www.researchgate.net/publication/40898763_The_copepod_Tigriopus_japonicus_genomic_DNA_information_574_Mb_and_molecular_anatomy

The genome of Tigriopus californicus is available at: https://i5k.nal.usda.gov/Tigriopus_californicus

SNP and linkage map of T. californicus: http://bmcgenomics.biomedcentral.com/articles/10.1186/1471-2164-12-568

Also, the salmon louse genome project:
http://www.imr.no/nyhetsarkiv/2010/november/lakselusens_arvestoff_er_sekvensert/en

Salmon louse genome made public in 2011: http://www.atlanticsalmontrust.org/latest-news/salmon-louse-genome-study-results-to-be-made-public-170.html

The reporting of Results and Discussion in this study is quite minimal, with no synthesis. More effort should be devoted to describing what the results are and explaining what they mean. A two paragraph Discussion that fails to explain most of the Results is not adequate. Most of the useful figures and tables are thrown into the supplementary files, without much explanation. And the figures included in the text are not that informative, with minimal explanation of what they mean. At this point there is not much to evaluate in this draft, as there isn't much content in the paper yet.

This study focuses on two seemingly disjoint pieces of data with no connection made between them. The authors look for genome-wide signatures of selection across the genome and discover many transport genes. Then they look for differentially expressed genes for this copepod in response to two temperatures. ***So, do any of the genes that show differential expression in response to two different temperatures also show signatures of selection???

For instance, the findings on the TkTret genes are interesting, where they show differential expression between two different temperatures and exhibit an expansion of 7 paralogs (as opposed to 4 in other taxa). Do any of these paralogs show signatures of selection?

The authors applied PAML to test for signatures of selection. I recommend that they also use HyPhy, which is more powerful, and able to make greater inferences than PAML.

Please go through the paper and correct the spelling and grammatical errors.

This study has the potential to make a valuable contribution to the field, but much more work needs to be devoted toward finishing the paper.

Level of Interest

Please indicate how interesting you found the manuscript: An article whose findings are important to those with closely related research interests

Quality of Written English

Please indicate the quality of language in the manuscript: Not suitable for publication unless extensively edited

Declaration of Competing Interests

Please complete a declaration of competing interests, considering the following questions:

- Have you in the past five years received reimbursements, fees, funding, or salary from an organisation that may in any way gain or lose financially from the publication of this manuscript, either now or in the future?
- Do you hold any stocks or shares in an organisation that may in any way gain or lose financially from the publication of this manuscript, either now or in the future?
- Do you hold or are you currently applying for any patents relating to the content of the manuscript?
- Have you received reimbursements, fees, funding, or salary from an organization that holds or has applied for patents relating to the content of the manuscript?
- Do you have any other financial competing interests?
- Do you have any non-financial competing interests in relation to this paper?

If you can answer no to all of the above, write 'I declare that I have no competing interests' below. If your reply is yes to any, please give details below.

I declare that I have no competing interests

I agree to the open peer review policy of the journal. I understand that my name will be included on my report to the authors and, if the manuscript is accepted for publication, my named report including any attachments I upload will be posted on the website along with the authors' responses. I agree for my report to be made available under an Open Access Creative Commons CC-BY license (http://creativecommons.org/licenses/by/4.0/). I understand that any comments which I do not wish to be included in my named report can be included as confidential comments to the editors, which will not be published.

I agree to the open peer review policy of the journal
